# Androgen receptor-modulatory microRNAs provide insight into therapy resistance and therapeutic targets in advanced prostate cancer

**DOI:** 10.1038/s41388-019-0823-5

**Published:** 2019-05-01

**Authors:** Claire E. Fletcher, Eric Sulpice, Stephanie Combe, Akifumi Shibakawa, Damien A. Leach, Mark P. Hamilton, Stelios L. Chrysostomou, Adam Sharp, Jon Welti, Wei Yuan, Dafydd. A. Dart, Eleanor Knight, Jian Ning, Jeffrey C. Francis, Evangelia E. Kounatidou, Luke Gaughan, Amanda Swain, Shawn E. Lupold, Johann S. de Bono, Sean E. McGuire, Xavier Gidrol, Charlotte L. Bevan

**Affiliations:** 1 Imperial Centre for Translational and Experimental Medicine, Department of Surgery & Cancer, Imperial College London, Hammersmith Hospital, Du Cane Road, London, W12 0NN UK; 2 Université Grenoble Alpes, CEA, INSERM, BIG, BGE, 17 Avenue des Martyrs, 38054 Grenoble, France; 3 Department of Molecular and Cellular Biology, Baylor College of Medicine, 1 Baylor Plaza Houston M822, Houston, TX 77030 USA; 4 Prostate Cancer Target Therapy Group, Institute of Cancer Research and The Royal Marsden NHS Foundation Trust, Sutton, UK; 5 Tumour Profiling Unit, Institute of Cancer Research, London, SW3 6JB UK; 6 Northern Institute for Cancer Research, Newcastle University, Newcastle upon Tyne, NE2 4HH UK; 7 The James Buchanan Brady Urological Institute, Johns Hopkins University School of Medicine, Baltimore, MD USA; 8 Department of Molecular and Cell Biology, Baylor College of Medicine Hospital, Houston, TX USA; 9 Dan L. Duncan Cancer Center, Baylor College of Medicine, Houston, TX USA; 10 Department of Radiation Oncology, The University of Texas MD Anderson Cancer Center, Houston, TX USA

**Keywords:** Non-coding RNAs, Prostate cancer

## Abstract

Androgen receptor (AR) signalling is a key prostate cancer (PC) driver, even in advanced ‘castrate-resistant’ disease (CRPC). To systematically identify microRNAs (miRs) modulating AR activity in lethal disease, hormone-responsive and -resistant PC cells expressing a luciferase-based AR reporter were transfected with a miR inhibitor library; 78 inhibitors significantly altered AR activity. Upon validation, miR-346, miR-361-3p and miR-197 inhibitors markedly reduced AR transcriptional activity, mRNA and protein levels, increased apoptosis, reduced proliferation, repressed EMT, and inhibited PC migration and invasion, demonstrating additive effects with AR inhibition. Corresponding miRs increased AR activity through a novel and anti-dogmatic mechanism of direct association with AR 6.9 kb 3′UTR and transcript stabilisation. In addition, miR-346 and miR-361-3p modulation altered levels of constitutively active AR variants, and inhibited variant-driven PC cell proliferation, so may contribute to persistent AR signalling in CRPC in the absence of circulating androgens. Pathway analysis of AGO-PAR-CLIP-identified miR targets revealed roles in DNA replication and repair, cell cycle, signal transduction and immune function. Silencing these targets, including tumour suppressors ARHGDIA and TAGLN2, phenocopied miR effects, demonstrating physiological relevance. MiR-346 additionally upregulated the oncogene, YWHAZ, which correlated with grade, biochemical relapse and metastasis in patients. These AR-modulatory miRs and targets correlated with AR activity in patient biopsies, and were elevated in response to long-term enzalutamide treatment of patient-derived CRPC xenografts. In summary, we identified miRs that modulate AR activity in PC and CRPC, via novel mechanisms, and may represent novel PC therapeutic targets.

## Introduction

Prostate cancer (PC) is the most prevalent malignancy in Western males [1]. Initially, growth of prostate tumours is dependent on circulating androgens activating the androgen receptor (AR) [2]. Targets of this ligand-activated transcription factor include effectors of cell cycle progression and proliferation, hence androgen deprivation remains a first-line approach for treatment of metastatic PC. However, relapse almost invariably occurs as tumours progress to refractory and largely lethal ‘castrate-resistant’ status (CRPC). Despite the lack of requirement of CRPC tumours for circulating androgens, it is widely accepted that AR signalling is still driving their growth. Mechanisms of persistent AR activity include increased sensitivity to remaining androgens (e.g. adrenal androgens), ligand promiscuity, AR gene amplification or mutation, altered AR mRNA splicing leading to constitutively active forms, aberrant expression of AR coregulators and intra-tumoural androgen synthesis [3]. Thus compounds reducing AR levels and/or activity are likely to be effective in CRPC, for which treatment options are severely limited. Hence, Abiraterone (Abi – a CYP17A inhibitor with AR-inhibitory activity) and Enzalutamide (Enza – an anti-androgen) are recommended for CRPC treatment [4]. Unfortunately, acquired resistance to drugs targeting the AR ligand binding domain (LBD) is an increasing clinical problem, so novel therapeutics that repress AR through non-LBD-mediated mechanisms will be important additions to the CRPC treatment repertoire.

MiRs are 20–25 bp non-coding RNAs that regulate gene expression through interaction with complementary binding sites in target mRNAs, most commonly in 3′ untranslated regions (3′UTRs). Mammalian miRs are transcribed by RNA polymerase II, generating a primary miR (pri-miR) transcript that undergoes several processing steps to the mature miR by way of a precursor stage (pre-miR), usually requiring the nuclear Drosha- and DiGeorge Critical Region 8 (DGCR8)-containing Microprocessor (MP) complex, the RNase III enzyme, Dicer, and the RNA-induced silencing complex (RISC) [5]. Then, mature miR associates with Argonaute 2 (AGO2) of the RISC and guides the complex to complementary sequences within the 3′-UTR of target mRNAs, usually resulting in translational repression and/or transcript degradation. There is a wealth of evidence for the importance of miRs, several of which can function as tumour-promoting oncogenes (or “oncomiRs”) and also tumour suppressors in hormone-regulated cancers [6–9], in PC. Androgen regulation of numerous miRs has been demonstrated, also miR expression profiles are altered during PC progression, with dysregulation in CRPC compared to androgen-dependent tumours [7, 10–14]. Their roles in PC likely include altering androgen signalling via targeting of AR itself, AR target genes or AR coregulatory proteins, opening up multiple avenues for future therapeutic development. However, surprisingly little is known concerning the repertoire of miRs that modulate AR activity.

Previously, Östling et al. [15] used a pre-miR library to perform gain-of-function screening for AR-modulatory miRs in PC cell lines. Thirteen miRs were validated as significantly reducing AR 3′-UTR activity: miR-135b, miR-185, miR-297, miR-299-3p, miR-34a, miR-34c, miR-371-3p, miR-421, miR-449a, miR-449b, miR-634, miR-654-5p and miR-9 [15]. AR targeting by miR-185 was also shown by Qu et al. [16], who demonstrated that miR-185 significantly reduces AR protein (but not mRNA) in LNCaP cells, and decreases LNCaP proliferation and xenograft tumourigenicity. AR targeting by miR-34a was previously shown by Kashat et al. [17], in C4-2B and CWR22Rv-1 cells. Other miRs also target AR directly: Hagman et al. [18] demonstrated ectopic miR-205 overexpression to significantly decrease AR protein levels and 3′-UTR activity in VCaP cells, although this was not recapitulated in LNCaP cells. In addition, miR-205 levels were low in all but one PC cell line, so the physiological relevance in PC is in question. Similarly, AR mRNA and protein levels, AR transcriptional activity and AR 3′UTR reporter activity were all decreased following ectopic expression of miR-488-5p in androgen-dependent and -independent PC cells [19]. In 2016, comprehensive, complementary functional screening for AR activity-regulating miRs, using a library of miR mimics [13] highlighted miR-30 family members as directly inhibiting AR activity through 3′UTR association; it also showed them to reduce PCa cell proliferation and inversely correlate with mRNA levels of the AR target gene PSA in clinical samples. This work also corroborated miR-488-5p as an AR-targeting miR. Our work complements the above miR mimic screening approach through use of a miR inhibitor library in androgen-dependent and -independent PC cell lines. A miR inhibitor library was selected since, unlike miR mimics, inhibitors do not deplete cells of endogenous miR processing or effector complexes (such as RISC), which could lead to off-target or non-specific effects.

Indirect regulation of AR by miRs is also evident in PC cells. For example, the tumour-suppressive miR, let-7c, reduces AR activity and PC cell growth by repressing the oncogenes Ras and c-Myc [20, 21], as Myc is required for AR transcriptional activity [22]. Let-7c expression is frequently reduced, or its gene deleted, in cancer [12, 23]. Similarly, miR-331-3p reduces AR activity in several PC cell lines by targeting the oncogene ERBB2 [24], which can activate transcription of AR target genes in the absence of androgens [25]. Tumour-suppressive, AR-inhibitory miRs such as these could be exploited as therapeutics for PC, alone or in combination with anti-androgens.

The above findings demonstrate the importance of miRs in regulation of AR activity, both directly and indirectly. However, much remains to be learnt surrounding mutual regulation of miRs and AR. Thus we sought a miRnome-wide approach to identify AR-modulatory miRs that may represent novel therapeutics, drug targets or biomarkers in PC. We used LNCaP and C42 cell lines to model androgen-dependent and CRPC stages of the disease respectively, with expression of an integrated luciferase-based AR activity reporter as an easily-assayable end-point [26]. To perform a non-biased screen with high coverage of the potential miR repertoire, we transfected these with a library comprising LNA-modified antisense inhibitors against 983 human miRs. Importantly, this strategy identified not only miRs that target AR directly, but also those that modulate AR activity indirectly by targeting AR cofactors, chaperones, pioneer factors and effector proteins.

## Results

### Identification and validation of AR-modulatory MiRs

**Fig. 1:**
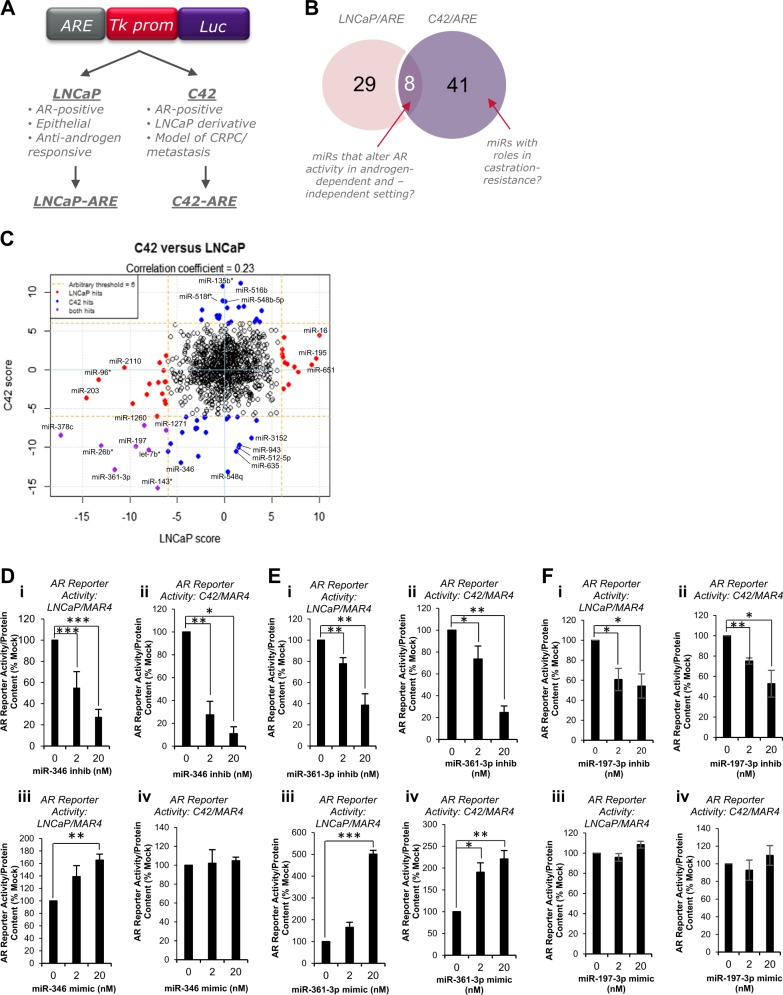
High-throughput microRNA inhibitor screening identified microRNAs that modulate androgen receptor signalling in prostate cancer (PC) cells. **a** Schematic illustration of AR reporter construct and derivative cell lines used for miR inhibitor screening. **b** Venn diagram illustrating common hits between LNCaP and C42 cells. **c** Correlation of miR hit scores between LNCaP and C42 cells. **d**–**f** Luciferase assay analysis of AR activity in LNCaP (i, ii) and C42 (iii, iv) cells transfected with 0–20 nM miR-346 (**d**), miR-361-3p (**e**) or miR-197-3p (**f**) inhibitor (i, iii) or mimic (ii, iv) for 24 h. Mean of three independent experiments ± SEM is shown. *P ≤ 0.05, **P ≤ 0.005, ***P ≤ 0.0001. See also Supplementary Table 2

**Fig. 2:**
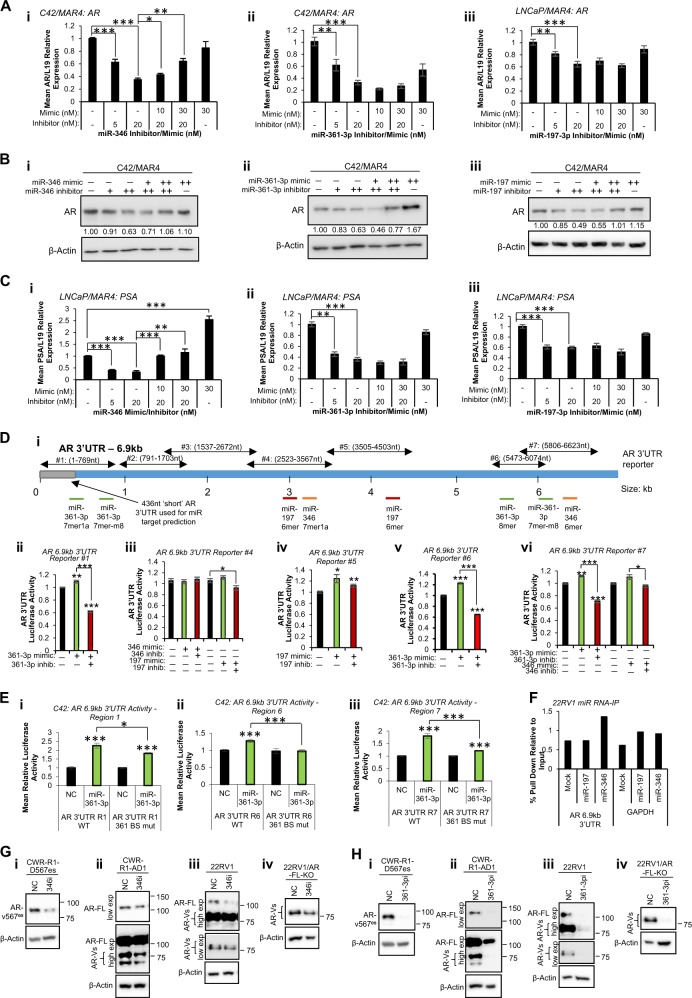
MiR-346, -361-3p and -197-3p alter wild-type and variant androgen receptor activity in prostate cancer (PC) cells partially through association with AR 3′UTR. **a** qRT-PCR analysis of AR mRNA levels in C42/MAR4 cells transfected with (i) miR-346, (ii) miR-361-3p or (iii) miR-197-3p mimic and/or inhibitor for 24 h. **b** Western blot analysis of AR protein levels in C42/MAR4 transfected with (i) miR-346, (ii) miR-361-3p or (iii) miR-197-3p mimic and/or inhibitor for 24 h. β-actin was used as a control for loading. Representative blots of three independent experiments are shown. Additional biological replicates and densitometry for three independent experiments are shown (Fig. S1c–g). **c** qRT-PCR analysis of PSA mRNA levels in LNCaP/MAR4 cells transfected with (i) miR-346, (ii) miR-361-3p or (iii) miR-197-3p mimic and/or inhibitor for 24 h. L19 was used as a normalisation gene. **d**, **e** Luciferase assay analysis of 6.9 kb AR 3′UTR activity in HEK293T (**d**) or C42 (**e**) cells transfected with pMiR-Report vector containing WT (**d**) or miR binding site-mutant (**e**) regions of the AR 6.9 kb 3′UTR as depicted (Fig. S2Di) ± miR mimics or inhibitors (20 nM) for 48 h (**d**) or 72 h (**e**) Luciferase activity was normalised to β-galactosidase activity to correct for transfection efficiency. Columns: mean normalised AR reporter luciferase activity from three independent experiments performed in duplicate ± SEM. **f** AGO2/biotin-miR RNA-IP analysis of miR-197 and miR-346 association with AR 6.9 kb 3′UTR. 22RV1 cells transfected with biotin-labelled miR (200 pmol) for 24 h, followed by two-step immunoprecipitation with AGO2 antibody- and streptavidin-coated beads. RNA was extracted from input and IP samples and qRT-PCR performed for 6.9 kb AR 3′UTR. Data are presented relative to input values. **g**, **h** Western blot analysis of wild-type AR (110 kDa) and variant-AR (65–90 kDa) in (i) CWR-R1-D567es (lacking WT-AR but overexpressing v567^es^), (ii) CWR-R1-AD1 (parental cells expressing WT- and variant-AR), (iii) 22RV1 (expressing WT- and variant-AR) and (iv) 22RV1/AR-FL-KO cells (expressing AR-variants but with WT-AR knocked out) cells transfected with (**a**) miR-346 or (**b**) miR-361-3p inhibitor (both 20 nM) for 24 h. β-actin was used as a control for loading. Representative western blot images are shown. See Fig. S5 for independent biological replicates. *P ≤ 0.05, **P ≤ 0.005, ***P ≤ 0.0001. See also Figs. S1, S2, S3, S4 and S5

In order to identify miRs that modulate AR activity in androgen-dependent and -independent PC, LNCaP and C42 cells stably expressing a luciferase-based AR activity reporter [26] (Fig. 1a) were transfected with a library of 983 miR inhibitors. Luciferase assays were performed and B Score calculated. Using a stringent B score of ±6, 78 miR inhibitors were found to significantly modulate AR reporter activity (B Score ≥ 6 in at least one PC cell line), 63% negatively and 37% positively. B score correlation between LNCaP and C42 cells, and top 30 hits are shown (Fig. 1c, Table S2, respectively). Eight miR inhibitors significantly altered AR activity in the same direction in both LNCaP and C42 cells (B Score ≥ 6, Fig. 1b, Table S2), while no inhibitors significantly modulated AR activity in opposite directions in the two cell lines at B Score ≥6. MiR-346, -361-3p and -197-3p were selected from top ten ‘hits’ due to their high B scores and confirmed expression in cell lines (Ct < 35 in LNCaP cells). To confirm the ability of these miRs to regulate AR activity in androgen-dependent and -independent settings, inhibitors or mimics were transfected into LNCaP/ARE and C42/ARE and luciferase assays performed. Figure S1a confirms significantly increased miR levels in C42 cells transfected with miR mimics. MiR-346 inhibitor significantly reduced AR activity by up to 90% in a dose-dependent manner in LNCaP/ARE and C42/ARE cells (Fig. 1di, ii). AR activity was not altered following transfection of a non-targeting inhibitor (Fig. S1b). Conversely, miR-346 mimic increased AR activity by 70% in LNCaP/ARE cells, an effect not significant in C42/ARE cells (Fig. 1diii, iv). Similarly, miR-361-3p inhibitor significantly repressed AR activity by up to 80% in both cell lines (Fig. 1ei, ii), while miR-361-3p mimic significantly increased AR activity by up to fivefold (Fig. 1eiii, iv). Comparable data were obtained for miR-197 inhibitor, which decreased AR activity by up to 50% (Fig. 1fi, ii), although miR-197 mimic did not significantly increase AR activity in either cell line (Fig. 1fiii, iv). Further, miR mimics are able to rescue effects of miR inhibitors, verifying that these are not off-target effects (Fig. 2a, c).

### Modulation of MiR-346, -361-3p and -197 alters AR wild-type and variant mRNA, protein and 3′UTR activity

Given that modulating miR-346, -361-3p and -197 significantly altered AR activity, we hypothesised that this may be due to changes in AR mRNA or protein levels. Thus, C42 and LNCaP cells were transfected with inhibitor and/or mimic and AR mRNA and protein levels assessed 24 h post-transfection. Non-targeting miR inhibitors and mimics did not significantly modulate AR activity in either cell line (Fig S1b). It was shown that inhibition of each miR under investigation significantly reduced AR mRNA levels by up to 70% (Fig. 2a, Fig. S1c), and that addition of mimic in the presence of inhibitor could rescue AR mRNA loss in a dose-dependent manner, an effect significant for miR-346 (Fig. 2ai). Further, inhibition of miR-346, -361-3p and -197 significantly reduced AR protein levels in a dose-dependent manner, also rescued through addition of miR mimic (Fig. 2b, Fig. S1d–h). Similar effects were observed in LNCaP cells (Fig. S1f, g, h).

To examine effects of miR manipulation on AR transcriptional output, we assessed levels of the endogenous AR target genes, *PSA*, *TMPRSS2* and *DRG1* in both LNCaP and C42 cells. Inhibition of miR-346, -361-3p or -197 was found to significantly reduce PSA mRNA levels by up to 75% in LNCaP cells (Fig. 2c). Loss of PSA mRNA was rescued through addition of miR-346 mimic, and miR-346 mimic alone was found to significantly increase PSA mRNA levels compared to mock-transfected cells (Fig. 2ci). Similar results were obtained for other AR target genes in both LNCaP and C42 (Fig. S2a–d). To assess whether upregulation of AR activity and protein levels occurs through direct miR activity at the AR 3′UTR, we analysed AR 3′UTR for miR-346, -361-3p and -197 seed region complementarity. Although algorithm-based miR binding prediction tools such as microrna.org and DIANAmicroT predict miR associations with an AR 3′UTR of 436 nt and c. 3 kb, respectively, a number of studies have identified AR 3′UTR lengths of between 6.6 and 6.9 kb in PC cells [27] resulting from alternative polyadenylation [15], meaning that large numbers of biologically important potential miR: AR 3′UTR interactions may be missed during bioinformatic analysis. Thus, 6.8 Kb AR 3′UTR sequence (from NM_000044 was examined for miR binding sites). Two miR-361-3p binding sites (complete seed complementarity) were identified within the proximal region of AR 6.8 kb 3′UTR at nucleotides 407–412 and 787–793 (although only the first is within the 436 nt short AR 3′UTR), with a further two sites identified distally at 5772–5777 and 6070–6075 (Fig. 2di). MiR-346 binding sites were identified at 3185–3190 and 6283–6288, and miR-197 binding sites at 3043–3048 and 4308–4313, none within the standard short AR 3′UTR. All sites were relatively poorly conserved across species, with the exception of the miR-197 site at 4308–4313, which was completely conserved across almost all mammals (Fig. S3).

To examine the functionality of these regions of seed complementarity, we performed luciferase assays in HEK293T cells using reporter vectors containing seven overlapping regions of AR 6.8 kb 3′UTR downstream of a luciferase gene (Fig. 2d, e) [13]. Effects of miR-361-3p on the 787–793 region were not assessed as the complete sequence of this region lies between sequences found in AR 3′UTR reporters #1 and #2. In contrast to the predominant repressive effects usually observed for miRs at 3′UTRs, we found that miR-361-3p increased activity of AR 3′UTR reporters #1, #6 and #7 (all of which contain putative miR-361-3p binding sites) (Fig. 2dii, v, vi), while addition of the corresponding inhibitor significantly reduced AR 3′UTR activity (Fig. 2di, iv, v). Interestingly, miR-346 modulation had no effect on activity of AR 3′UTR reporter #4, despite this region containing a miR-346 7mer1a site (Fig. 2diii). Likewise, AR 3′UTR reporter #4 activity was only minimally increased by addition of miR-197 (although significantly decreased by miR-197 inhibitor), despite containing a miR-197 6mer site (Fig. 2diii). MiR-197 increased luciferase activity of AR 3′UTR reporter #5, which was partially rescued by addition of miR-197 inhibitors (Fig. 2div). Finally, miR-346 slightly increased activity of AR 3′UTR reporter #7, an effect abrogated through addition of inhibitor (Fig. 2dvi). Addition of miR-346 to HEK293T cells transfected with AR 3′UTR reporter 2 or 3 (no putative miR-346 binding sites) did not alter luciferase activity, as expected (Fig. S2E). Further, above effects of miR-361-3p addition were replicated in C42 cells (Fig. 2e). The observation that miR-361-3p produces a greater increase in region 1 and 7 AR 3′UTR activity in C42 cells (Fig. 2ei, iii) as compared to HEK293T cells (Fig. 2dii, vi) may reflect the increased importance of this mode of AR regulation in AR-requiring PC cells as compared to non-cancer lines.

To confirm specificity of interaction of AR-modulatory miRs with identified seed region complementary sequences (Fig. 2d), key residues within miR binding sites were mutated in those AR 3′UTR reporters that demonstrated altered activity upon miR transfection. MiR-361-3p induction of AR 3′UTR activity was significantly reduced upon mutation of miR-361-3p binding sites of reporters #1, #6 and #7 in C42 cells (Fig. 2e), with similar results observed in HEK293T cells (Fig. S2Fiii-v). Interestingly, luciferase activity of neither wild-type nor miR-197 binding site-mutant AR 3′UTR reporter #5 was altered upon miR-197 transfection at the 24 h timepoint (Fig. S2Fi). Mutation of miR-346 binding site in AR 3′UTR reporter #7 reduced miR-346-induced increase in activity of the wild-type vector, albeit non-significantly (Fig. S2Fii). In order to identify physiological association of AR-modulatory miRs with AR transcript, we performed AGO/biotin-miR RNA-IP, whereby biotin-labelled miR mimic-transfected cells are subjected to a two-step IP with anti-AGO2 antibody, followed by streptavidin IP, RNA extraction and qRT-PCR. Minimal enrichment of AR transcript was identified in presence of miR-197 or -346, and AR enrichment levels were similar to those observed for the abundant house-keeping gene GAPDH, which lacks predicted miR-346 and -197 binding sites (Fig. 2f). In agreement with this observation, AGO-PAR-CLIP did not identify AR as a target of either miR-346 or -197(36), although this may be due to use of standard AR 3′UTR sequence length upon read alignment during data processing. Nevertheless, we hypothesised that miR:AR interaction may be very transient, and thus undetectable by RNA-IP methodologies. Alternatively, it is possible that miRs may stabilise AR transcript in an AGO2-independent manner.

A potential mechanism of persistent cell survival under conditions of depleted circulating androgens in CRPC is expression of AR variants (AR-Vs) that are constitutively active, lacking the LBD but retaining N-terminal transactivation and DNA-binding domains [28]. AR variants, such as AR-v7 and AR-v567^es^, drive overlapping but distinct gene sets to wild-type AR [28]. AR-v1, -v4, -v7 and -v9 lack the C-terminal exon (exon 8), and thus have entirely distinct 3′UTRs from WT transcript. These variants lack the miR-346 and -361-3p binding sites present in WT-AR 3′UTR, but contain an additional variant-specific miR-361-3p site (Table S3). In contrast, ARv567^es^ lacks exons 5–7 of the LBD, but retains exon 8 (and an additional exon 9). It thus has a 3′UTR longer than, but with high sequence similarity to, WT-AR, retains WT miR-346 and -361-3p binding sites and also has an additional miR-346 site at the 3′ end of its 3′UTR (Table S3). To assess effects of miR-346 and -361-3p on AR variant mRNA levels, we used 22RV1 cells, which express the majority of described AR variants, and C42 cells, which express AR-v7 only. It was found that miR-346 inhibition reduced WT, v7 and v567es expression in 22RV1 cells, with significance reached for AR-v7. Similarly, miR-346 inhibitor significantly decreased AR-WT and -v7 expression in C42 cells (Fig. S4a, b, see also Fig. 2a). Very similar results were obtained for miR-361-3p (Fig. S4c, d). To examine effects of miR-346 and -361-3p inhibition on WT and variant-AR protein levels, Western blotting was performed in 22RV1 cells, WT-knock out derivatives (22RV1-AR-FL-KO—all variants expressed but not WT), parental CWR-R1-AD1 cells (lacking AR-v567es) and the CWR-R1-D567^es^ derivative lacking WT-AR but overexpressing v567^es^. An N-terminal AR antibody was used in order to detect both WT and variant-AR. This antibody is unable to identify individual variants, although AR-v567es is distinguishable from others due to its size (80.7 kDa vs. 50.6–74.9 kDa for variants 1, 4, 7 and 9 and 110 kDa for WT-AR). Of note: the 22rv1 line carries a 35 kb tandem duplication encompassing AR exon 3 and cryptic exons, which are spliced as alternative 3′ exons of AR-variants, including v7 [29]. This may result in increased levels of cryptic exon-derived variants in this cell line, potentially in different ratios compared to cells lacking the duplication. These, as above, are indistinguishable using available antibodies. It was shown that miR-346 inhibitor reduces protein levels of full-length and multiple variants of AR (including v567^es^) across the above panel of cell lines after 24 h (Fig. 2g). MiR-361-3p inhibitor produced similar results, demonstrating a greater extent of repression (Fig. 2h). Biological replicates are shown in Fig. S5. Thus we conclude that miR-346 and miR-361-3p modulation alters levels of constitutively active AR variants in PC, and may contribute to persistent AR signalling in CRPC in the absence of circulating androgens.

### AR-modulatory MiRs stabilise AR transcript and their inhibition demonstrates additive effects with AR silencing

**Fig. 3:**
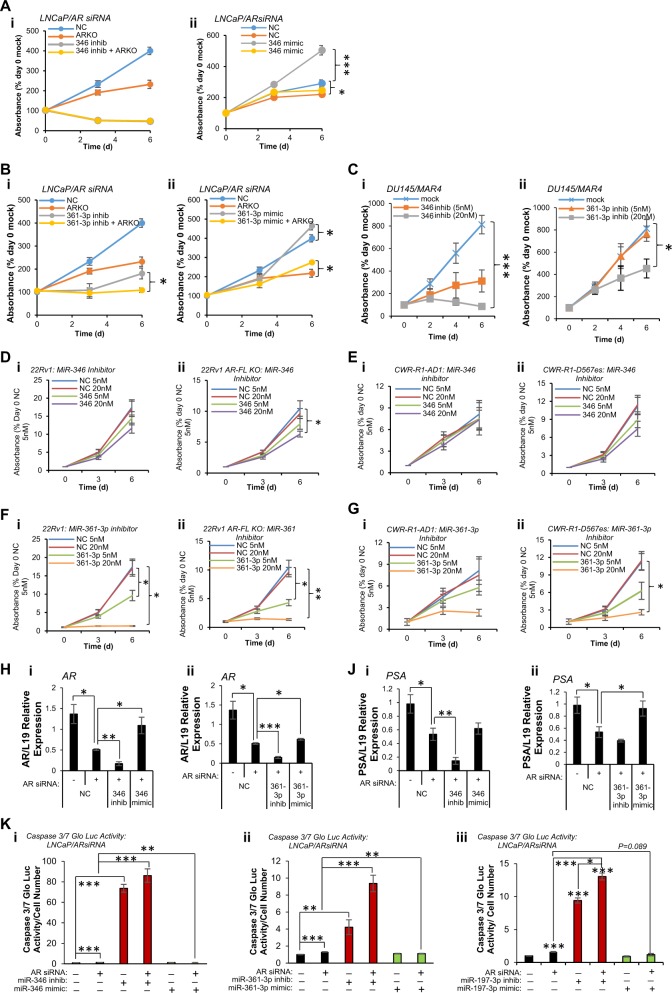
AR-modulatory miRs stabilise AR transcript and regulate apoptosis and WT- and variant-AR-driven proliferation, and MiR-346, -361-3p and -197-3p inhibitors show additive effects with AR silencing. **a**, **b** SRB proliferation assay analysis of LNCaP/ARsiRNA cells transfected with miR-346 (**a**) or miR-361-3p (**b**) mimic (7.5 nM – miR-346, 20 nM – miR-361-3p) or inhibitor (10 nM – miR-346, 20 nM – miR-361-3p) ± 1 µM Dox to induce AR siRNA (ARKO) for 6 days. **c** SRB proliferation assay analysis of DU145 cells transfected with miR-346 (i) or miR-361-3p (ii) inhibitor (5 and 20 nM) for 6 days. **d**–**g** SRB proliferation assay analysis of 22RV1 (**d**i, **f**i), 22RV1-AR-FL-KO (AR variant-driven) (**d**ii, **f**ii), CWR-R1-AD1 (**e**i, **g**i) and CWR-R1-D567^es^ (AR-v567^es^-driven) cells (**e**ii, **g**ii) transfected with: **d**, **e** miR-346 inhibitor (5 and 20 nM) or **f**, **g** miR-361-3p inhibitor (5 and 20 nM) for 6 days. **a**–**g** Data are presented relative to absorbance at day 0. Points: mean absorbance at 492 nm for three independent experiments performed in quadruplicate ± SEM. **h**, **j** qRT-PCR analysis of (**h**) AR and (**j**) PSA mRNA levels in LNCaP/ARsiRNA cells transfected with miR-346 (i) or miR-361-3p (ii) inhibitor or mimic (20 nM) ± Doxycycline (1 µM) for 24 h. L19 was used as a normalisation gene. Columns: mean ± SEM for three independent experiments performed in triplicate. **k** Caspase 3/7 Glo assay analysis of apoptosis in LNCaP/AR siRNA cells ± miR mimic or inhibitor (20 nM) ± Doxycycline (1 µM) for 72 h. Columns: mean relative luminescence for three independent experiments performed in triplicate ± SEM. *P ≤ 0.05, **P ≤ 0.005, ***P ≤ 0.0001. See also Fig. S6

**Fig. 4:**
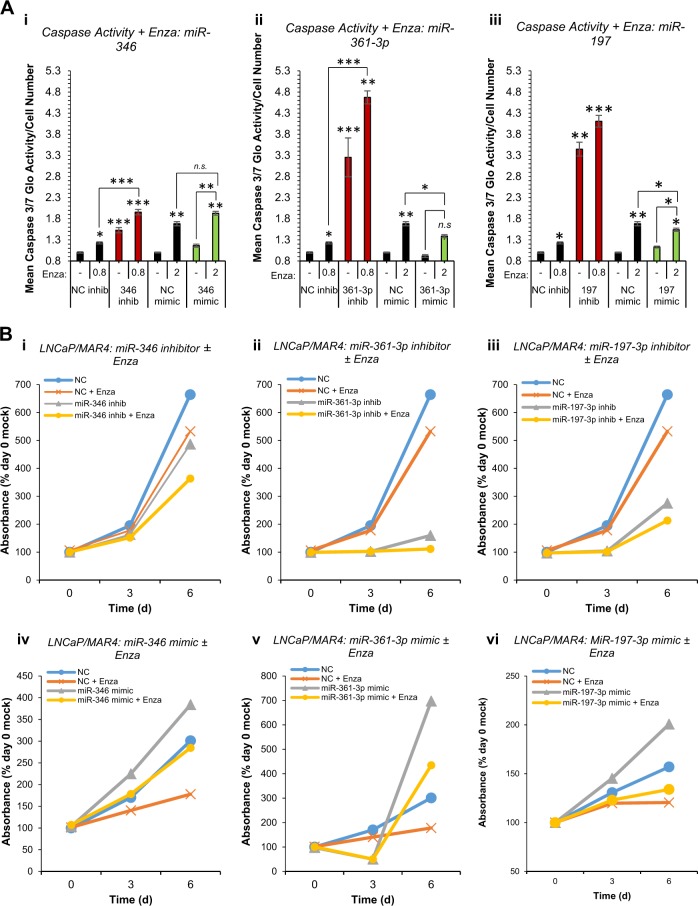
AR-modulatory miRs inhibitors enhance enzalutamide efficacy. **a** Caspase 3/7 Glo assay analysis of apoptosis in LNCaP/MAR4 cells ± miR-346 (i), miR-361-3p (ii) or miR-197-3p (iii) mimic or inhibitor (20 nM) ± Enzalutamide (0.8 µM – in presence of inhibitors, 2 µM in presence of mimics) for 72 h. Columns: mean relative luminescence for three independent experiments performed in triplicate ± SEM. *P ≤ 0.05, **P ≤ 0.005, ***P ≤ 0.0001. **b** SRB proliferation assay analysis of LNCaP/MAR4 cells transfected with inhibitors of miR-346 (i – 2.5 nM), miR-361-3p (ii – 5 nM) or miR-197-3p (iii - 10 nM) or mimics of miR-346 (iv – 10 nM), miR-361-3p (v – 30 nM) or miR-197-3p (vi – 20 nM) ± Enzalutamide (0.8 µM) for 6 days. Data are presented relative to absorbance at day 0 and one representative experiment is shown. Additional biological replicates are shown in Fig. S7

In light of their effects on AR protein levels (Fig. 2b), we investigated the ability of AR-modulatory miRs to enhance AR transcript stability. C42 cells were transfected with miR mimics for 24 h prior to 4 h Actinomycin D (ActD) treatment. In agreement with Fig. 2a, all three miRs increased AR mRNA levels in vehicle-treated conditions compared to negative control (Fig. S2g). It was demonstrated that addition of either miR-346 or miR-197 rescued ActD-mediated loss of AR transcript compared to negative control-transfected cells in a significant manner, while miR-361-3p transfection was not able to rescue ActD-mediated AR mRNA loss (Fig. S2g). Given that miR-346, -361-3p and -197 have profound effects on AR protein levels and activity in PC cells, we hypothesised that modulation of these miRs may alter PC cell proliferation, and that miR inhibition may demonstrate combinatorial effects with AR silencing, potentially revealing a novel therapeutic avenue for PC treatment. To this end, LNCaP cells expressing a doxycycline (Dox)-inducible AR siRNA [30] were treated ± 1 µM Dox and transfected with miR-346 or -361-3p inhibitors or mimics prior to SRB cell proliferation analysis. It was shown that miR-346 inhibitor alone significantly reduced cell number to 40% of day 0 numbers (Fig. 3ai), suggesting that miR-346 inhibition has cytotoxic, rather than cytostatic effects. AR siRNA alone reduced growth of LNCaP cells by ~50% over 6 days (Fig. 3a). Induction of AR siRNA in the presence of miR-346 inhibitor did not enhance effects of miR-346 inhibitor alone, presumably due to the very marked effects of miR-346 inhibitor at concentrations used in this experiment (20 nM) (Fig. 3ai). Converse experiments showed that miR-346 mimic was able to significantly enhance proliferation of LNCaP cells by 80%, and partially rescue effects of AR silencing in a significant manner (Fig. 3aii). Inhibition of miR-361-3p significantly repressed growth of LNCaP cells by 70%, and further addition of AR siRNA in combination repressed cell growth at day 6 to day 0 levels (Fig. 3bi). MiR-361-3p mimic significantly increased proliferation of LNCaP cells and partially rescued AR siRNA-induced reduction in cell growth in a significant manner (Fig. 3bii). It was additionally demonstrated that while miR-346 inhibitor represses C42 and LNCaP proliferation at both 5 nM and 20 nM (Fig. S6a), miR-361-3p inhibitor reduced cell number in a dose-dependent manner in both lines (Fig. S6c). In contrast to the observed increase in LNCaP cell proliferation following transfection of 7.5 nM miR-346 mimic, concentrations of 10 and 50 nM miR-346 mimic significantly reduced proliferation of both C42 and LNCaP cells (Fig. S6b). We hypothesise that this is due to threshold effects of miR-346 manipulation, with lower doses activating proliferative responses, while higher concentrations induce toxicity, leading to apoptotic induction as illustrated in Fig. 4a. Such a threshold is likely to be different in different cell lines. MiR-361-3p mimic significantly increased C42 and LNCaP cell growth at both 10 and 50 nM (Fig. S6d). Interestingly, both miR-346 and -361-3p inhibitors significantly repressed proliferation of AR-negative DU145 cells in a dose-dependent manner (Fig. 3c), suggesting that at least some of miR-346 and -361-3p effects are mediated via non-AR pathways.

We further investigated the ability of miR-346, 1361-3p and -197 to regulate proliferation of AR variant-driven PC cell lines, by comparing proliferation between parental 22RV1 cells (all variants and WT expressed) and WT-knock out derivatives (22RV1-AR-FL-KO), and also between parental CWR-R1-AD1 cells (lacking AR-v567es) and the CWR-R1-D567^es^ derivative lacking WT-AR but overexpressing v567^es^. Interestingly, miR-346 inhibition significantly reduced proliferation of 22RV1 cells expressing variants only, but had less effect on the parental cells with FL AR (Fig. 3d). Although the enhanced effect is modest, it may reflect the additional miR-346 seed complementarity site present in AR-v567^es^ 3′UTR (nts 8353-8358)—see Table S3. This is supported by the observation that miR-346 inhibition non-significantly represses AR-v567es-driven growth of CWR-R1-D567^es^ cells, with no effect observed in parental cells (Fig. 3e). MiR-361-3p inhibition completely blocked proliferation of 22RV1 and the variant-driven derivative, with similar effects in v567es-driven CWR-R1 (Fig. 3f, g). This is consistent with effects in LNCaP, C42 and DU145 cells (Fig. 3a–c, Fig. S6c, d), and presence of similar numbers of miR-361-3p seed complementarity sites in 3′UTRs of AR-variants and -WT (Table S3).

We additionally investigated the ability of AR-modulatory miRs to modulate apoptosis and apoptotic response to AR silencing. It was demonstrated that miR-346, -361-3p and 197 inhibitors significantly enhanced AR siRNA-induced AR loss at transcript level, while AR mRNA was significantly rescued by addition of miR mimics in the presence of AR siRNA (Fig. 3h and Fig. S6e). Accordingly, levels of AR targets PSA, TMPRSS2 and KLK2 were significantly reduced following miR-346, -361-3p of 197 inhibition in the presence of AR siRNA compared to AR siRNA alone, while miR mimics rescued this (Fig. 3j, S6f–h). MiR-346 inhibition significantly increased AR siRNA-induced apoptosis by 85-fold, while miR-346 mimic significantly reduced such apoptosis (Fig. 3ki). Likewise, miR-361-3p significantly increased apoptosis of LNCaP cells by fourfold compared to untreated cells, and significantly enhanced apoptosis induced by AR siRNA by sixfold, with a 50% reduction in apoptosis observed for miR-361-3p mimic compared to AR siRNA alone (Fig. 3kii). In addition, miR-197 inhibitor significantly increased apoptosis tenfold compared to untreated cells, and significantly enhanced AR siRNA-induced cell death by 8.5-fold, whereas miR-197 mimic reduced AR silencing-induced apoptosis by 50% (Fig. 3kiii). Taken together, these data indicate that miR-346 and -197 stabilise AR transcript, and that miR inhibition shows additive effects with AR silencing on proliferation and apoptosis, identifying a rationale for potential combined use of AR-inhibitory therapies and miR-346, -361-3p or -197 inhibitors in PC.

### AR-modulatory MiR inhibition demonstrates additive effects with enzalutamide

To provide further evidence to support the rationale of combined miR-346, -361-3p or -197 inhibitor and anti-androgen treatment, we investigated the effects of AR-modulatory miR manipulation on apoptotic and proliferative response to the anti-androgen, Enzalutamide (Enza), in PC cells. It was demonstrated that miR-346 inhibitor significantly enhances apoptosis induced by Enza by 52%, while miR-346 mimic (20 nM) also increased Enza-induced apoptosis (Fig. 4ai), consistent with miR-346 mimic effects on proliferation in LNCaP cells at 10 and 50 nM (Fig. S6Bii). MiR-361-3p inhibitor significantly increased Enza-induced apoptosis by 4.3-fold, while miR-361-3p mimic significantly reduced Enza-induced apoptosis by 40% compared to negative control-transfected, Enza-treated cells (Fig. 4aii). Similar Enza-induced apoptosis-modulatory effects were observed for miR-197 inhibitor and mimic (Fig. 4aiii). We additionally investigated effects of miR-346, -361-3p and -197 modulation on PC growth inhibition by Enza. Inhibition of miR-346, -361-3p or -197 considerably enhanced Enza-induced reduction in LNCaP proliferation (Fig. 4bi–iii). Conversely, miR-346 and -361-3p mimics were able to completely rescue Enza-induced repression of LNCaP proliferation (Fig. 4biv, v). MiR-197 mimic increased LNCaP/MAR4 proliferation, but did not significantly alter cell growth in presence of Enza (Fig. 4bvi). Representative data is shown and results of replicate experiments are shown in Fig. S7.

### AR-modulatory MiR-361-3p increases migration and invasion of PC cells and induces Snail-independent EMT

**Fig. 5:**
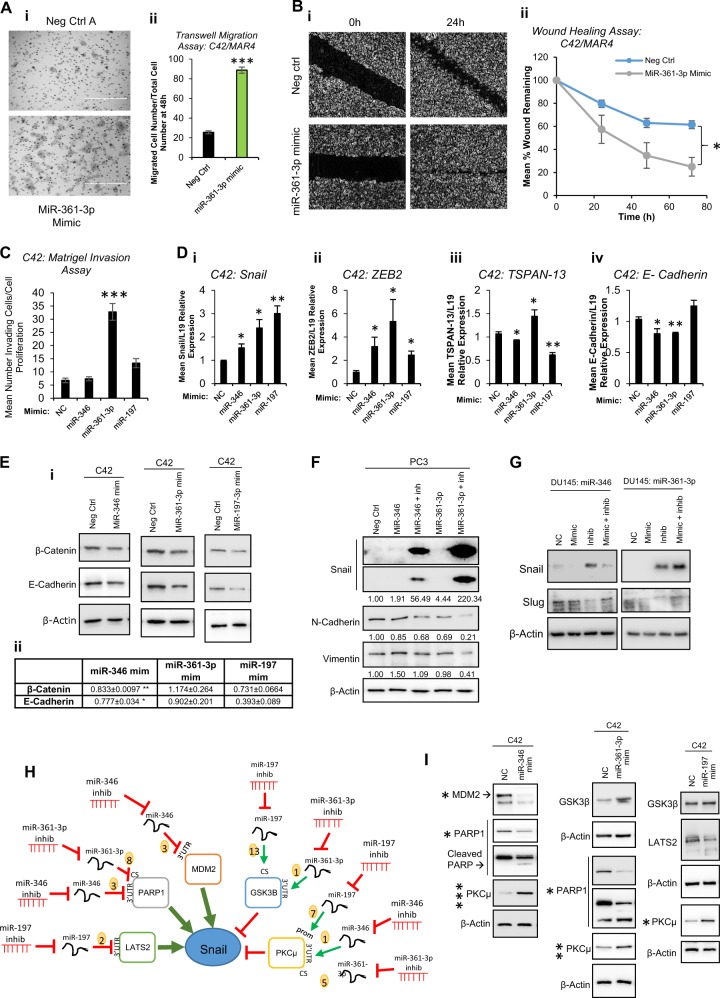
AR-modulatory miRs modulate migration and invasion and induce a unique EMT progression phenotype through modulation of snail-regulatory proteins. **a** Transwell migration assay analysis of C42 cells transfected with miR-361-3p mimic (20 nM) for 48 h prior to seeding onto rat tail collagen-coated transwell membranes for 48 h. Representative image shown in (i), (ii) shows Columns: mean migrated cell number for three independent experiments performed in duplicate normalised to cell number by SRB proliferation assay ± SEM. **b** Wound healing assay analysis of C42 cells transfected with negative control or miR-361-3p mimic (20 nM) for 0–72 h. Representative image shown in (i), in (ii) data are presented as % of day 0 wound remaining and represent mean ± SEM for three independent experiments performed in triplicate (ii). **c** Matrigel invasion assay analysis of C42 cells transfected with miR-346, -361-3p or -197-3p mimic (20 nM) for 48 h prior to seeding onto rat tail collagen- and 5 µg matrigel-coated transwell membranes for 48 h. Columns: mean migrated cell number for three independent experiments performed in duplicate normalised to cell number by SRB proliferation assay ± SEM. **d** qRT-PCR analysis of Snail (i), ZEB2 (ii) TSPAN-13 (iii) and E-Cadherin (iv) mRNA levels in C42 cells transfected with miR-346, -361-3p or -197-3p mimics (20 nM) for 96 h. L19 was used as a normalisation gene. Columns: mean ± SEM for three independent experiments performed in triplicate. **e** Western blot analysis of the epithelial markers β-catenin and E-Cadherin in C42 cells transfected with miR-346, -361-3p or -197-3p mimic (20 nM) for 72 h. β-actin was used as a control for loading. Representative blots of three independent experiments are shown in (i). Images from replicate experiments may be found in Fig. S8E. Densitometry was performed using Image J software and relative protein levels are displayed (ii and Fig. SEii). **f** Western blot analysis of mesenchymal marker proteins Snail, N-Cadherin and Vimentin in PC3 cells transfected with miR-346 or -361-3p mimic (20 nM) ± inhibitor (20 nM) for 96 h. β-actin was used as a control for loading. Densitometry was performed using Image J software and relative protein levels are displayed below bands and in Fig. S8F. **g** Western blot analysis of mesenchymal marker proteins, Snail and Slug, in DU145 cells transfected with miR-346 or -361-3p inhibitor (20 nM) ± mimic (20 nM) for 48 h. β-actin was used as a control for loading. Representative blots of three independent experiments are shown. Images from replicate experiments may be found in Fig. S9. Densitometry was performed using Image J software and relative protein levels are displayed (Fig. S9). **h** MiR-346, -361-3p and -197-3p prostate cancer (PC) AGO-PAR-CLIP targets were enriched for Snail-regulatory proteins. Interactions and direction of modulation by miRs is shown. MiR binding sites from AGO-PAR-CLIP are shown in Fig. S10A. **i** Western blot analysis of Snail-regulatory proteins in C42 cells transfected with miR-346, -361-3p or -197-3p mimic (20 nM) for 96 h. β-actin was used as a control for loading. Representative blots of three independent experiments are shown and *P-*values are indicated by asterix. Images from replicate experiments may be found in Fig. S10b–d. Densitometry was performed using Image J software and relative protein levels are displayed (Fig. S10b–d). *P ≤ 0.05, **P ≤ 0.005, ***P ≤ 0.0001. See also Figs. S8, S9 and S10

Having demonstrated that miR-346, -361-3p and -197 modulate AR activity, proliferation and apoptosis in PC, we hypothesised that these miRs may alter other cancer progression-associated processes. To this end, transwell migration assays were performed in C42 cells transfected with miR-361-3p mimic or negative control mimic. It was demonstrated that miR-361-3p significantly increases migration of PC cells by fourfold (Fig. 5a). In agreement with this, miR-361-3p mimic also significantly enhanced C42 cell migration when assessed by wound healing assay (Fig. 5b). Further, miR-361-3p mimic was shown to increase invasion of C42 cells through matrigel matrix (Fig. 5c), suggesting that miR-361-3p overexpressing cells show increased capacity to actively remodel extracellular matrix, potentially illustrative of increased metastatic potential. Overexpression of neither miR-197 nor miR-346 significantly increased invasion. To investigate whether increased invasive potential is attributable to adoption of a more mesenchymal PC cell phenotype (an initiating event in metastasis), mRNA levels of the established mesenchymal markers, Snail and ZEB2, were assessed in C42 cells post-miR-346, -361-3p or -197 transfection. mRNA levels of both markers were significantly increased by a minimum of 1.5-fold following miR overexpression (Fig. 5di, ii). MiR overexpression also increased expression of additional mesenchymal markers, Slug and Twist-1 at mRNA level (Fig. S8a), and concomitant loss of the epithelial markers, TSPAN-13 and E-Cadherin was observed following miR-346 or -197 overexpression (TSPAN-13) and miR-346 or 361-3p overexpression (E-Cadherin) (Fig. 5diii, iv). Similarly, while miR-346 and -361-1p mimics significantly increased Snail and ZEB2 mRNA levels in LNCaP cells, their inhibition led to reduction of these mesenchymal markers (Fig. S8b, c).

To establish whether such gene expression changes translated to modulation of EMT-associated protein levels, Western blotting was performed to assess epithelial protein levels in epithelial C42 cells and mesenchymal protein levels in more mesenchymal-like PC3 and DU145 cells following transfection of miR mimics and/or inhibitors. It was demonstrated that overexpression of miR-346 and miR-197 significantly reduced β-catenin and E-Cadherin protein levels in C42 cells (Fig. 5e and S8E-iii). In support of enhanced EMT by miR-346 and -361-3p, addition of miR inhibitors in presence of miR mimics reduced protein levels of the mesenchymal markers, N-Cadherin and Vimentin in PC3 cells (Fig. 5f, Fig. S8d). Unexpectedly given increased Snail mRNA levels in presence of miR mimics (Fig. 5di, Fig. S8B), Snail protein levels were markedly induced, by 50-fold and 220-fold, respectively, following miR-346 or -361-3p inhibitor transfection of PC3 cells in presence of the corresponding miR mimic (Fig. 5f, Fig. S8d). Similar significant effects were observed in the presence of miR inhibitors alone (Fig. S8f): while most mesenchymal markers (N-Cadherin, Vimentin and Slug) are lost following miR-346 or -361-3p inhibition, Snail levels are significantly increased. To demonstrate that this was not a cell line-specific effect, the more mesenchymal-like DU145 cells were transfected with miR-346 or -361-3p inhibitor and/or mimic. Again, Snail protein levels were significantly and markedly increased in presence of miR inhibitors, an effect rescued by addition of miR mimic (Fig. 5g, Fig. S9). In contrast, protein levels of closely-related Slug are significantly reduced in the presence of miR inhibitor (Fig. 5g, Fig. S9), consistent with enhanced EMT in presence of miR mimics and reversal of EMT when miRs are inhibited. We hypothesised that this unique EMT protein signature may result from direct regulation of Snail-regulatory proteins by AR-modulatory miRs. Thus we mined AGO-PAR-CLIP data from PC cells [31] for association of miR-346, -361-3p or -197 with 58 unique Snail interactors identified from biogrid.org. 20 of these (although not Snail itself) were found to contain binding sites for at least one of AR-modulatory miR-346, -361-3p or -197 in at least one PC cell line. These include LATS, PARP1, MDM2, GSK3β and PKCµ (Fig. S10a, Fig. 5h). We hypothesised that AR-modulatory miRs may repress positive regulators (green arrows, Fig. 5h) and upregulate negative regulators (red arrows, Fig. 5h) of Snail protein levels in PC cells, with miR inhibitors showing opposing effects. To confirm this hypothesis, Western blotting was performed following transfection of C42 cells with miR-346, -361-3p or 197 mimics. It was demonstrated that, as hypothesised, levels of positive Snail regulators PARP1 and MDM2 were reduced following transfection of miRs for which AGO-PAR-CLIP-identified association in PC cells (Fig. 5i, Fig. S9b, c, d). In addition, increased levels of negative Snail regulators, GSK3β and PKCµ were identified following transfection of AGO-PAR-CLIP-implicated miRs (Fig. 5i, S9b, c, d). These data confirm that AR-modulatory miR-346, -361-3p and -197 can target Snail regulators, potentially contributing to Snail-independent EMT in PC.

### MiR-346, -361-3p and -197 downregulate the AR corepressors, ARHGDIA and TAGLN2, and upregulate the oncogene, YWHAZ, in PC

**Fig. 6:**
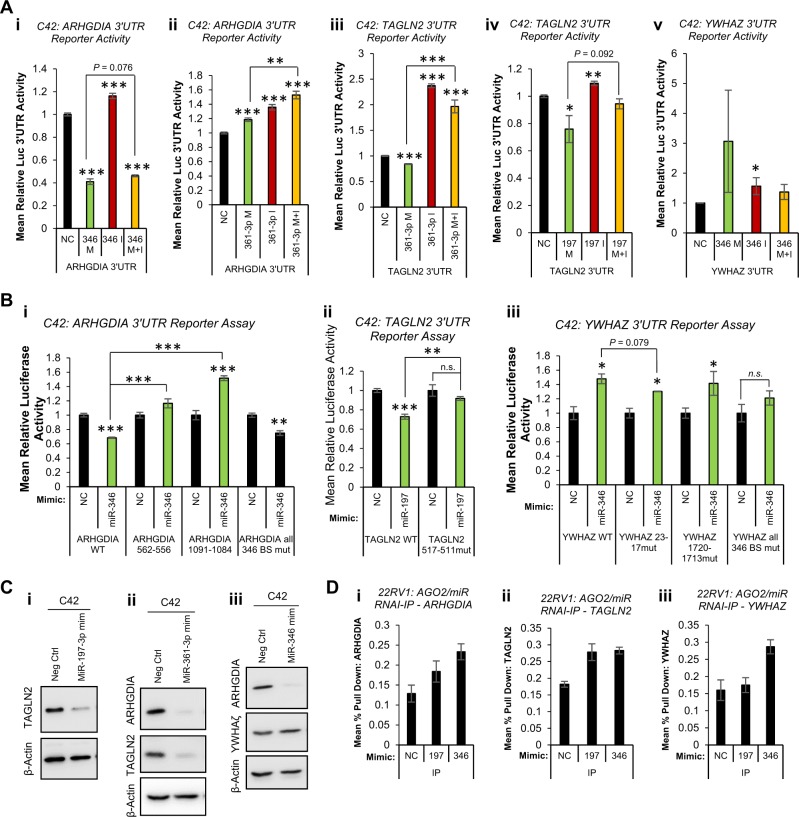
MiR-346, -361-3p and 197-3p repress known tumour suppressors and upregulate the oncogene, YWHAZ in prostate cancer (PC). **a** Luciferase 3′UTR reporter assay analysis of C42 cells transfected with pLightSwitch (i), pLightSwitch-random-3′UTR (ii), pLightSwitch-ARHGDIA-3′UTR (iii), pLightSwitch-TAGLN2-3′UTR (iv) or pLightSwitch-YWHAZ-3′UTR (v) ± miR-346, -361-3p or -197-3p mimics and/or inhibitors (20 nM) for 48 h. **b** Luciferase 3′UTR reporter assay analysis of C42 cells transfected with wild-type or miR binding site-mutant pLightSwitch-ARHGDIA-3′UTR (i), pLightSwitch-TAGLN2-3′UTR (ii) or pLightSwitch-YWHAZ-3′UTR (iii) *±* miR-346, -361-3p or -197-3p mimic, as appropriate, for 48 h. **a**, **b** Luciferase activity was normalised to β-galactosidase activity to correct for transfection efficiency, and data are displayed relative to negative control miR-transfected cells. Columns: mean relative luminescence from three independent experiments performed in duplicate ± SEM. **c** Western blot analysis of TAGLN2, ARHGDIA and YWHAZ protein levels in C42 cells transfected with miR-346, -361-3p or -197 mimic (20 nM) for 96 h. β-actin was used as a control for loading and replicate blots are shown (Fig. S10c). **d** AGO2/biotin-miR RNA-IP analysis of miR-197 and miR-346 association with (i) ARHGDIA, (ii) TAGLN2 and (iii) YWHAZ 3′UTRs. 22RV1 cells were transfected with biotin-labelled miR (200 pmol) for 24 h, followed by two-step immunoprecipitation with AGO2 antibody- and streptavidin-coated beads. RNA was extracted from input and IP samples and qRT-PCR performed for ARHGDIA, TAGLN2 and YWHAZ. Data are presented relative to input values. Columns: mean pulldown relative to input from three independent experiments ± SEM. *P ≤ 0.05, **P ≤ 0.005, ***P ≤ 0.0001. See also Figs. S11, S11 and S13, and Supplementary Tables 3, 4, 5 and 6

We have observed non-AR-mediated effects of miR-346, -361-3p and -197 in PC. Given that pathway analysis revealed additional roles for these miRs in such processes as signal transduction, cell cycle and DNA replication (Fig. S12), we sought to identify additional relevant targets of miR-346, -361-3p and -197. Top AGO-PAR-CLIP targets identified in at least three of five PC cell lines are shown (Tables S4, 5 and 6). ARHGDIA, TAGLN2 and YHWAZ were selected for further analysis based on their interaction with at least one of miR-346, -361-3p or -197 in multiple PC cell lines (Table S7), and also their links to PC progression or AR signalling. The majority of miR binding sites identified were located within the 3′UTR of these genes. ARHGDIA was identified as a target of miR-346 in three PC cell lines, and as a miR-361-3p target in four lines (Table S7). TAGLN2 3′UTR was identified as a miR-361-3p and -197 target in four cell lines. Interestingly, TAGLN2 pseudogene, TAGLN2P1, was also identified as a miR-197 target in two PC lines (Table S7). It is tempting to speculate that TAGLN2 pseudogene could act as a miR sponge to regulate activity of TAGLN2. Finally, miR-346 3′UTR binding sites were identified in YWHAZ 3′UTR in four PC cell lines, while promoters of YWHAZ pseudogenes, YWHAZP2 and YWHAZP7, were found to contain miR-346 binding sites (Table S7). To validate functional association of miRs with identified regions of seed complementarity, C42 cells were transfected with reporter vectors containing ARHGDIA, TAGLN2 or YWHAZ 3′UTRs downstream of Renilla luciferase, ±miR mimics or inhibitors. MiR-346 significantly reduced ARHGDIA 3′UTR activity, while inhibitor alone significantly increased 3′UTR activity by 15% (Fig. 6ai). MiR-346 increased YWHAZ 3′UTR activity, which was partially rescued by addition of miR-346 inhibitor (Fig. 6av), but did not significantly alter activity of empty pLightSwitch, or vector containing random 3′UTR in C42 (Fig. S11Aii) or HEK293T cells (Fig. S11Bi,ii). MiR-361-3p significantly reduced TAGLN2 3′UTR activity in C42 cells, with an opposing increase in 3′UTR activity observed upon inhibitor transfection (Fig. 6aiii), while it enhanced ARHGDIA 3′UTR activity (Fig. 6aii). This may be due to off-target effects on the vector, as miR-361-3p increased luciferase activity both of empty pLightSwitch and vector containing a random 3′UTR sequence (Fig. S11A). Finally, miR-197 mimic significantly reduced 3′UTR activity of TAGLN2, which was significantly opposed through addition of miR-197 inhibitor (Fig. 6aiv). Similar results were obtained upon repetition of experiments in HEK293T cells (Fig. S11biii-iv).

To confirm miR targeting of 3′UTRs through association with AGO-PAR-CLIP-identified seed region binding sites, key residues within such seed binding sites were mutated (minimum of three residues within seed-complementary region) and 3′UTR luciferase reporter assays performed ± miR mimics. AGO-PAR-CLIP-identified miR-346 seed binding sites at nucleotides 556–562 and 1084–1091 of the ARHGDIA 3′UTR. The more 5’ site shows greater conservation across mammals than the 3′ site. Mutation of each site individually, and of both sites in the same vector, was performed. Mutation of either region was able to completely abolish miR-346 induced loss of ARHGDIA 3′UTR activity. Interestingly, although the extent of miR-346-induced loss of 3′UTR activity was reduced following mutation of both sites, complete rescue of repression was not observed (as seen for individual sites) (Fig. 6bi). One poorly conserved miR-197 binding site was identified by AGO-PAR-CLIP at nucleotides 511–517 of TAGLN2 3′UTR. Mutation of this seed-complementary region significantly abrogated miR-197-induced loss of TAGLN2 3′UTR activity (Fig. 6bii). Finally, two miR-346 binding sites were identified by AGO-PAR-CLIP at nucleotides 17–23 and 1713–1720 of YWHAZ 3′UTR, with the proximal site very highly conserved across mammals, and the more distal site relatively poorly conserved. Mutation of these seed-complementary regions individually only minimally reduced miR-346-induced increase in YWHAZ 3′UTR activity, although this effect was greater for the proximal site (Fig. 6biii). However, mutation of both sites significantly abrogated miR-346-mediated increase in YWHAZ 3′UTR activity (Fig. 6biii).

To investigate whether altered miR target 3′UTR activity upon miR transfection is translated into altered protein levels, Western blots were performed in C42 cells following transfection of miR-197, -361-3p or -346. It was shown that miR-197 significantly reduced levels of its putative target, TAGLN2, by 56% (Fig. 6ci, Fig. S11Ciii, iv). Similarly, miR-361-3p significantly decreased protein levels of TAGLN2 by 70%, and ARHGDIA by 59% (Fig. 6cii, Fig. S11Cii, iv). In addition, miR-346 significantly reduced ARHGDIA protein levels by 74% but increased YHWAZ protein by 1.2-fold (Fig. 6ciii, Fig. S11Ci, iv). Finally, to confirm physiological association of miRs with target transcripts, we performed AGO/biotin-miR RNA-IP. It was found that levels of ARHGDIA transcript are significantly enriched in 22RV1 cells following IP with miR-346 as compared to miR-197 or non-targeting control (Fig. 6di). Although pulldown of TAGLN2 was greater in presence of miR-197 compared to non-targeting control, there was no significant enrichment when compared to miR-346, which was not identified as targeting TAGNL2 by AGO-PAR-CLIP in 22RV1 cells (Fig. 6dii). In contrast, enrichment of YWHAZ was increased twofold upon miR-346 transfection, compared to non-targeting control or miR-197 (Fig. 6diii). Taken together, these data confirm functional repressive targeting of ARHGDIA by miR-346 and -361-3p and TAGLN2 by miR-361-3p and -197, and upregulatory targeting of the PC oncogene, YWHAZ, by miR-346.

Additional putative miR-346 targets (IL18, LIF, BTK and TAP1), miR-361-3p targets (ATXN7L3, MAPT and PRMT2) and -197 targets (ACVR1, TSPAN3, TUSC2), as identified from miRTarBase, were validated in PC cells through a combination of AGO/biotin-miR RNA-IP, qPCR and western blotting (Fig. S13).

### siRNA-mediated silencing of ARHGDIA, TAGLN2 and YWHAZ phenocopies effects of MiRs (or MiR inhibition) in PC

**Fig. 7:**
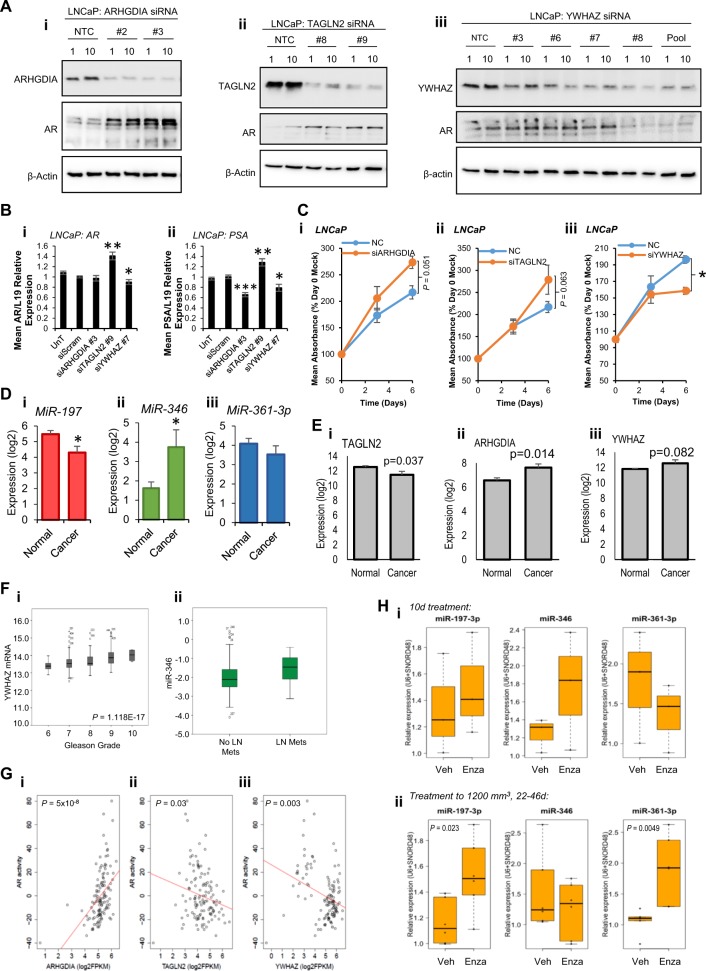
AR-modulatory miR target siRNA silencing phenocopies miRs. **a** Western blot analysis of ARHGDIA, TAGLN2, YWHAZ and AR protein levels in LNCaP cells transfected with siRNAs (1 or 10 nM) targeting (i) YWHAZ, (ii) TAGLN2 or (iii) ARHGDIA for 72 h. A minimum of two different siRNAs were used for each gene. β-actin was used as a control for loading. **b** qRT-PCR analysis of (i) AR or (ii) PSA mRNA levels in LNCaP cells transfected with ARHGDIA, TAGLN2 or YWHAZ siRNAs (10 nM) for 48 h. L19 was used as a normalisation gene. Columns: mean ± SEM for three independent experiments performed in triplicate. **c** SRB proliferation assay analysis of LNCaP cells transfected with siRNA targeting ARHGDIA, TAGLN2 or YWHAZ (10 nM) for 6 days. Data are presented relative to absorbance at day 0. Points: mean absorbance at 492 nm for three independent experiments performed in quadruplicate ± SEM. **d**, **e** MiR-197 (**d**i), miR-346 (**d**ii), miR-361-3p levels (**d**iii), TAGLN2 (**e**i), ARHGDIA (**e**ii) and YWHAZ (**e**iii) RNA levels in fresh-frozen tissue from five PCs and six normal prostates (GSE34932). **f** YWHAZ mRNA levels in PC tumours of Gleason Grades 6–10 (i) and miR-346 levels in PC tumours of lymph node-positive and –negative patients from the TCGA-PRAD data set of 333 primary prostate cancers (PCs). **g** Linear regression analysis of correlation between AR activity and ARHGDIA (i), TAGLN2 (ii) and YWHAZ (iii) levels in CRPC patient tumours (*n* = 122, transcriptome data from SU2C-PCF). AR activity score is an accumulation measurement of AR pathway activity based on 43 genes whose expression level is regulated by AR in PC cell lines [56] and metastatic PC [57]. **h** qRT-PCR analysis of miR-197, miR-346 and -361-3p levels in patient-derived xenografts implanted into nude mice and derived from a single biopsy from an AR-amplified, C-MYC-gain CRPC patient. Enzalutamide (10 mg/kg) or vehicle treatment was initiated when xenografts reached 300 mm^3^, and mice killed at day 10 (i) or when tumours reached 100 mm^3^ (ii) (mean treatment duration = 35.5 days). (i) *n* = 3 per treatment group, (ii) *n* = 6 per treatment group. Data were normalised to U6 and SNORD48 levels. *P ≤ 0.05, **P ≤ 0.005, ***P ≤ 0.0001. See also Figs. S14, S15, S16

If ARHGDIA, TAGLN2 and YWHAZ represent physiologically relevant miR-346, -361-3p and -197 targets in PC, we reasoned that siRNA-mediated silencing of these genes should phenocopy effects of miR overexpression (or miR inhibition in the case of YHWAZ, since miR-346 increases YHWAZ 3′UTR activity and protein levels). To address this, we first examined AR protein levels in LNCaP cells transfected with several single siRNAs targeting ARHGDIA, TAGLN2 or YWHAZ. It was found that both ARHGDIA siRNAs tested reduced ARHGDIA protein levels by up to 80%, with AR protein levels concomitantly increased by up to 4.5-fold (Fig. 7ai, Fig. S14a). Similarly, TAGLN2 siRNAs reduced TAGLN2 protein levels by up to 90% and increased AR protein levels up to 10.5-fold (Fig. 7aii, Fig. S14b). Interestingly, YWHAZ siRNAs reduced YWHAZ protein levels by up to 70%, but contrasting effects were observed on AR protein: where siRNAs efficiently reduced YWHAZ protein levels (for example, siRNA #8 and pooled siRNAs), AR protein levels were reduced by up to 60% (Fig. 7aiii, Fig. S14c), while less efficient silencing of YWHAZ (for example, by siRNAs #3 and #6) led to a small increase in AR protein levels of up to 20%, suggestive of threshold effects of YWHAZ on AR protein levels. These data demonstrate that siRNA-mediated silencing of ARHGDIA and TAGLN2 phenocopies miR-346, -361-3p and -197-induced increase in AR protein levels.

In support of the above data, siRNA silencing of TAGLN2 significantly increased AR mRNA levels and expression of the AR target gene, PSA, similar to effects observed for the TAGLN2-targeting miRs 361-3p and 197 (Fig. 7b). As anticipated, siYWHAZ phencopied effects of miR-346 inhibition, resulting in significant reduction of both AR and PSA mRNA levels (Fig. 7b). siRNA silencing of ARHGDIA did not significantly modulate AR mRNA levels, but unexpectedly and significantly reduced PSA levels in LNCaP cells (Fig. 7b). To ascertain whether siRNA silencing of miR targets can replicate phenotypic effects of miR manipulation, SRB assays were performed in LNCaP cells following transfection of ARHGDIA, TAGLN2 or YWHAZ siRNAs. It was demonstrated that knockdown of ARHGDIA increased cell number by 1.3-fold (Fig. 7ci) and that TAGLN2 siRNA enhanced proliferation by 1.25-fold (Fig. 7cii). In contrast, knockdown of YWHAZ significantly reduced LNCaP cell proliferation by 20% (Fig. 7ciii). Taken together, these data demonstrate that targeting of ARHGDIA, TAGLN2, YWHAZ are likely to significantly contribute to oncogenic miR-346, -361-3p and -197-induced phenotypes.

To examine the relevance of AR-modulatory miR expression and levels of their targets genes to clinical PC development and progression, bioinformatic analyses of publically available expression data sets were performed. Upon examination of AR-modulatory miR levels in fresh-frozen tissue from five PCs and six normal prostates (GSE34932), it was found that miR-197 was significantly decreased in cancer vs normal, while miR-361-3p is unchanged and miR-346 significantly increased (Fig. 7d). TAGLN2 mRNA levels were significantly lower in cancer vs normal and ARHGDIA and YWHAZ levels elevated significantly and non-significantly, respectively, in the same data set (Fig. 7e). In analysis of TCGA-PRAD data from 333 primary PCs, increased YWHAZ transcript correlated with Gleason grade in a highly-significant manner (Fig. 7fi), and was higher in patients with lymph node metastases, biochemical recurrence and in non-organ-confined disease (Fig. S15). No such relationships were identified for TAGLN2 or ARHGDIA. In the same data set, miR-346, but not -361-3p nor -197, was significantly elevated in lymph node-positive disease (Fig. 7fii). As anticipated, ARHGDIA expression inversely correlated with its targeting miRs, miR-346 and miR-361-3p, in the TCGA-PRAD data set (Fig. S16a, b). However, miR-361-3p was found to positively correlate with its target, TAGLN2 (Fig. S16c), and miR-346 negatively correlated with its positively regulated target, YWHAZ (Fig. S16d). When linear regression analysis was conducted to assess correlation between AR activity score and miR targets in CRPC patient tumours (*n* = 122) from SU2C [32], a significant positive correlation of ARHGDIA expression with AR activity was found (*P* *=* 5 × 10^−8^) (Fig. 7gi), while TAGLN2 is significantly negatively correlated (*P* *=* 0.03) (Fig. 7gii). Similarly, YHWAZ is negatively associated with AR activity, although samples separate into two populations according to YWHAZ expression, and both correlate positively with AR activity when assessed individually (Fig. 7giii). In the same data set, patients with high levels of miR-197 showed a near-significant (P = 0.0501) decreased survival compared to miR-197 low patients (Fig. S16e). Neither miR-346 (Fig. S16f) nor miR-361-3p (alone, Fig. S16g, or combined with miR-197, not shown) correlated with survival in TCGA-PRAD. Of note, MiR-361-3p significantly correlated with AR activity in this cohort (Fig. S16h), albeit with an *r*^2^ value of 0.13.

We next investigated the effect of treatment on AR-modulatory miRs in patient-derived models. CP50 PDX was derived from a CRPC patient biopsy with chromosome 8 gain and AR amplification who had progressed through all standard of care therapies (Welti et al; submitted to Clinical Cancer Research). Short-term (10 days) treatment with enzalutamide (10 mg/kg; *n* = 3) did not significantly alter miR levels compared to vehicle treatment (Keptose HPB; *n* = 3) (Fig. 7Hi). However, long-term treatment (mean 35.5 days) with enzalutamide (*n* = 6) resulted in significant increases in miR-197 and -361-3p levels as compared to vehicle (*n* = 6) (Fig. 7hii). MIR-346 levels were not altered (Fig. 7hii).

Taken together, these data confirm that AR-modulatory miRs and their biologically relevant PC targets are altered in cancer vs normal tissue, correlate with disease progression and AR activity, and are altered in response to hormone therapy in a CRPC patient tumour.

## Discussion

In PC that is refractory to conventional LBD-targeting anti-androgens, AR-targeting miRs may represent promising therapeutics or drug targets. A handful of studies have demonstrated direct AR targeting by miRs, however with little consensus. Much remains to be learnt regarding the roles of miRs and the AR 3′UTR in disease progression, the ability of miRs to integrate AR signalling with other pathways, and relationships between AR and other targets of AR 3′UTR-associating miRs. In an effort to increase consensus on AR-modulatory miRs, we performed a miR inhibitor library screen in LNCaP and C42 PCa cells stably expressing a luciferase-based AR activity reporter.

Although little agreement was observed between top hits from our screen and that of Kumar et al. [13], which used a miR mimic library, 22 miR inhibitors that increased AR activity above our stringent threshold in LNCaP and/or C42 cells agreed with their screen in that the corresponding miR mimic significantly reduced LNCaP cell viability, and decreased PSA and/or AR protein levels/ activity in at least two of three PC cell lines tested (LNCaP, VCaP, LAPC4). Four additional miRs, inhibitors of which decreased AR activity in our screen, were likewise identified by these criteria in the Kumar study. Of thirteen miRs found by Östling et al. to significantly reduce AR 3′-UTR activity, although corresponding inhibitors did not found to modulate AR activity above our threshold, there was some agreement for seven, as we found that miR-421 and -449a increased AR activity in C42 cells; miR-135b, -634 and -654-5p increased AR activity in LNCaP; while miR-34c and miR-9 increased AR activity in both lines. We were unable to validate previously reported reduction of AR levels, in various cell lines, by miR-185 [16], miR-34a [33], or miR-488-5p [13, 19]. We identified agreement with our screening data (LNCaP and/or C42) for 26 of 64 miRs identified to modulate PSA protein levels in LNCaP and LAPC4 cells in a miR mimic screen by Larne et al. [34], although only three had B Score > 6 in our screen. There are many likely reasons for lack of agreement between studies: differences in library types (mimic or inhibitor), inhibitor/mimic concentrations, cell lines with different transfection efficiencies and endogenous miR levels, end-point assays, and statistical scores and thresholds for analysis.

Upon validation of screen hits, we demonstrated that miR-346, -361-3p and -197 inhibitors markedly reduced AR activity in both LNCaP and C42 cells, with opposite effects observed for corresponding miR mimics. In corroboration we also showed that the inhibitors significantly reduced AR mRNA and protein levels, and target gene expression, effects rescued by addition of miR mimics. Since these effects were observed after only 24 h, it is likely that these miRs directly regulate AR through activatory association with the 3′UTR, in contrast to the classical model of target repression by miRs. In support of this, we identified binding sites for all three miRs in the extended 6.9 kb AR 3′UTR, which has been described in PC cells by a number of studies [15, 27], but is not used by miR binding prediction tools, which use the UCSC genome browser and Genbank-defined 436 nt AR 3′UTR sequence (NM_000044). This could potentially result in exclusion of critical but uncharacterised regulators of AR activity from previous investigations. Direct miR-346, -361-3p and -197 upregulation of AR 3′UTR activity was confirmed through use of AR 3′UTR reporters in combination with miR mimics, which significantly increased AR 3′UTR activity, with specificity demonstrated through additional transfection of miR inhibitors and miR seed binding site mutagenesis. Such miR-mediated AR upregulation could provide a mechanism for maintenance of AR activity in castrate conditions. Further, miR-346 and -197 prevented ActD-induced loss of AR transcript, so may additionally repress AR mRNA turnover. This study is not the first to report miR-346 upregulation of target genes: miR-346 increases AGO2 mRNA and protein levels in cervical, gastric and colorectal cancer cell lines through formation of a miR-346 mid-region ‘bulge’ upon association with AGO2 3′UTR, which in turn increases recruitment of GRSF1 [35]. The same authors showed that a similar mechanism increases recruitment of miR-346-bound hTERT mRNA to ribosomes to enhance translation in a GRSF1-dependent manner [36]. It will be interesting to assess whether miR-346:AR 3′UTR association results in similar miR looping and recruitment of RNA binding proteins.

The identification of an extended AR 3′-UTR in PCa cell lines is interesting in the context of a proposed novel mechanism for altered gene regulation, whereby dynamic 3′UTR shortening prevents miR targeting. In cancer cells, mRNAs with shortened 3′-UTRs produced tenfold more protein, in part due to loss of miR-mediated repression [37]. While it has been proposed that active AR 3′-UTR shortening represents a further mechanism of PCa progression [38], permitting AR to evade regulation by tumour-suppressive miRs, such miR-34a and miR-34c [15], there is currently no evidence for reduced miR binding and consequently higher AR protein levels following AR 3′-UTR shortening. Further studies are required to assess its potential role in cancer progression, especially given our data showing AR 3′UTR as a region positively regulated by oncogenic miRs.

Expression of constitutively active AR variants lacking the LBD represent a potential mechanism of cell survival in CRPC. AR-v7 has a distinct 3′UTR sequence from WT-AR, while ARv567^es^ 3′UTR has considerable overlap with WT-AR 3′UTR but is extended at the 3′ end [39]. We therefore hypothesised that ARv567es would be subject to regulation through the same miR-346 and -361-3p binding sites as WT-AR, while AR-v7 would not. Indeed, we observed decreased ARv567^es^ mRNA and protein levels following transfection of miR-346 and miR-361-3p inhibitors in 22RV1 and CWR-R1-D567^es^ cells. AR-v7 mRNA was significantly decreased following miR-361-3p inhibitor transfection in 22RV1 and C42 cells, possibly due to an AR-v7/9/4-unique miR-361-3p 7mer site between 981-987nt of AR-v7 3′UTR (Table S3). AR-v7 does not contain any predicted miR-346 binding sites however, and the unexpected decrease in its expression in both 22RV1 and C42 cells following miR-346 inhibitor transfection (Fig. S4c) may be attributable to feedback regulation from loss of WT-AR. Protein levels of AR variants of size consistent with AR-v1, -v4 and -v7 were markedly downregulated upon miR-361-3p inhibitor treatment in 22RV1 and CWR-R1-AD1 cells. Together, these data indicate that modulation of miR-346 and miR-361-3p can alter levels of constitutively active AR variants as well as WT/full-length AR.

Given their effects on AR activity, we examined phenotypic consequences using proliferation assays and found that miR-346 and -361-3p inhibitors reduce proliferation, supporting a tumour-promoting/oncogenic effect of the corresponding miRs. In AR-positive cells the inhibitors showed additive effects with AR silencing and Enzalutamide treatment, conversely, mimics increased PC cell proliferation and abrogated AR siRNA- or Enza-induced loss of cell proliferation. This extended to AR-variant-driven lines, and the enhanced effects of miR-346 inhibition on AR-variant-driven vs parental PC cell growth likely reflect the presence of an additional variant-specific miR-346 seed complementarity site in v567es 3′UTR. However, miRs have potentially dozens of target mRNAs, and the phenotypic consequences of miR inhibition are likely to be both AR-mediated and non-AR-mediated Indeed, miR-346, -361-3p and -197 inhibitors were able significantly reduce growth of both AR-positive and AR-negative cells in a dose-dependent manner. Looking into potential modes of action by which miR-346, -361-3p and -197 inhibitors reduce proliferation, we found that all three significantly increased apoptosis in LNCaP cells, and enhanced cell death induced by AR inhibition//ablation, while the mimics mostly abrogated this. The observation that, contrary to expectation, miR-346 mimic showed similar effects to inhibitor in increasing Enza-induced apoptosis may be attributable to very large (280,000-fold) upregulation of miR-346 upon transfection of mimic at 20 nM final concentration (Fig. S1Ai), which may generate toxicity. Indeed, 10 nM miR-346 mimic was shown to repress C42 and LNCaP proliferation, and cells showed signs of toxicity at this concentration (Fig. S6b). All three inhibitors showed additive effects with AR siRNA on AR target gene expression, while corresponding mimics rescued AR siRNA-mediated loss of AR, PSA and KLK2 expression. However, the observation that they could not completely rescue effects of AR siRNA on proliferation, apoptosis and AR target gene expression supports that these miRs also function, in part, through non-AR pathways. This is reinforced by the observation that miR-346 and -361-3p inhibition significantly reduces proliferation of AR-negative DU145 cells. Together, these data suggest that miR-346, miR-361-3p and miR-197 can act as oncomiRs to increase PC proliferation and inhibit apoptosis, partly, but not wholly, through regulation of AR signalling.

As further evidence of its oncomiR function, miR-361-3p significantly increases migration and invasion of C42 PC cells. It is thus interesting that significant downregulation of miR-361-3p was observed in prostatic secretions of 23 (low grade) PC patients compared to 25 BPH controls [40]. It would be interesting to know whether observed miR dysregulation was also identified in tissue, urine or plasma, and whether the correlation is retained in men with higher-grade tumours. Conceivably, these data may suggest active retention of oncomiR-361-3p within PC cells, to drive EMT and migration/invasion. Indeed, miR-361-3p, -346 and -197 all reduced levels of epithelial markers in C42 cells (which have epithelial morphology), while miR-346 and -361-3p inhibitors decreased protein levels of mesenchymal markers in the more aggressive PC3 and DU145 cells. The exception was Snail, a mesenchymal marker, which was markedly increased. We hypothesised that miR-346, -361-3p and -197 modify levels of Snail-regulatory proteins, and analysis of AGO-PAR-CLIP data confirmed these to be enriched in their targets: Snail repressors GSK3β and PKCµ appear to contain activatory miR binding sites (levels are increased in presence of targeting miRs), while Snail activators MDM2, PARP1 and LATS2 contain repressive miR binding sites. An alternative, possibly complementary, explanation is that the miRs induce intermediate EMT ‘plasticity’ that promotes migration and invasion in a Snail-independent manner. Evidence for such Snail-independent EMT has been suggested by a number of studies: p38 MAP kinase and p38 interacting protein (p38IP) are required for E-Cadherin downregulation during EMT in mouse embryo gastrulation, in a Snail-independent manner [41]; Snail and Slug induce distinct EMT programmes in mammary stem cells as compared to tumour initiating cells [42]; and high levels of the E-Cadherin repressors, ZEB1 and ZEB2, which could initiate EMT independently of Snail, were found in recurrent oral squamous cell cancer as compared to primary tumours, suggestive of non-canonical EMT progression in advanced cancer [43].

To identify additional PC-relevant targets of these miRs that mediate the non-AR-mediated effects, we exploited AGO-PAR-CLIP to identify miR:target association in a panel of PC cell lines. We focussed on genes previously implicated in PC and with multiple 3′UTR binding sites for more than one of miR-346, 361-3p and -197, selecting YWHAZ, ARHGDIA and TAGLN2 for further analysis. Our data confirm ARHGDIA and TAGLN2 as bona fide AR-modulatory miR targets, downregulated by miR-346 and -361-3p, and miR-361-3p and -197, respectively. ARHGDIA and TAGLN2 have both previously been shown to repress AR transcriptional activity, and to have tumour-suppressive roles [44–47]; their downregulation has clear implications for PC development, and further supports oncogenic activity of miR-346, 361-3p and -197. Conversely, the androgen-regulated PC oncogene YWHAZ was upregulated by miR-346, likely through association between the miR and nucleotides 17–23 of YWHAZ 3′UTR. YWHAZ has been shown to directly associate with AR, to promote proliferation, migration, survival and resistance to apoptosis in PC cells [48], to increase in PC patient tumours as compared to benign tissue, and to positively correlate with presence of metastases in clinical tumour samples [49]. Thus, miR-346-mediated YWHAZ upregulation could enhance PC progression.

To confirm biological relevance of miR targeting of ARHGDIA, TAGLN2 and YWHAZ, we examined the ability of siRNAs against these genes to phenocopy effects of miR-346, -361-3p and -197 in PC cells. Silencing ARHGDIA or TAGLN2 resulted in increased AR protein levels, target gene expression and PC cell growth, similarly to miR-346, -361-3p or -197 mimics. Conversely, and as anticipated, siYWHAZ phenocopied miR-346 inhibition reducing AR protein levels and represseing PC cell growth. We hypothesise that AR-modulatory miRs may have important functions at different stages of PC: miR-346 levels are higher in cancerous vs normal prostate tissue and elevated in tumours of patients with lymph node metastases compared to those without, while not altered in Enza-treated CRPC patient xenografts. In contrast, miR-197 is reduced and -361-3p unchanged in cancerous vs normal prostate, but both were significantly elevated in Enza-treated CRPC xenografts, although only after long-term treatment. This suggests that while miR-346 may contribute to progression of early-stage PC (through upregulation of YWHAZ and AR activity), miR-197 and -361-3p may act at the castrate-resistant stage of the disease to promote or maintain resistance to AR-targetting therapies, through downregulation of tumour-suppressive targets and stabilisation of AR transcript.

Taken together, these data identify miR-346, miR-361-3p and miR-197 as AR-modulatory miRs in PC that associate with an extended form of the AR 3′UTR to increase transcript stability and AR activity. AR-modulatory miRs modulate apoptosis, proliferation, EMT, migration and invasion of PC cells, and their inhibition shows additive effects with anti-androgens, highlighting the potential for combination approaches for the treatment of PC. Silencing PC-relevant miR targets phenocopies effects of these miRs, supporting their physiological relevance.

## Materials and methods

### Mammalian cell culture

Cells were maintained at 37 °C in 5% CO_2_. PC3, 22RV1, LNCaP, C42, DU145 and HEK293T cells were maintained and passaged in RPMI-1640 or Dulbecco’s Modified Eagle’s Medium (DMEM – HEK293T only) (Sigma) supplemented with 10% fetal bovine serum (FBS), 100 U/ml penicillin, 100 µg/ml streptomycin and 2mM l-glutamine (Sigma). LNCaP/ARE, C42/ARE and DU145/ARE cells were maintained and passaged as above but with 10% tetracycline-free FBS, 12 µg/ml blasticidin and 500 µg/ml G418. LNCaP/ARsiRNA cells (a kind gift of Paul Rennie, Vancouver Prostate Centre, Canada) were maintained as above, but with 2.4 µg/ml blasticidin and 1 µg/ml puromycin. Parental 22RV1 cells and AR WT-knock out derivatives, and parental CWR-R1-AD1 cells and CWR-R1-D567^es^ derivative lacking WT-AR but expressing AR-v567^es^ were kind gifts from Luke Gaughan, Newcastle University, UK, and Scott Dehm, University of Minnesota, USA, respectively [50]. Cell lines were routinely tested for Mycoplasma contamination on a monthly basis

### Plasmid stocks

pMiR-Report-AR 6.9 kb 3′UTR reporter plasmids 1–7, representing seven different regions of the 6.9 kb AR 3′UTR (Fig. 2d) were a kind gift from Dr Shawn Lupold(36). pLightSwitch-Empty, pLightSwitch-Random#2, pLightSwitch-ARHGDIA 3′UTR, pLightSwitch-TAGLN2 3′UTR and pLightSwitch-YWHAZ 3′UTR were purchased from OriGene. Putative miR-346, -361-3p and -197-3p binding sites were mutated in the above vectors by site-directed mutagenesis using the QuikChange Lightning Site-Directed Mutagenesis Kit (Stratagene).

### High-throughput LNA microRNA inhibitor screening

C42/ARE and LNCaP/ARE cells were seeded into thirteen 96-well, white-walled, flat-bottomed cell culture plates per cell line in hormone-depleted, antibiotic-free RPMI medium 48 h prior to transfection. Seeding densities were 3000 cells per well (C42/ARE) and 4000 cells per well (LNCaP/ARE). 8 h prior to transfection, cells were supplemented with 0.1 nM Mibolerone. Cells were transfected with LNA MicroRNA Inhibitor Library (Exiqon) to a final concentration of 20 nM using Lipofectamine RNAiMax for 48 h. Controls were AllStars Hs Cell Death Control siRNA (Qiagen), AllStars Negative Control siRNA (Qiagen) and LNA Negative Control A (Exiqon).

### MicroRNA inhibitor/mimic, siRNA transfection

MiRCURY LNA microRNA inhibitors and mimics (Exiqon) and/or miRidian microRNA mimics (GE healthcare) were transfected into cells at final concentration of 0–30 nM in antibiotic-free conditions using Lipofectamine RNAiMax as per the manufacturer’s recommendations. siRNAs (Flexitube, Qiagen) were transfected as above, final concentration 10 nM. Co-transfection of plasmids and siRNAs or microRNA inhbitors/mimics was performed using JetPrime transfection reagent (Polyplus) according to the manufacturer’s protocol.

### RNA extraction

RNA was extracted using Trizol according to the manufacturer’s specifications. For RNA extraction from PDX tissue, samples were subjected to homogenisation using Precellys24 Homogeniser in the presence of Trizol.

### MiR and mRNA quantitative real-time PCR

Mature miR expression was quantified by quantitative real-time RT-PCR using TaqMan microRNA assays and TaqMan Universal PCR Master Mix (Applied Biosystems) according to the manufacturer’s protocol. For detection of mRNAs, cDNA was prepared from 500 ng total RNA using Precision qScript Reverse Transcription kit (PrimerDesign) and oligo d(T) primers. cDNAs were amplified using 2× Fast SYBR Green Master Mix (Applied Biosystems) and 250 nM forward and reverse primers (Table S1). All data were analysed using the ΔΔC_t_ method, with U18 and L19 as endogenous references for miR and mRNA levels respectively, using DMSO/ethanol-treated samples as calibrators where appropriate.

### 3′UTR reporter assays

HEK293T or C42 cells seeded in 24-well plates were co-transfected with 400 ng pMiR-Report AR 6.9 kb 3′UTR #1–7, pLightSwitch-Empty, pLightSwitch-Random#2, pLightSwitch-ARHGDIA 3′UTR, pLightSwitch-TAGLN2 3′UTR, or pLightSwitch-YWHAZ 3′UTR (Origene) alongside 50 ng pdm-LacZ (β-galactosidase expression vector) and 0–20 nM final concentration non-targeting, miR-346, miR-361-3p or miR-197 mimic and/or inhibitors (see above) using JetPrime Transfection reagent for 48 h. For AR 3′UTR reporter assays, luciferase assays were performed using Luclite assay as below. For pLightSwitch plasmids, LightSwitch luciferase assay (Active Motif) was used according to the manufacturer’s protocol. For both assays, luciferase activity was normalised for transfection efficiency using the Galacton kit (Tropix) as previously described [51].

### Luciferase assays

Luciferase assays were performed using the Luclite assay (Packard, USA). Cell medium was aspirated from wells and 30 µl (96-well plates) or 60 µl (24-well plates) of Reporter Lysis Buffer (Promega) added per well. Plates were stored at −80 °C for a minimum of 15 min. Plates were thawed and 30 µl luciferin substrate added per well (96-well plates) or 20ul of cell lysate dispensed in duplicate into white-walled 96-well plates and 20ul luciferin substrate added per well (24-well plates). Plates were incubated for 15 min in the dark prior to luminescence reading using the GloMax system (Promega) or Victor luminescence counter. Luciferase activity was normalised for cell number by Bradford assay for protein content, where appropriate.

### Actinomycin D transcriptional inhibition experiments

C42 cells in 6-well plates previously transfected with miR mimics for 24 h were treated with Actinomycin D (1 µg/ml) for 4 h prior to harvesting and RNA extraction.

### Cell Lysis, western blotting and antibodies

Cells were lysed and protein extracted as described [26]. Proteins were resolved by 8–12% SDS-polyacrylamide gel electrophoresis and electroblotted to nitrocellulose membrane (Bio-Rad). After blocking (5% non-fat dried milk powder in 0.05% Tween-20 in 1x TBS) for 40 min, membrane was incubated with mouse anti-androgen receptor mAb (ThermoFisher, MA513426), rabbit anti-beta-catenin pAb (Cell Signalling Technology, 8480), rabbit anti-E-Cadherin pAb (Cell Signalling Technology, 3195), rabbit anti-Snail pAb (Cell Signalling Technology, 3879), rabbit anti-N-Cadherin pAb (Cell Signalling Technology, 13116), rabbit anti-Vimentin pAb (Cell Signalling Technology, 5741), rabbit anti-Slug pAb (Cell Signalling Technology, 9585), rabbit anti-MDM2 mAb (Abcam, ab178938), mouse anti-PARP1 mAb (Santa Cruz Biotechnology, sc-8007), rabbit anti-PKCµ mAb (Abcam, ab108963), mouse anti-GSK3β mAb (Abcam, ab93926), rabbit anti-LATS2 pAb (Abcam, ab70565), rabbit anti-ARHGDIA pAb (Santa Cruz Biotechnology, sc-360), mouse anti-TAGLN2 mAb (Santa Cruz Biotechnology, sc-373928), rabbit anti-YWHAζ pAb (Abcam, ab51129), mouse anti-BTK mAb (R + D Systems, MAB5807), mouse anti-LIF mAb (Abcam, ab34427), mouse anti-β-tubulin mAb (Sigma, T4126), or mouse anti-β-actin mAb (Abcam, ab6276) for 1 h and visualised using goat anti-mouse, goat anti-rabbit, goat anti-rat or rat anti-goat IgG-HRP as appropriate. Detection was by Luminata Forte HRP substrate (Millipore).

### Sulphorhodamine B (SRB) Assay

Cell number was assayed using the SRB assay as previously described [52].

### Luminescent caspase 3/7 activation assay

Caspase assays were performed using the Caspase-Glo 3/7 kit (Promega) according to the manufacturer’s instructions, 48 h post-transfection with miR mimics or inhibitors. Luminescent signal was detected using a Victor luminometer.

### Wound healing assay

Cells seeded in 6-well plates were transfected with miR mimics as described. 48 h post-transfection, an artificial ‘wound’ was created in the cell monolayer using a 200 µl pipette. Medium was replaced with antibiotic-free, phenol red-free RPMI supplemented with 5% sFCS to minimise proliferation. Wounds were imaged at 0 and 24 h and wound area quantified using Image J.

### Transwell migration/matrigel invasion assays

Cells seeded in 6-well plates were transfected with miR mimics as described. 48 h post-transfection, outer surfaces of 8 µm pore transwell inserts were pre-coated with 2.5 μg rat tail collagen type I in cold, non-supplemented, phenol red-free, RPMI and air-dried for 2 h and placed in 24-well companion plates. Transfected cells were harvested and seeded (3 × 10^4^ cells per condition) onto collagen-coated transwell membranes in 100 μl serum-free RPMI in duplicate. Lower chamber of companion plate was filled with 600 μl antibiotic-free complete growth medium as a chemoattractant. Cells were incubated for a further 48 h at 37 °C. Inner surfaces of transwells were scrapped with a PBS-soaked cotton bud to remove non-migrated cells. Migrated cells were fixed with 4% paraformaldehyde in PBS for 10 min at RT, washed with PBS and stained with 0.4% crystal violet in 20% methanol for 45 min at RT. Membranes were washed in ddH_2_O and stored in PBS at 4 °C. A minimum of five fields per transwell membrane were imaged at ×10 magnification using the digital inverted microscope EVOS Cell Imaging System (Life Technologies, CA, USA). Total number of migrated cells was manually counted for each imaged field, the mean was calculated, and normalised to total protein content by SRB assay. Invasion assays were performed as above but with additional coating of inner transwell surfaces with 5 µg Matrigel.

### Biotinylated miR/AGO2 pulldown for miR target identification

Cells seeded in 2 × 10 cm dishes per condition were transfected with 200 pmol biotinylated miR mimic (Exiqon) per plate for 24 h prior to harvesting, pooling and lysis in 500 µl per pellet of ice-cold hypotonic lysis buffer (10 mM KCl, 1.5 mM MgCl_2_, 10 mM Tris-HCl pH7.5, 5 mM DTT, 0.5% NP40, 60 U/ml SUPERaseIn (Ambion), 5 µl/ml protease inhibitor cocktail). Lysates were passed through needles (2 × 23 G, 2 × 27 G) to ensure efficient lysis. Lysates were cleared by centrifugation at 5000×*g* for 5 min at 4 °C. 50 µl of cleared lysate was retained for input RNA extraction. 10 µg AGO2 antibody (Abcam ab57113) diluted in 200 µl hypotonic lysis buffer was conjugated to 50 µl Dynabeads Protein G (Invitrogen) per condition by incubation at room temperature for 20 min with rotation. AGO2-conjugated beads were washed 3× in hypotonic lysis buffer prior to incubation with lysate (500 µl) at room temperature for 30 min with rotation. Beads were then washed 3× with hypotonic lysis buffer and eluted in 50 µl elution buffer (Dynabeads Protein G IP kit, Invitrogen). NaCl was added to eluted immunoprecipitated AGO2 complex to a final concentration of 1 M. Reconstituted MyOne C1 streptavidin beads (LifeTech) were washed 2× in 0.1 M NaOH, 0.05 M NaCl, 1× in 0.1 M NaCl and 1× in hypotonic lysis buffer and blocked with 1 µg/µl BSA and 1 µg/µl yeast tRNA in hypotonic lysis buffer for 3 h at 4 °C with rotation. Blocked beads were washed 3× with hypotonic lysis buffer prior to incubation with immunoprecipitated AGO2 complex for 16 h at 4 °C. Beads were washed 3× with hypotonic lysis buffer and RNA extracted from both streptavidin beads and input lysates using 300 µl Trizol LS according to the manufacturer’s protocol. Isolated RNA was reverse transcribed and qPCR performed as described. RNase-free conditions were maintained throughout.

### AGO-PAR-CLIP-seq

AGO-PAR-CLIP-seq data represented is extracted from prior published data [31]. Methods are as previously published [31].

### Patient-derived xenograft (PDX) development CP50

The development of CP50 PDX has been described recently (Welti et al; submitted to *Clinical Cancer Research*). Briefly, a tissue biopsy from a patient with CRPC who had progressed through all standard of care treatments (docetaxel, cabazitaxel, enzalutamide and abiraterone) was implanted into 2 NOD scid gamma (NSG) (JAX Mice) male mice. Next generation sequencing of the parental lymph node biopsy and generated CP50 PDX demonstrated comparable copy number analysis, with chromosome 8 gain and androgen receptor amplification (Welti et al; submitted to *Clinical Cancer Research*). To develop a castrate line, CP50 PDX was castrated when tumours reached 300 to 400 mm^3^, and passaged again at a size of 1200 mm^3^ into castrate mice, this process was repeated for ongoing maintenance. The effect of enzalutamide on castrate CP50 PDX miR levels was determined at two time points. For both experiments, animals were treated daily by intraperitoneal injection with enzalutamide (10 mg/kg) or vehicle (Keptose HPB) when implanted tumours reached a size of 300 to 400 mm^3^. Six (*n* = 3 enzalutamide and *n* = 3 vehicle) were treated for 10 days (short term) and 12 (*n* = 6 enzalutamide and *n* = 6 vehicle) were treated until tumours reached a size 1200 mm^3^ (long term: 22–48 days).

### Statistical analyses

Normally distributed continuous variables were assessed by Student’s *t*-test. *P* ≤ 0.05 was interpreted to denote statistical significance. Data from miR inhibitor library screening were analysed using the B score method [53]. The simplest and most widely used statistical scoring method in high-content screening is the *Z* score, however, B score was used here as it additionally adjusts for positional effects within each 96-well plate. The expression of mRNA and miRNA in PC samples was analysed from publically available data sets GSE34932 and TCGA (mRNA and miRNA-seq). TCGA data (processed, and Log2 transformed) was accessed from https://tcga-data.nci.nih.gov/docs/publications/prad_2015/. IBM-SPSS statistics software was used for analysis of relationships between miR levels and target gene mRNA levels, and with clinical attributes. CRPC (*n* = 122) transcriptome data from SU2C-PCF [32] was re-analysed. Paired-end transcriptome sequencing reads were aligned to the human reference genome (GRCh37/hg19) using a RNA-seq spliced read mapper Tophat2 [54] (Tophat2.0.7), with default settings. Gene expression, fragments per kilobase of transcript per million mapped reads (FPKM), was calculated using Cufflinks [55]. AR activity score is an accumulation measurement of AR pathway activity based on 43 genes whose expression level is regulated by AR in PC cell lines [56] and metastatic PC [57].

## Supplementary information

Supplemental Data
