# Supplementary material for: Androgen receptor-modulatory microRNAs provide insight into therapy resistance and therapeutic targets in advanced prostate cancer
Source: Oncogene. 2019 May 1;38(28):5700–24. doi: 10.1038/s41388-019-0823-5 (PMC6755970; doi:10.1038/s41388-019-0823-5)
Supplement: Supplementary file 1 — Supplemental Data [file 41388_2019_823_MOESM1_ESM.pdf]

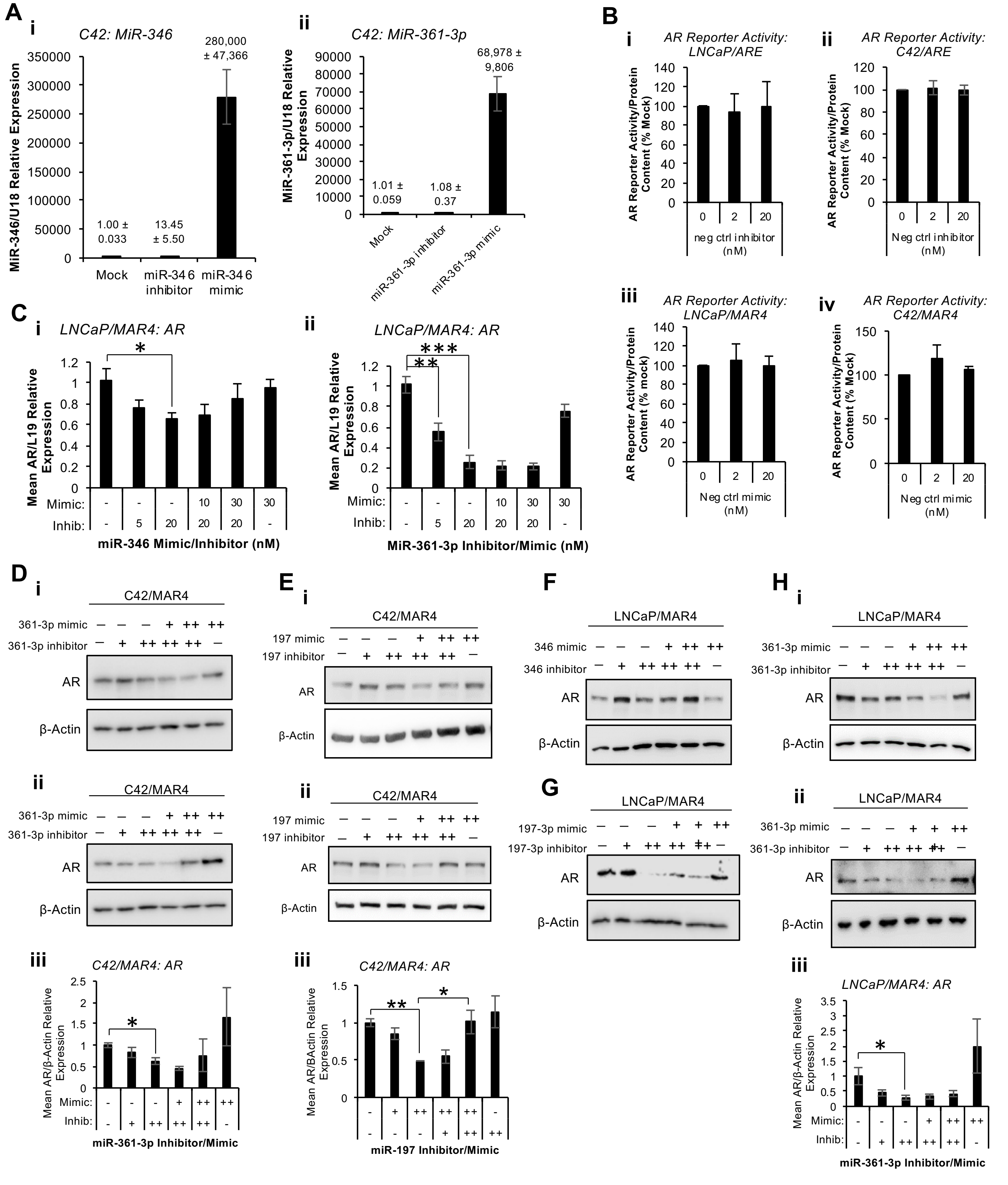

Figure S1: Validation of Effects of MiR-346, -361-3p and -197 on AR mRNA and Protein Levels. *A)* qRT-PCR analysis of *(i)* miR-346 or *(ii)* miR-361-3p levels in C42 cells transfected with *(i)* miR-346 or *ii)* miR-361-3p inhibitor or mimic for 24h. U18 was used for normaliation. *C)* qRT-PCR analysis of AR mRNA levels in LNCaP/MAR4 cells transfected with *(i)* miR-346 or *ii)* miR-361-3p mimic and/or inhibitor for 24h. L19 was used as a normalisation gene. *(A-C) Columns:* mean ± SEM for three independent experiments performed in triplicate *(A,C)* or duplicate *(B)*. *D-H)* Western blot analysis of AR protein levels in C42/MAR4 *(D,E)* or LNCaP/MAR4 cells *(F-H)* transfected with *(D,H)* miR-361-3p, *(E,G)* miR-197 or *(F)* miR-346 mimic and/or inhibitor for 24h. β-actin was used as a control for loading. Densitometry was performed using Image J software and relative protein levels are displayed *(iii)*.Independent biological replicates relating to Fig 2B are shown. *Columns:* mean relative protein levels ± SEM for three independent experiments. * $P \leq 0.05$, ** $P \leq 0.005$, *** $P \leq 0.0001$. See also Fig 1, 2.

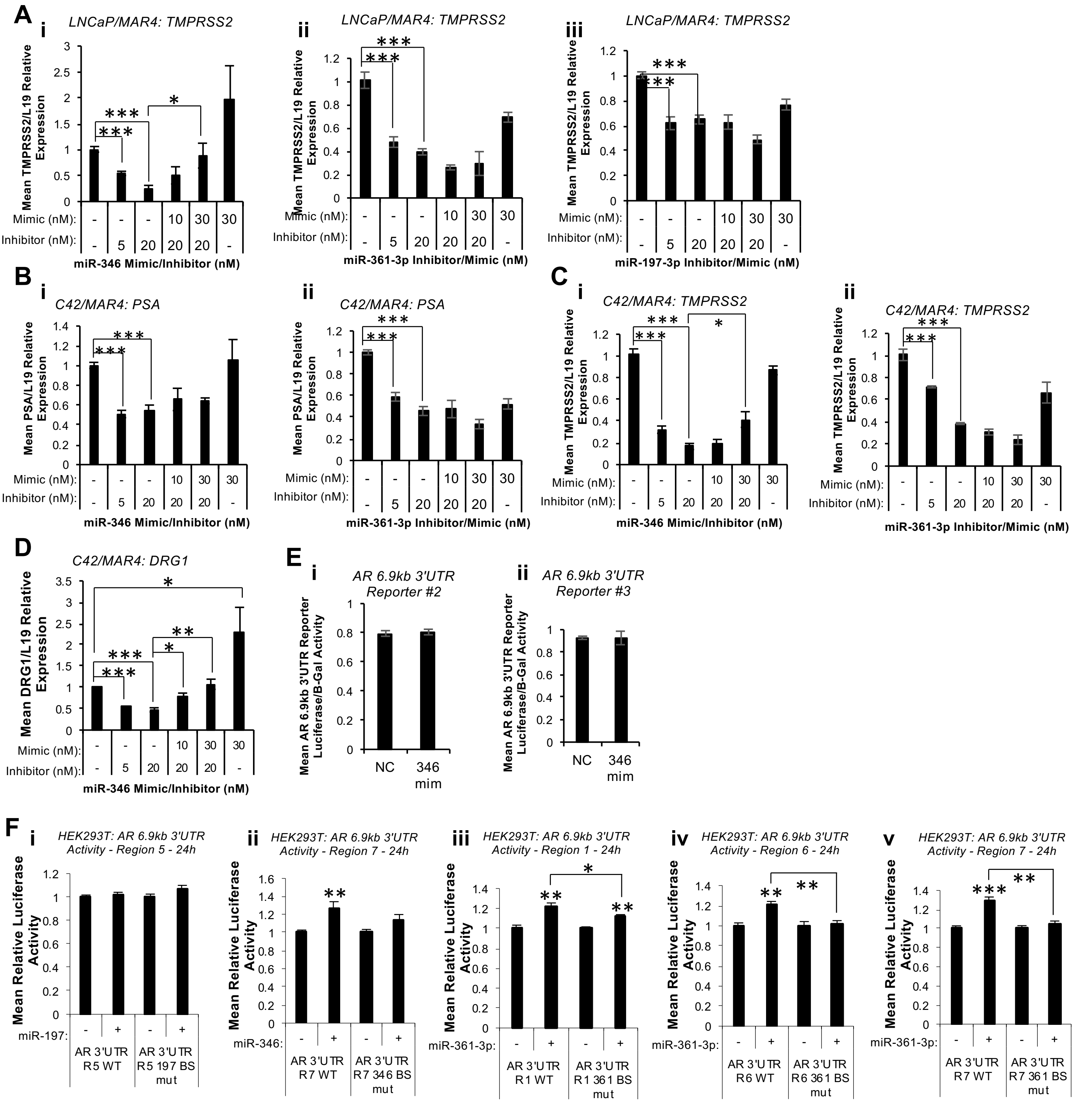

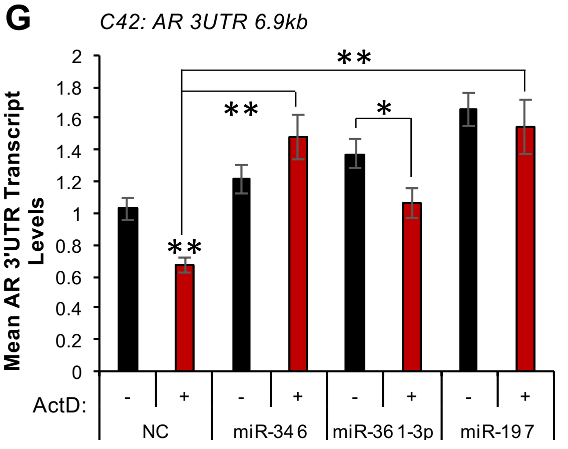

Figure S2: MiR-346, -361-3p and -197 Increase AR Activity in Part Through Association with AR 3'UTR. *A)* qRT-PCR analysis of TMPRSS2 mRNA levels in LNCaP/MAR4 cells transfected with *(i)* miR-346, *ii)* miR-361-3p or *(iii)* miR-197 mimic and/or inhibitor for 24h. *(B,C,D)* qRT-PCR analysis of *(B)* PSA, *(C)* TMPRSS2 or *(D)* DRG1 mRNA levels in C42/MAR4 cells transfected with *(i)* miR-346 or *ii)* miR-361-3p mimic and/or inhibitor for 24h. *(B,C,D)* L19 was used as a normalisation gene, *columns:* mean ± SEM for three independent experiments performed in triplicate. *(E,F)* Luciferase assay analysis of 6.9kb AR 3'UTR activity in HEK293T cells transfected with pMiR-Report vector containing WT *(E)* or miR binding site-mutant *(F)* regions of the AR 6.9kb 3'UTR as depicted *(Fig 2Di)* ± miR mimics (20nM) for 48h *(E)* or 24h *(F)*. Luciferase activity was normalised to β-galactosidase activity to correct for transfection efficiency. *Columns:* mean normalised AR reporter luciferase activity from three independent experiments performed in duplicate ± SEM. *(G)* qRT-PCR analysis of AR 6.9kb 3'UTR in C42 cells following 24h transfection of miR mimics (20nM) and Actinomycin D treatment (1μM, 4h). *Columns:* mean ± SEM for three independent experiments performed in triplicate. * $P \le 0.05$, ** $P \le 0.005$, *** $P \le 0.0001$.

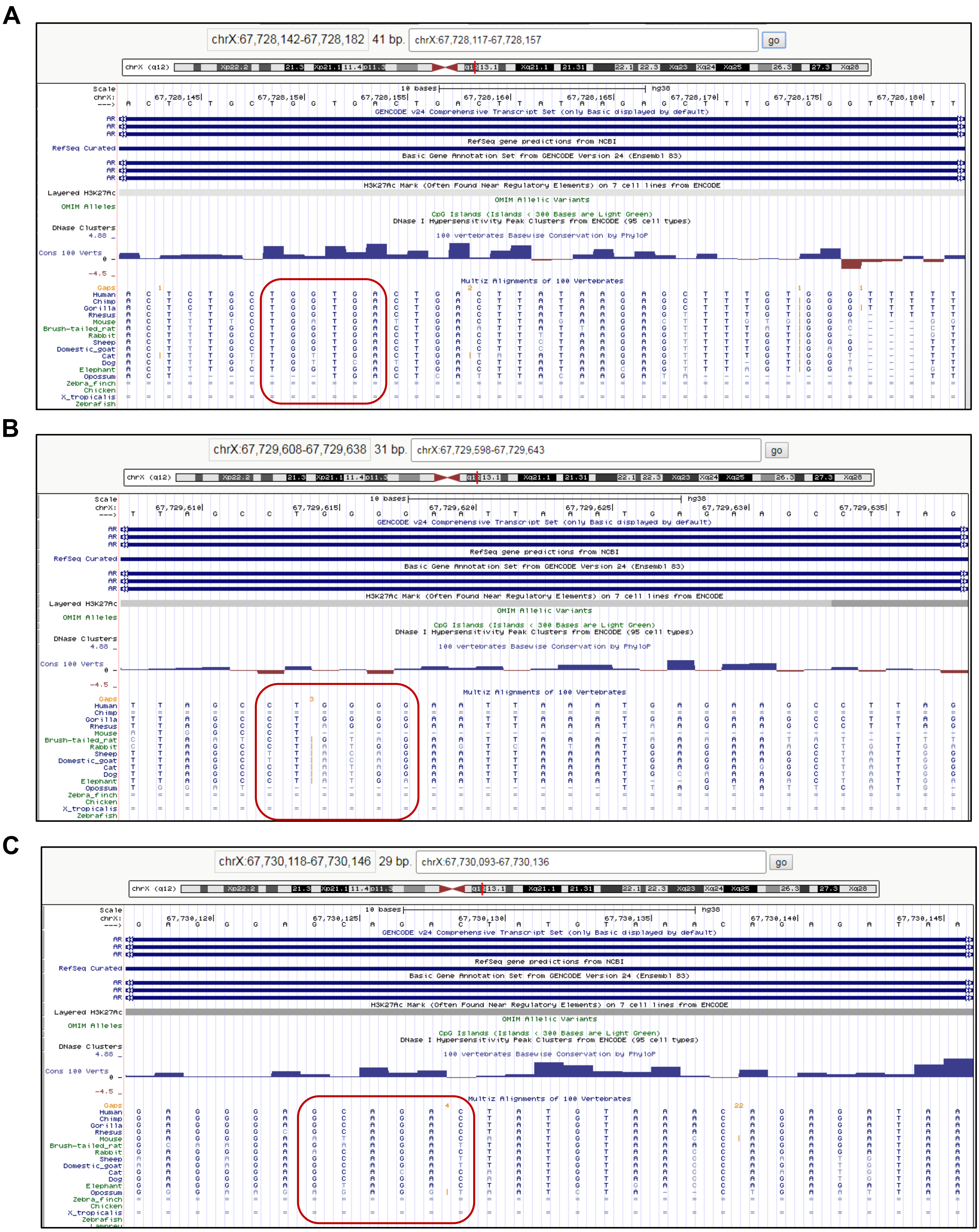

Fig S3: MiR-197 Binding Site at 4308-4313nt of AR 3'UTR is Highly Conserved Across Species. *(A,B,C)* UCSC genome browser analysis of miR binding sites across mammalian and non-mammalian species for *A)* miR-197 binding site at 4308-4313nt of AR 3'UTR, *B)* miR-361-3p binding site at 5772-5777nt of AR 3'UTR and *C)* miR-346 binding site at 6283-6288nt of AR 3'UTR. MiR seed binding site sequence is indicated by red box.

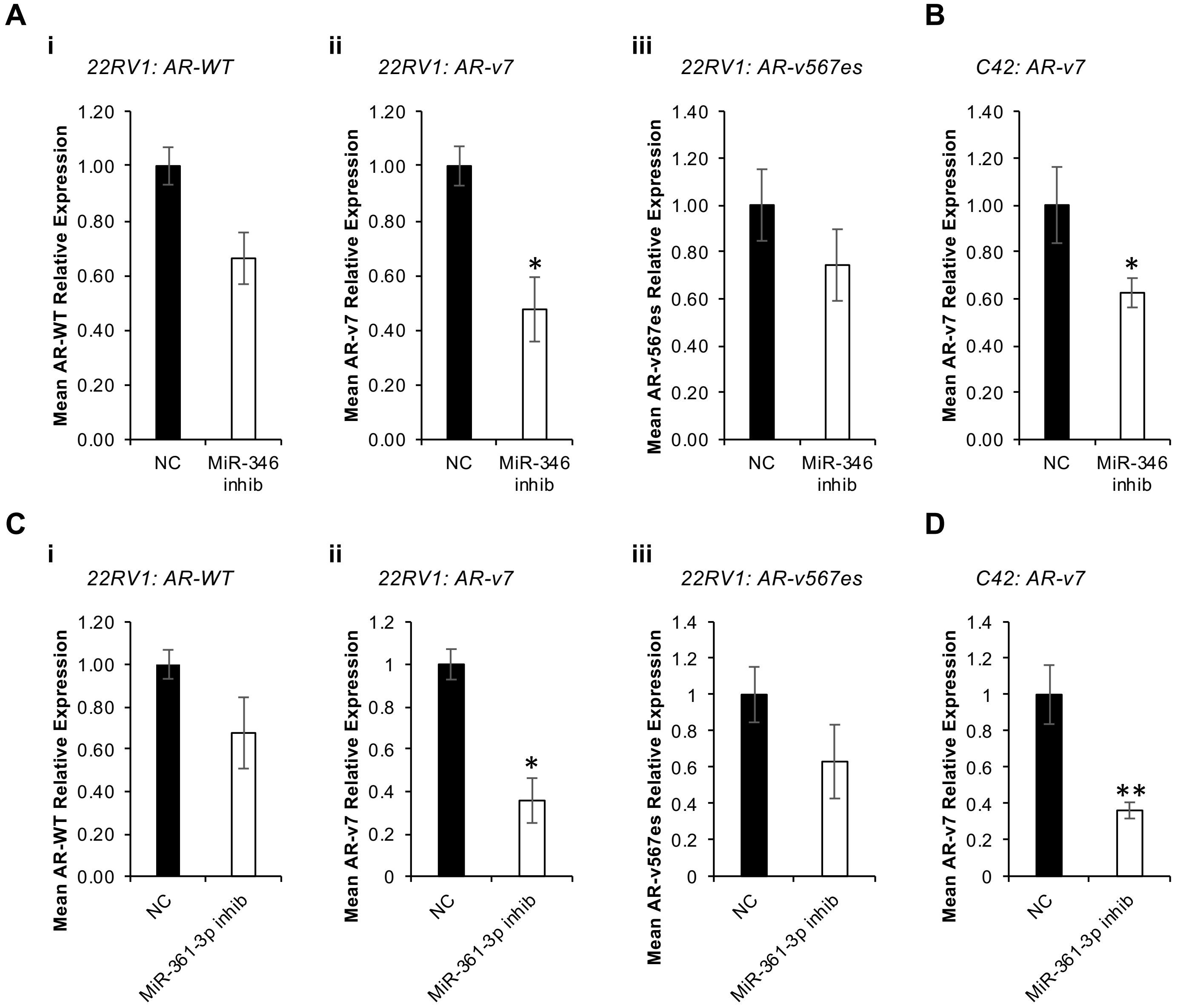

Figure S4: MiR-346 and MiR-361-3p Alter Levels of AR Variants in Prostate Cancer Cells. *A-D)* qRT-PCR analysis of wild-type AR *(i),* AR-v7 *(ii,iv)* and AR-v567es *(iii)* mRNA levels in 22RV1 *(A,C)* and C42 *(B,D)* cells transfected with *(A,B)* miR-346 or *(C,D)* miR-361-3p inhibitor (both 5nM) for 24h. Mean of L19 and GAPDH was used for normalisation. *Columns:* mean ± SEM for three independent experiments performed in triplicate. * $P \leq 0.05$, ** $P \leq 0.005$. See also Fig 2.

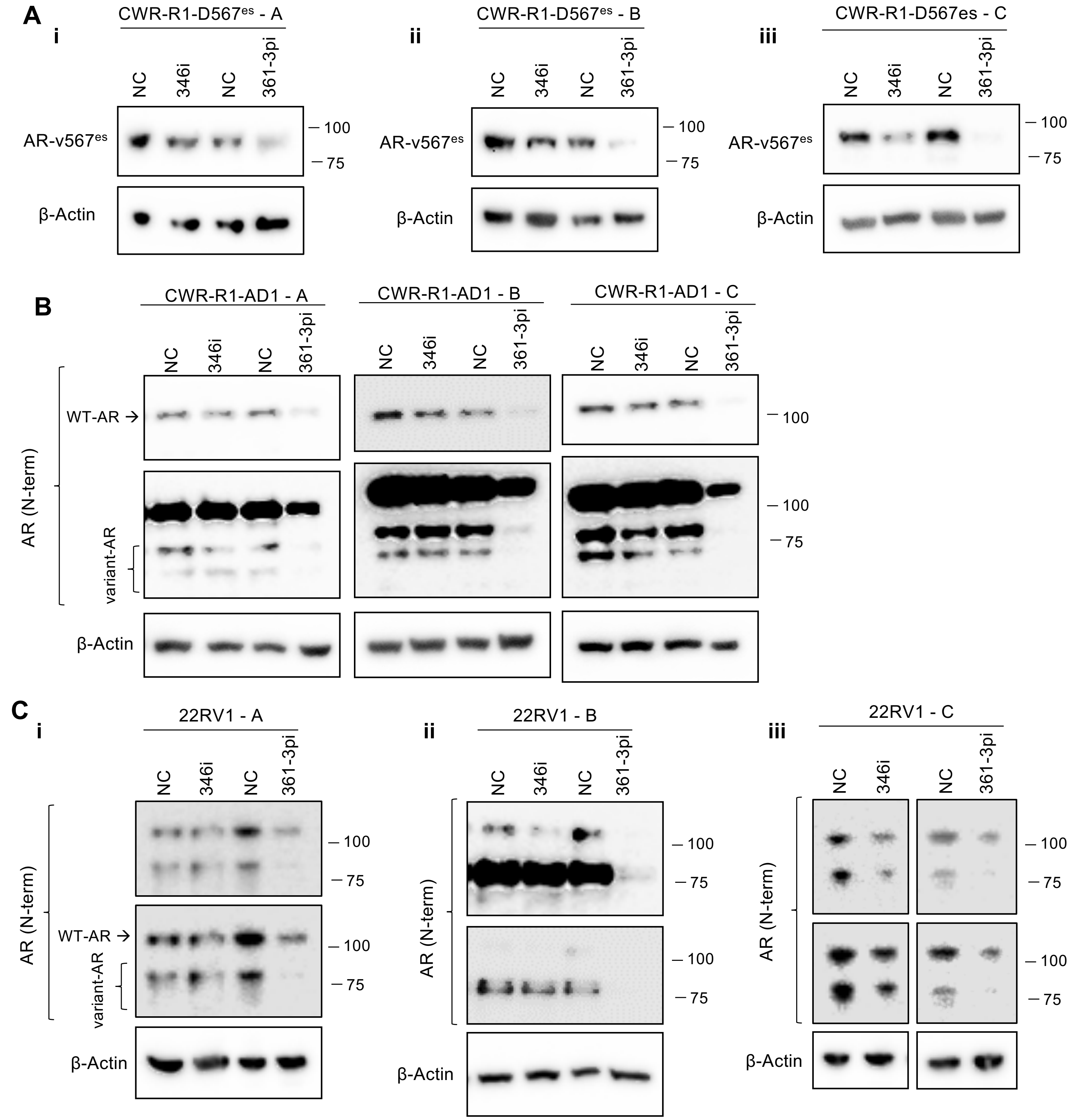

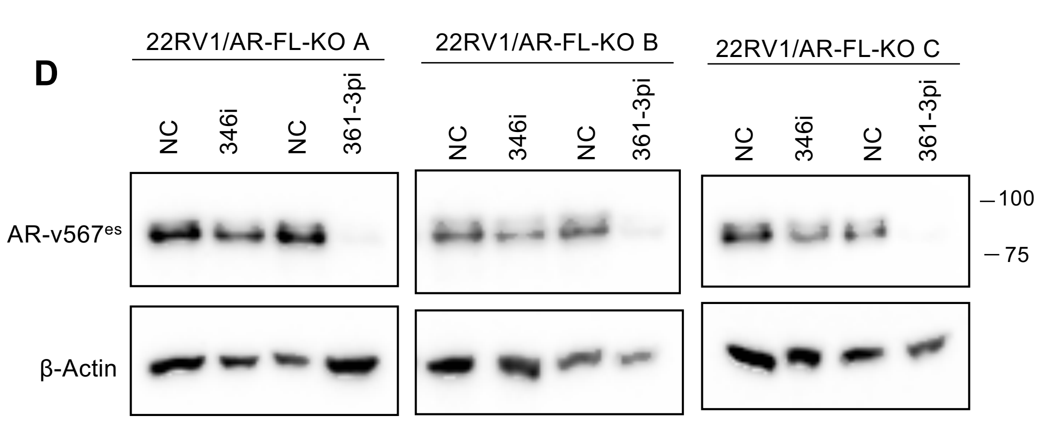

Figure S5: MiR-346 and MiR-361-3p Inhibitors Alter Protein Levels of AR Variants in Prostate Cancer Cells. *A-D)* Western blot analysis of wild-type AR (110kDa) and variant AR (65-90kDa) in *(A)* CWR-R1-D567es (lacking WT-AR but overexpressing v567es), *(B)* CWR-R1-AD1 (parental cells expressing WT- and variant-AR), *(C)* 22RV1 (expressing WT- and variant-AR) and *(D)* 22RV1/AR-FL-KO cells (expressing AR-variants but with WT-AR knocked out) cells transfected with miR-346 or miR-361-3p inhibitor (both 20nM) for 24h. β-actin was used as a control for loading. Three independent biological replicates are shown. See also Fig 2.

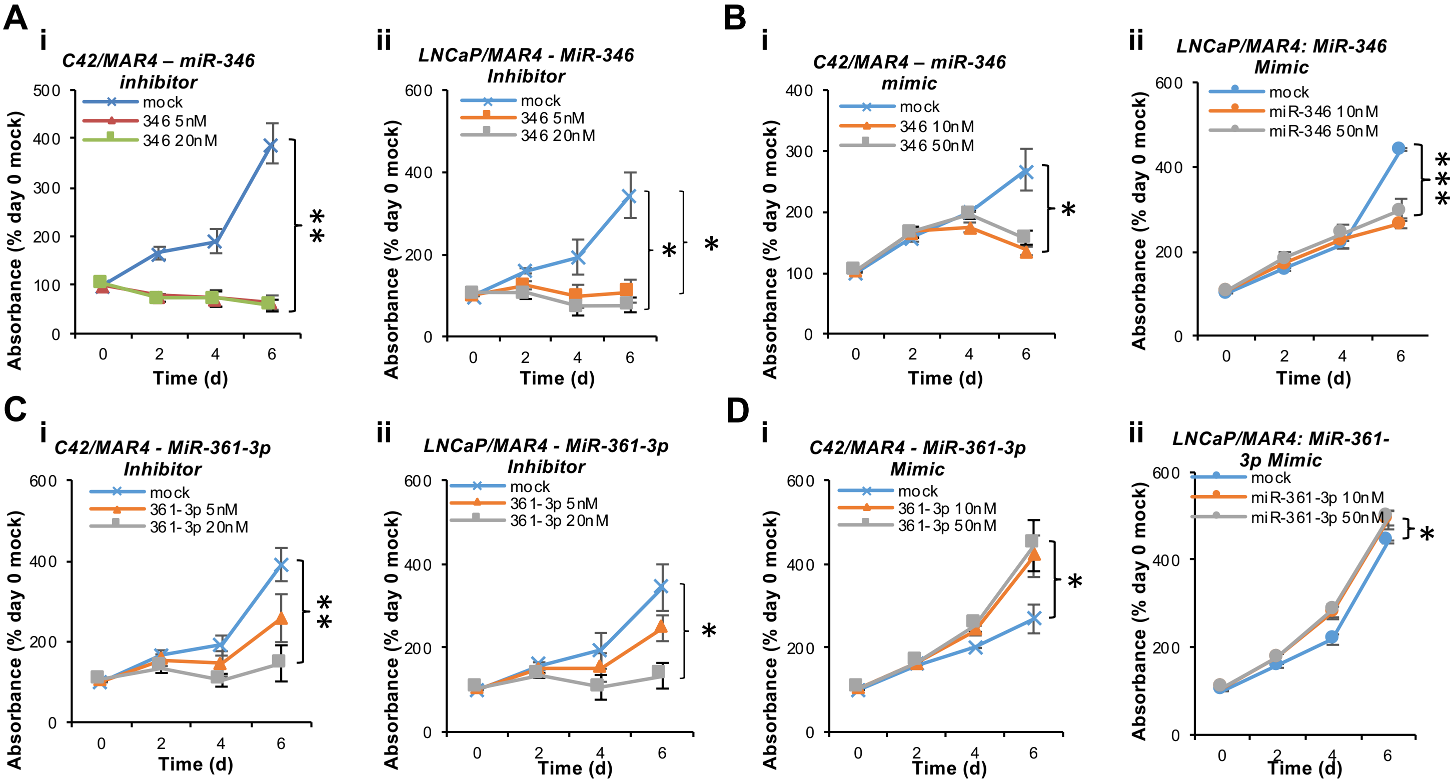

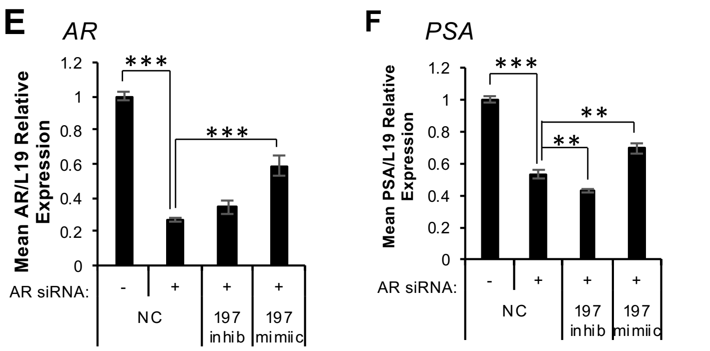

Figure S6: MiR-346, -361-3p and -197 Modulate PC Cell Proliferation, and Inhibition Shows Additive Effects with AR Silencing. *(A-D)* SRB proliferation assay analysis of C42/MAR4 *(Ai,Bi,Ci,Di)* or LNCaP/MAR4 *(Aii,Bii,Cii,Dii)* cells transfected with: *(A)* miR-346 inhibitor (5 and 20nM), *(B)* miR-346 mimic (10 and 50nM), *(C)* miR-361-3p inhibitor (5 and 20nM) or *(D)* miR-361-3p mimic (10 or 50nM) for 6 days. Data are presented relative to absorbance at day 0. *Points:* mean absorbance at 492nm for three independent experiments performed in quadruplicate ± SEM. *E-H)* qRT-PCR analysis of *(E)* AR, *(F)* PSA, *(G)* TMPRSS2 and *(H)* KLK2 mRNA levels in LNCaP/ARsiRNA cells transfected with miR-197 *(E,F)*, miR-346 *(Gi,Hi)* or miR-361-3p *(Gii,Hii)* inhibitor or mimic (20nM) ± Doxycycline (1μM) for 24h. L19 was used as a normalisation gene. *Columns:* mean ± SEM for three independent experiments performed in triplicate. * $P \leq 0.05$, ** $P \leq 0.005$, *** $P \leq 0.0001$. See also Fig 3.

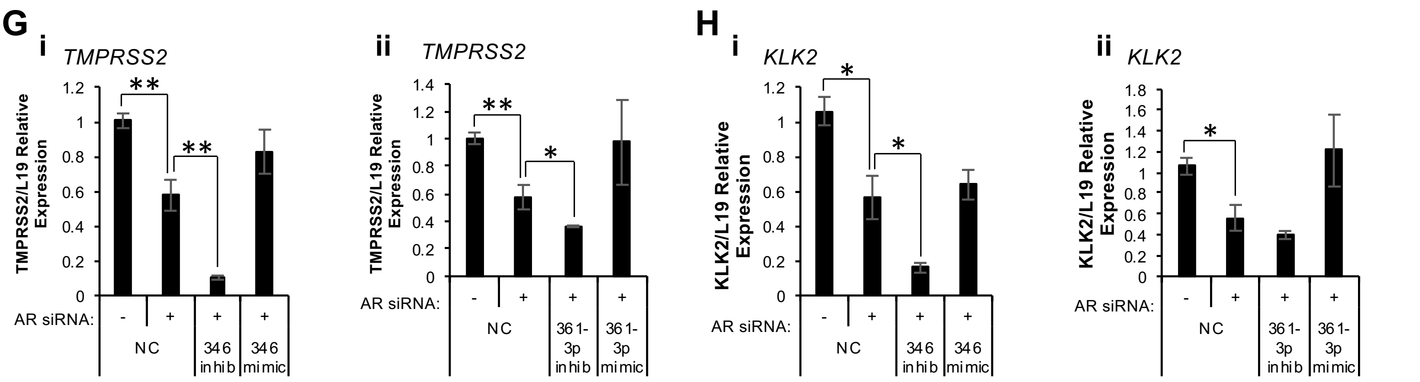

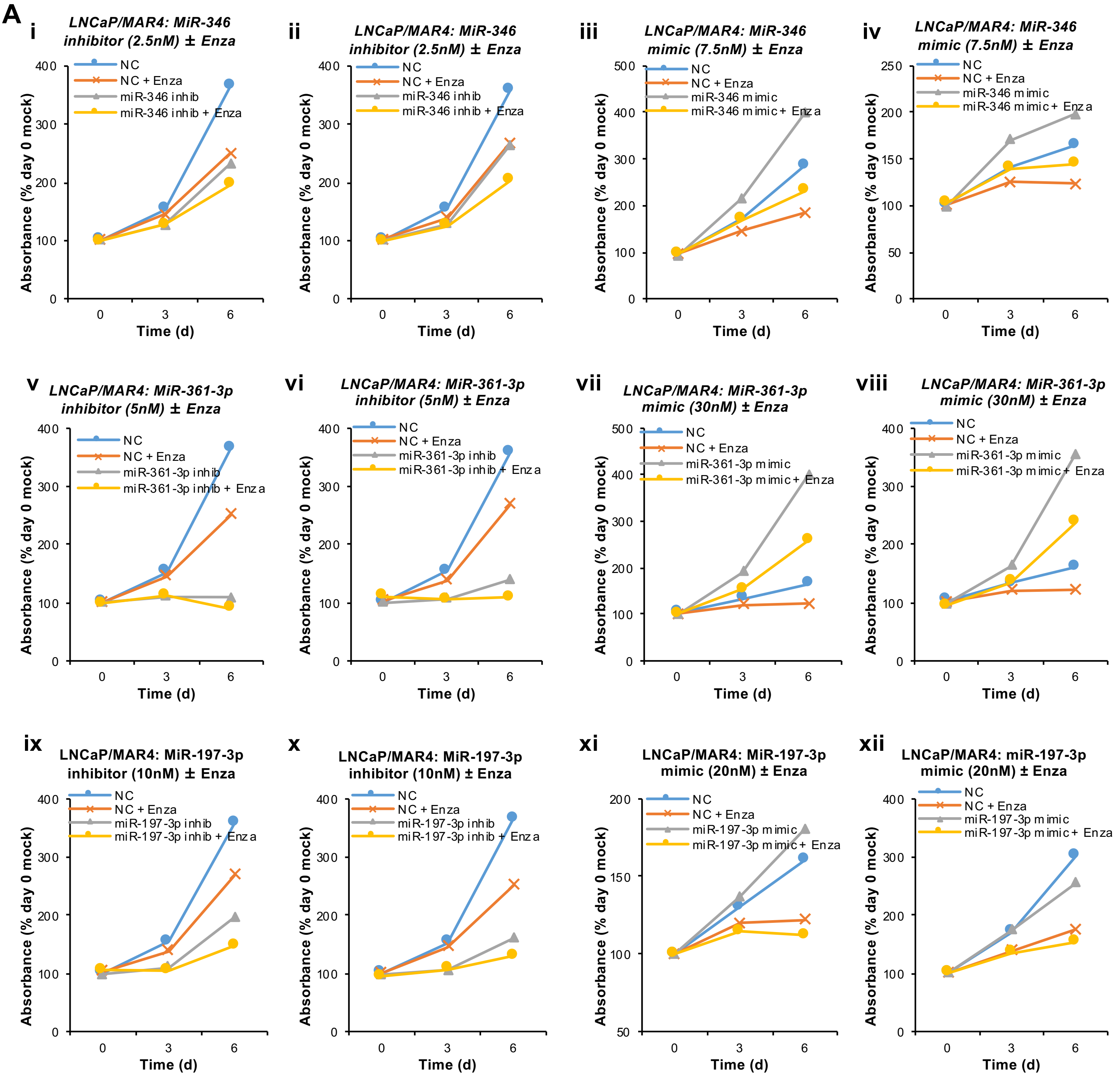

Figure S7: Inhibitors of MiR-346, -361-3p and -197 Show Additive Effects with Enzalutamide Treatment in Prostate Cancer Cells. SRB proliferation assay analysis of LNCaP/MAR4 cells transfected with miR-346 inhibitor *(i,ii* – 2.5nM*)*, miR-346 mimic *(iii,iv* – 7.5nM*)*, miR-361-3p inhibitor *(v,vi* – 5nM*)*, miR-361-3p mimic *(vii,viii* – 30nM), miR-197-3p inhibitor *(ix,x* - 10nM), or miR-197-3p mimic *(xi,xii* – 20nM*)* ± Enzalutamide (0.8µM) for 6 days. Data are presented relative to absorbance at day 0 and two additional biological replicates are shown. Representative results are shown in Fig 4.

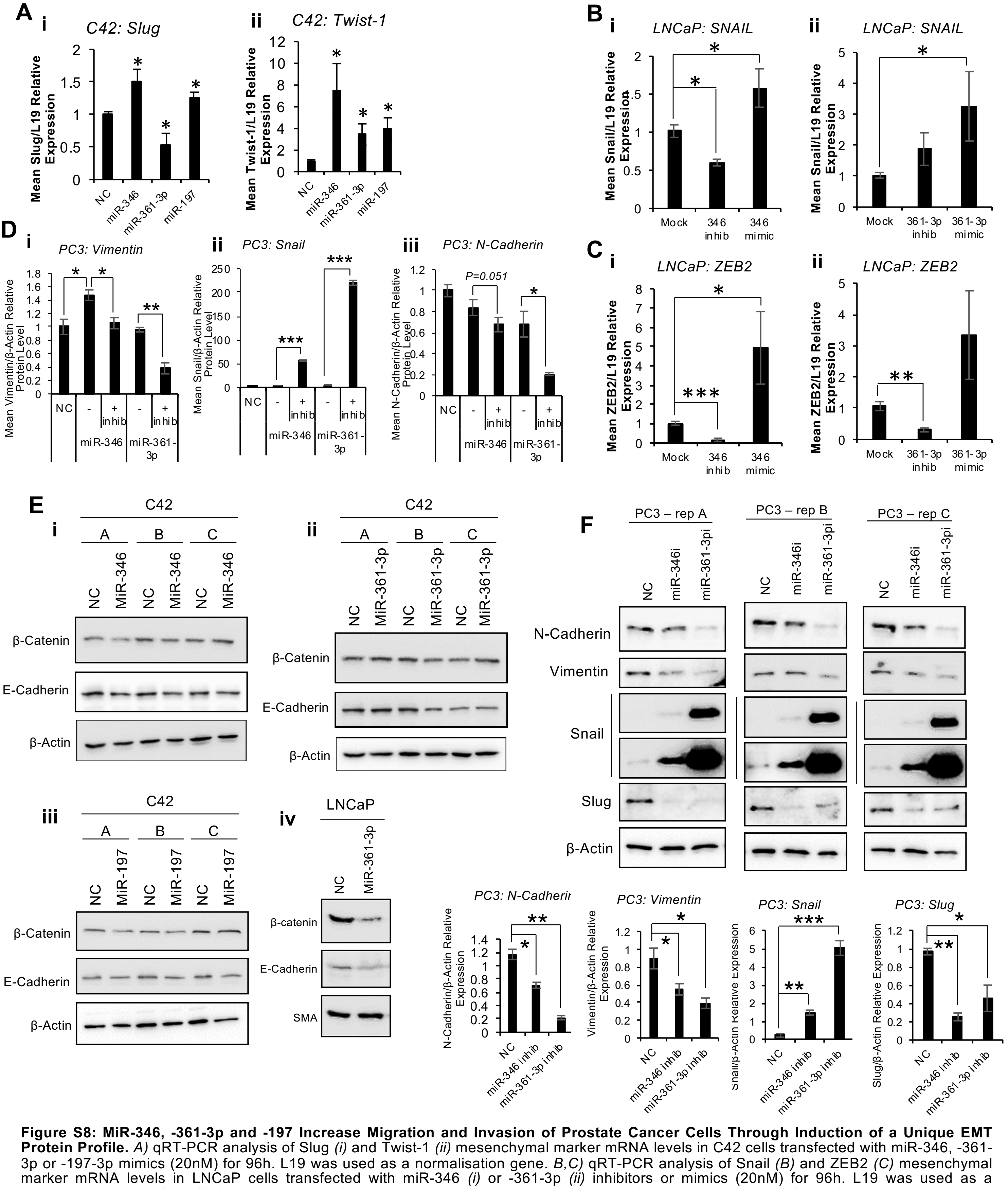

Figure S8: MiR-346, -361-3p and -197 Increase Migration and Invasion of Prostate Cancer Cells Through Induction of a Unique EMT Protein Profile. *A)* qRT-PCR analysis of Slug *(i)* and Twist-1 *(ii)* mesenchymal marker mRNA levels in C42 cells transfected with miR-346, -361-3p or -197-3p mimics (20nM) for 96h. L19 was used as a normalisation gene. *B,C)* qRT-PCR analysis of Snail *(B)* and ZEB2 *(C)* mesenchymal marker mRNA levels in LNCaP cells transfected with miR-346 *(i)* or -361-3p *(ii)* inhibitors or mimics (20nM) for 96h. L19 was used as a normalisation gene. *(A,B,C) Columns:* mean ± SEM for three independent experiments performed in triplicate. *D)* Quantification of Western blot analysis of *(i)* Vimentin, *(ii)* Snail and *(iii)* N-Cadherin protein levels in PC3 cells transfected with miR-346 or -361-3p mimic (20nM) ± inhibitor (20nM) for 96h. β-actin was used as a control for loading. Densitometry was performed using Image J software. *Columns:* mean relative protein levels from three independent experiments ± SEM. *E)* Western blot analysis of β-Catenin and E-Cadherin protein levels in C42 *(i,ii,iii)* and LNCaP *(iv)* cells transfected with miR-346 *(i),* miR-361-3p *(ii,iv)* or miR-197 *(iii)* mimic for 72h. β-actin was used as a control for loading. Representative blots of three independent experiments are shown. Densitometry was performed using Image J software and relative protein levels are displayed *(*Fig 5E*ii). F)* Western blot analysis of mesenchymal marker proteins N-Cadherin, Vimentin, Snail and Slug in PC3 cells transfected with miR-346 or -361-3p inhibitor (20nM) for 96h. β-actin was used as a control for loading. Results of three independent experiments are shown. Densitometry was performed using Image J software and relative protein levels are displayed. *Columns:* mean relative protein levels from three independent experiments ± SEM. * $P \le 0.05$, ** $P \le 0.005$, *** $P \le 0.0001$. See also Fig S5.

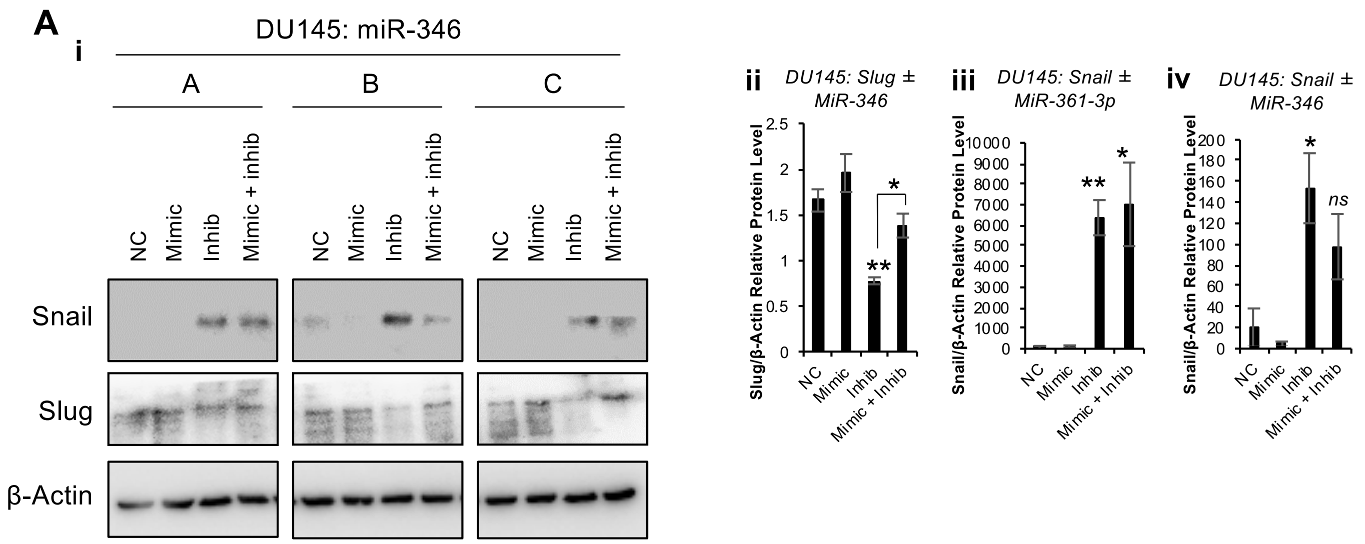

Figure S9: Inhibition of MiR-346 Increases Snail Protein Levels in DU145 Prostate Cancer Cells. *A)* Western blot analysis of mesenchymal marker proteins, Snail and Slug, in DU145 cells transfected with miR-346 mimic (20nM) and/or inhibitor (20nM) for 48h. β-actin was used as a control for loading. Results of three independent experiments are shown. Densitometry was performed using Image J software and relative protein levels ± SEM of thee independent experiments are displayed *(ii,iii,iv)*. * *P* ≤ 0.05, ** *P* ≤ 0.005. See also Fig 5.

A

Chrom.	Binding Location	Strand	Gene	Seed Start	Seed End	Seed Type	MiR	Total Occur	No. PC lines
chr3	CDS	-	GSK3B	119582270	119582278	8mer-1a	197	13	4
chr16	Promoter	-	PKCμ	2156693	2156700	7mer-m8	197	7	4
chr1	CDS	-	PARP1	226555951	226555958	7mer-1a	361-3p	5	4
chr16	CDS	-	PKCμ	2140195	2140202	7mer-m8	361-3p	5	4
chr1	3_UTR	-	PARP1	226548831	226548839	8mer-1a	346	3	3
chr12	3_UTR	+	MDM2	69236143	69236150	7mer-1a	346	3	1
chr1	CDS	-	PARP1	226570817	226570824	7mer-m8	361-3p	3	3
chr13	3_UTR	-	LATS2	21548863	21548870	7mer-1a	197	2	2
chr16	3_UTR	-	PKCμ	2139089	2139097	8mer-1a	346	1	1
chr3	3_UTR	-	GSK3B	119544271	119544278	7mer-1a	361-3p	1	1

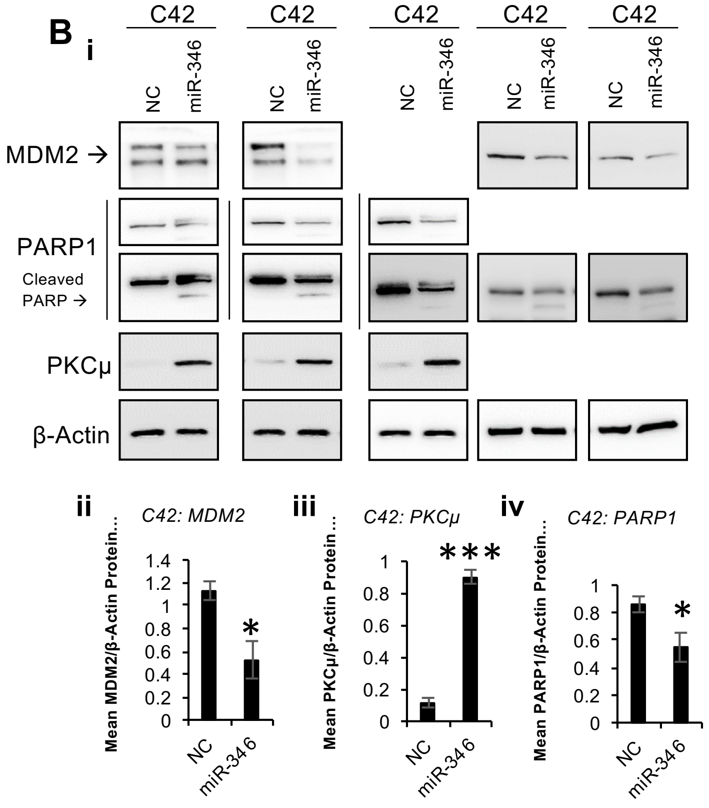

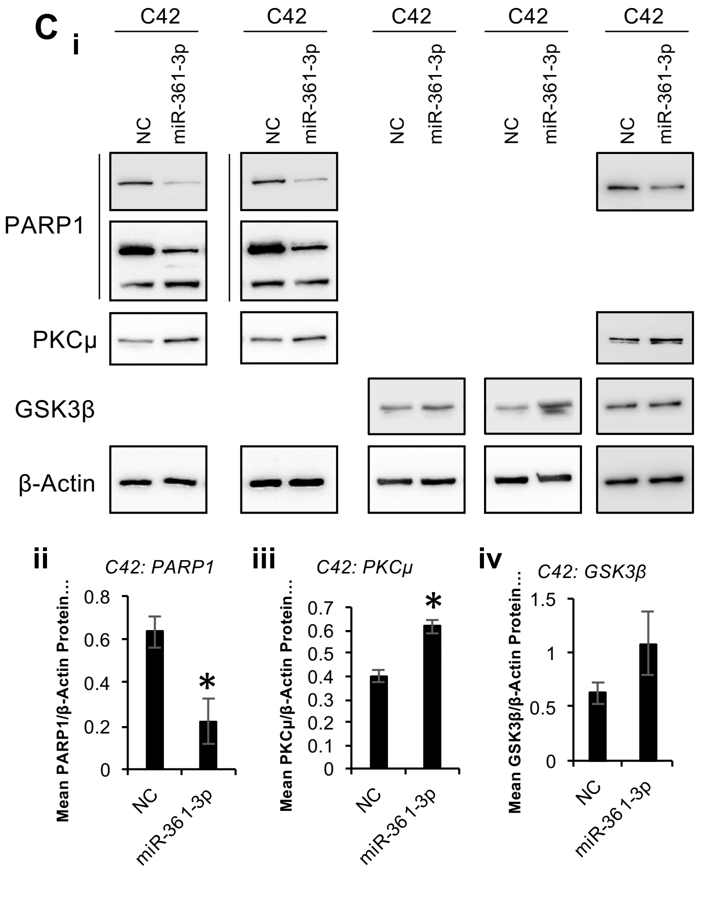

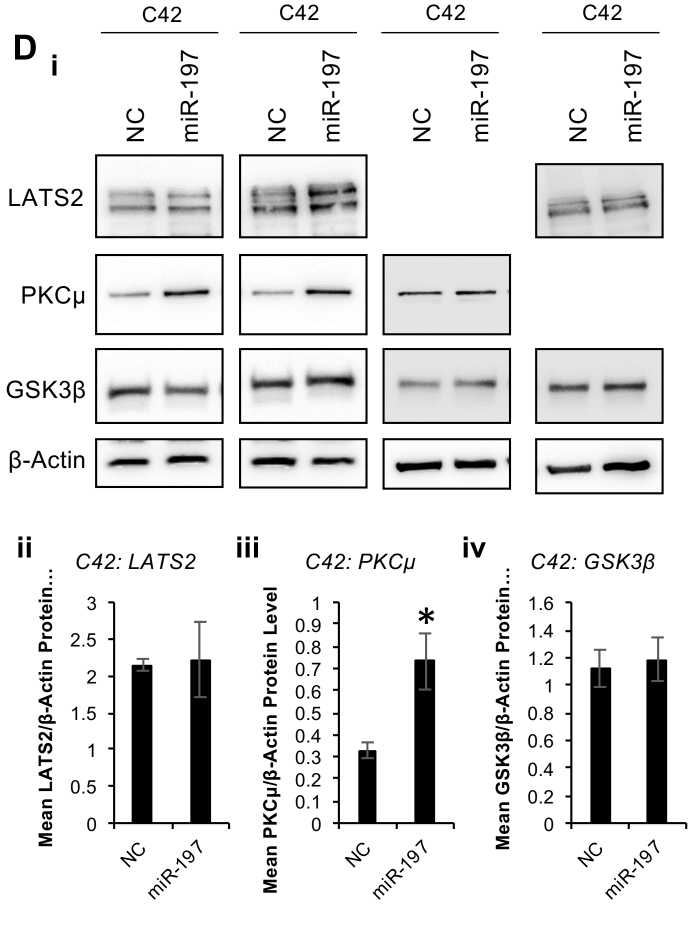

Figure S10: MiR-346, -361-3p and -197 Target Snail-Regulatory Proteins to Induce a Unique, Pro-Invasive EMT Protein Profile. *A)* Table of AGO-PAR-CLIP-identified Snail-regulatory targets of miR-346, -361-3p and -197 in prostate cancer cell lines. AGO-PAR-CLIP-seq data represented is extracted from prior published data[25] *B,C,D)* Western blot analysis of Snail-regulatory proteins in C42 cells transfected with miR-346 *(B)*, -361-3p *(C)* or -197-3p *(D)* mimic (20nM) for 96h. β-actin was used as a control for loading. Images from replicate experiments are shown. Densitometry was performed using Image J software and mean relative protein levels ± SEM from 3-5 independent experiments are displayed *(ii,iii,iv)*. * $P \leq 0.05$, ** $P \leq 0.005$, *** $P \leq 0.0001$. See also Fig 5.

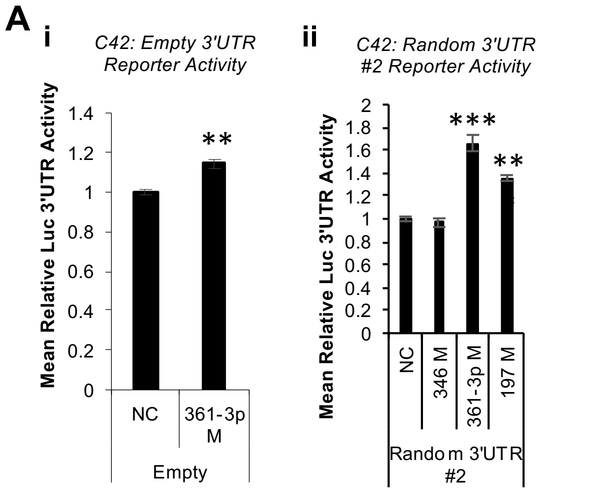

Figure S11: MiR-346, -361-3p and -197 Directly Target ARHGDIA, TAGLN2 and YWHAZ in Prostate Cancer Cells. *A)* Luciferase 3'UTR reporter assay analysis of C42 cells transfected with empty pLightSwitch *(i)* or pLightSwitch-Random-3'UTR #2 *(ii)* ± miR-346, -361-3p or -197-3p, as appropriate, for 48h. *B)* Luciferase 3'UTR reporter assay analysis of HEK293T cells transfected with pLightSwitch *(i)*, pLightSwitch-random-3'UTR *(ii)*, pLightSwitch-ARHGDIA-3'UTR *(iii)*, pLightSwitch-TAGLN2-3'UTR *(iv)* or pLightSwitch-YWHAZ-3'UTR *(v)* ± miR-346, -361-3p or -197-3p mimic and/or inhibitor for 48h. *(A,B)* Luciferase activity was normalised to β-galactosidase activity to correct for transfection efficiency, and data are displayed relative to negative control miR-transfected cells. *Columns:* mean relative luminescence from three independent experiments performed in duplicate ± SEM. *C)* Western blot analysis of TAGLN2, ARHGDIA and YWHAZ protein levels in C42 cells transfected with miR-346 *(i)*, -361-3p *(ii)* or -197 *(iii)* mimic (20nM) for 96h. β-actin was used as a control for loading and results of three independent experiments are shown. Densitometry was performed using ImageJ software and mean fold change relative to negative control-transfected cells ± SEM is shown *(iv)*. * $P \leq 0.05$, ** $P \leq 0.005$, *** $P \leq 0.0001$. *D)* Western blot analysis of ARHGDIA, TAGLN2 and YWHAZ protein levels in LNCaP cells transfected with siRNAs (1 or 10nM) targeting *i)* YWHAZ, *ii)* TAGLN2 or *iii)* ARHGDIA for 72h. β-actin was used as a control for loading.

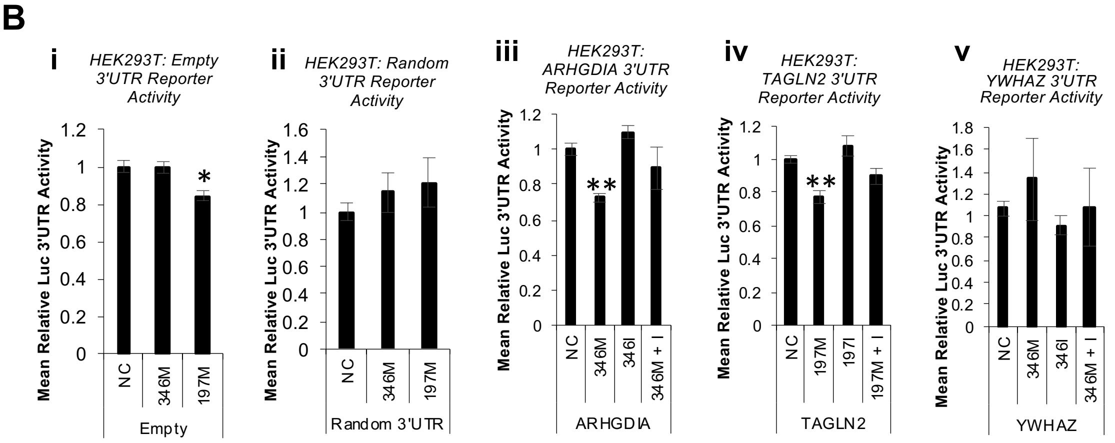

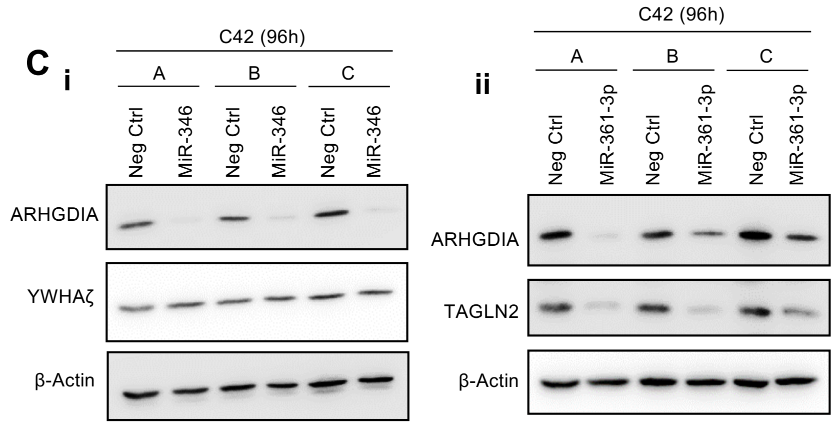

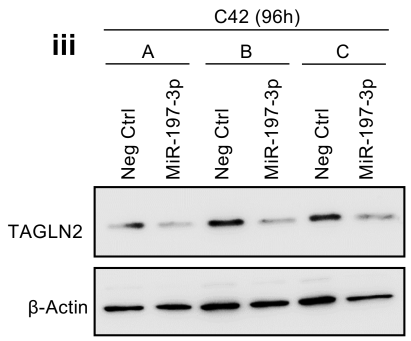

iv

	MiR-346	MiR-361-3p	MiR-197
ARHGDIA	0.259 ± 0.048 *	0.413 ± 0.079 *	-
TAGLN2	-	0.308 ± 0.103 **	0.438 ± 0.062 *
YWHAZ	1.18 ± 0.088	-	-

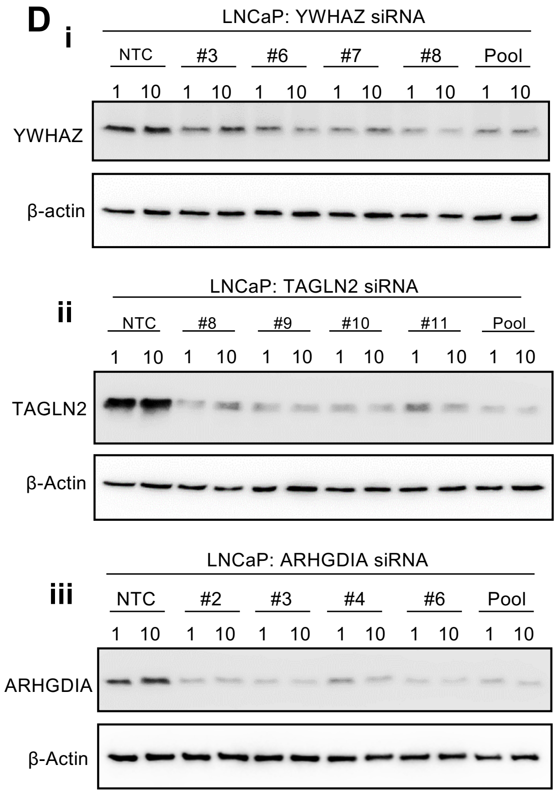

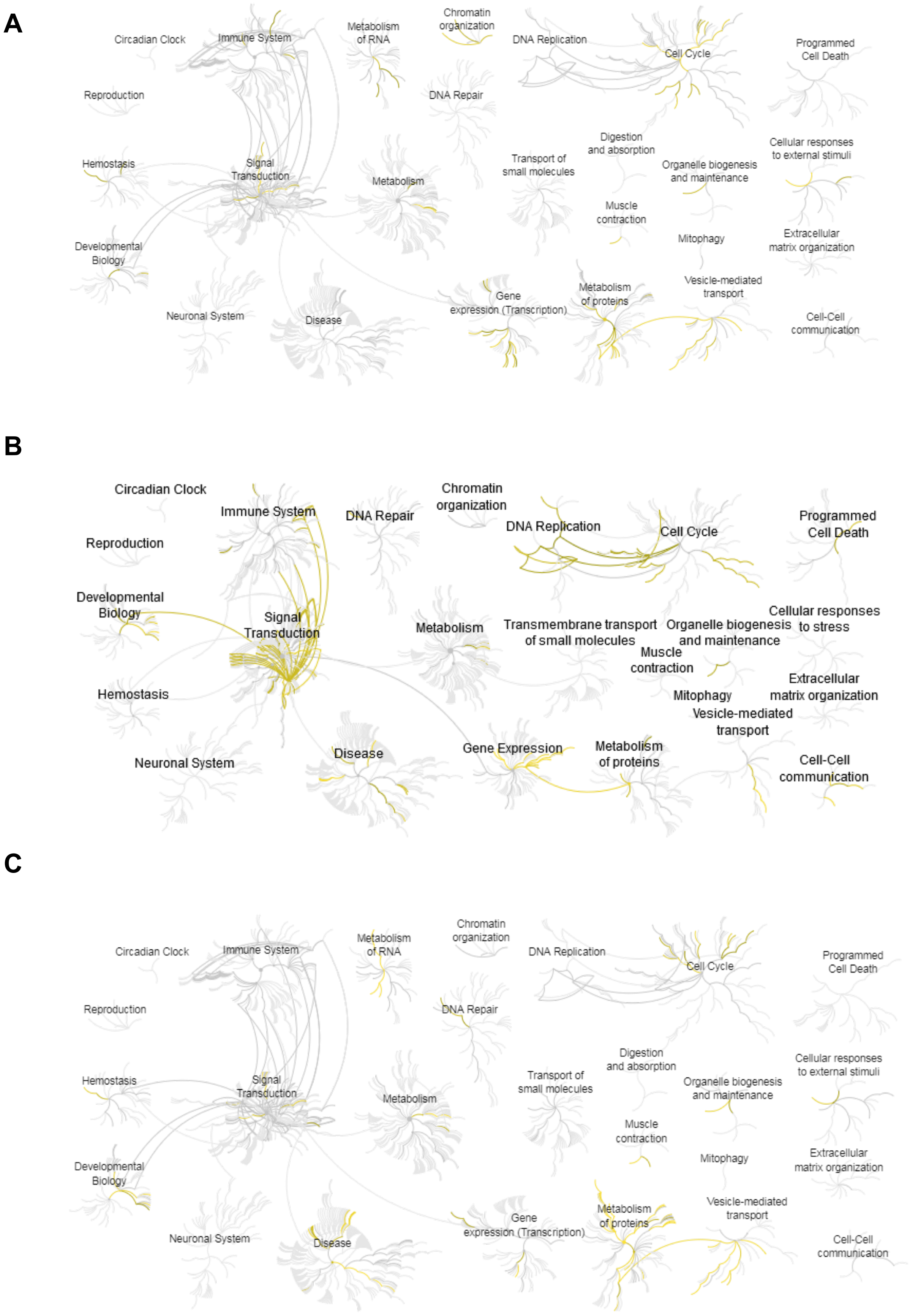

Figure S12: MiR-346, -361-3p and -197 Modify Diverse Biological Processes in Prostate Cancer. Diagram illustrating gene ontology pathway analysis of AGO-PAR-CLIP-identified miR-346 *(A)*, miR-361-3p *(B)* and miR-197 *(C)* targets in prostate cancer. See Fig 6.

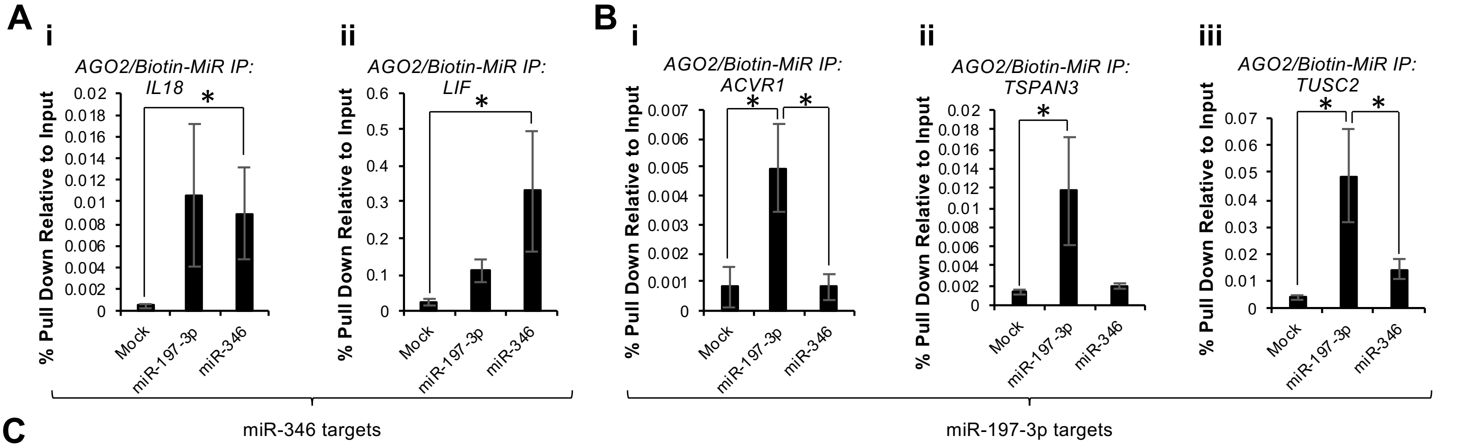

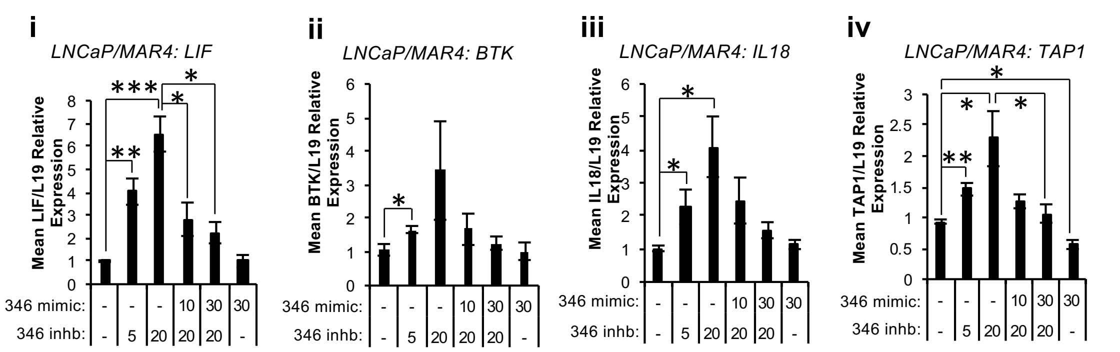

Figure S13: Validation of Additional MiR-346, -361-3p and -197 Targets in Prostate Cancer. *A,B)* AGO2/biotin-miR RNA-IP analysis of miR-346 and miR-197 association with miRTarBase-predicted miR-346 targets *(A* – IL18 and LIF) and miR-197 targets (*B* – ACVR1, TSPAN3 and TUSC2 3'UTRs. 22RV1 cells were transfected with biotin-labelled miR (200pmoles) for 24h, followed by two-step immunoprecipitation with AGO2 antibody- and streptavidin-coated beads. RNA was extracted from input and IP samples and qRT-PCR performed for predicted miR targets as shown. Data are presented relative to input values. *Columns:* mean pulldown relative to input from three independent experiments ± SEM. *C)* qRT-PCR analysis of *(i)* LIF, *(ii)* BTK, *(iii)* IL18 and *(iv)* TAP1 mRNA levels in LNCaP/MAR4 cells transfected with miR-346 mimic (0, 10, 30nM) and/or miR-346 inhibitor (0, 5, 20nM) for 24h. L19 was used as a normalisation gene. *Columns:* mean relative mRNA levels for three independent experiments performed in triplicate ± SEM. *D,E)* Western blot analysis of BTK and LIF protein levels in *(D)* C42/MAR4 and *(E)* LNCaP/MAR4 cells transfected with miR-346 inhibitor and/or mimic as above for 48h. β-Actin was used as a control for loading. *F,G)* qRT-PCR analysis of miR-346 *(F)* and miR-361-3p targets in C42/MAR4 cells transfected with miR-346 *(F)* or miR-361-3p *(G)* mimic (0, 10, 30nM) and/or inhibitor (0, 5, 20nM) for 24h. L19 was used as a normalisation gene. *Columns:* mean relative mRNA levels for three independent experiments performed in triplicate ± SEM.

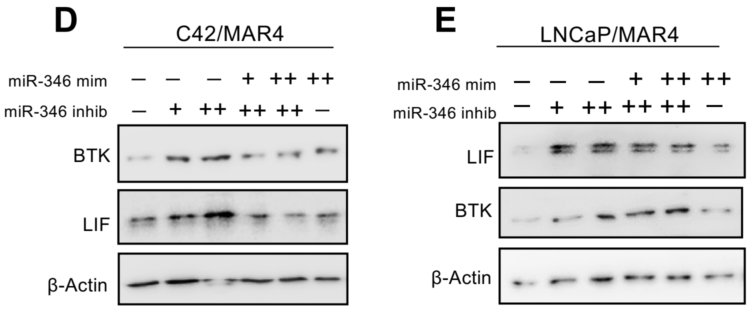

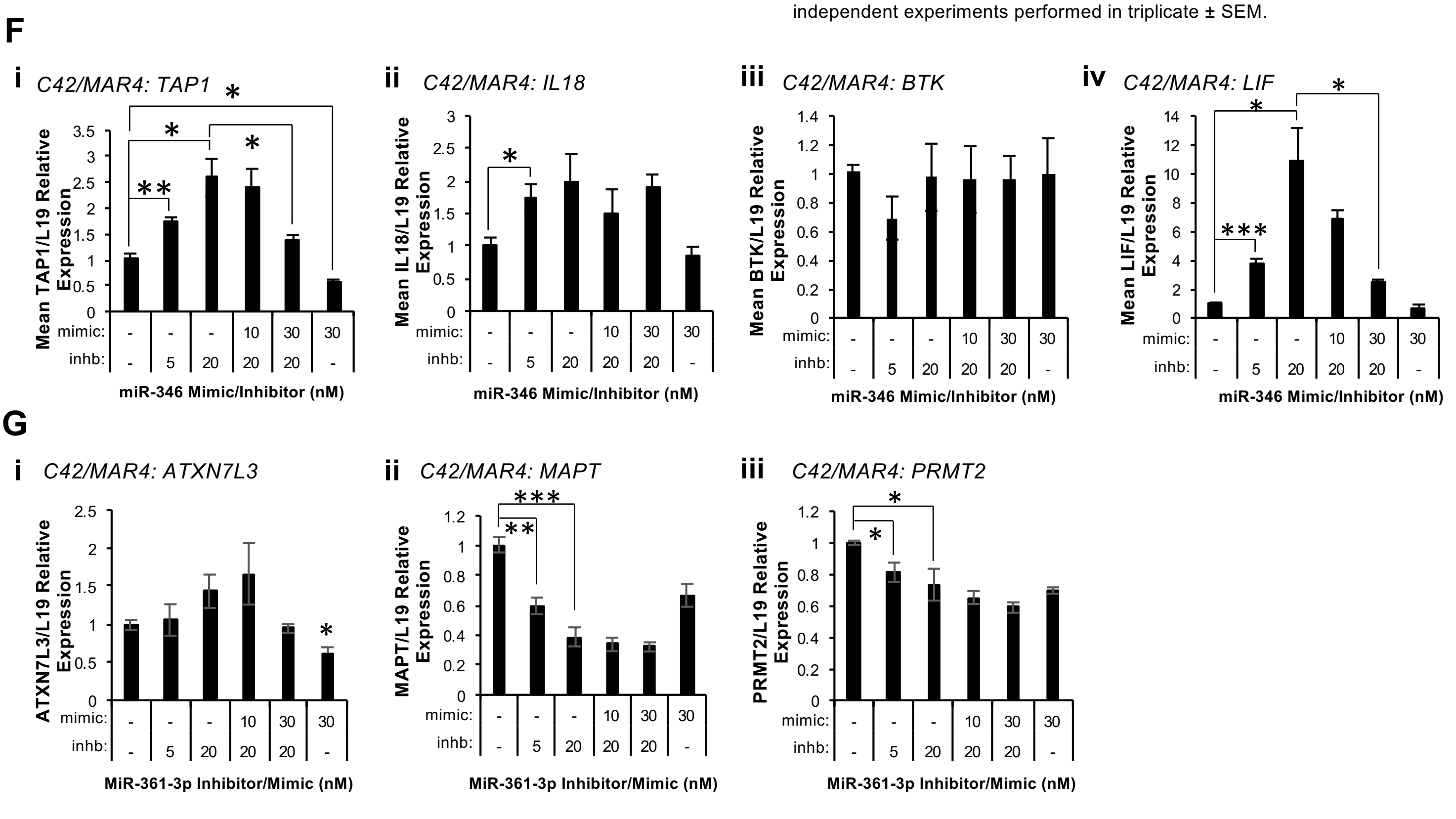

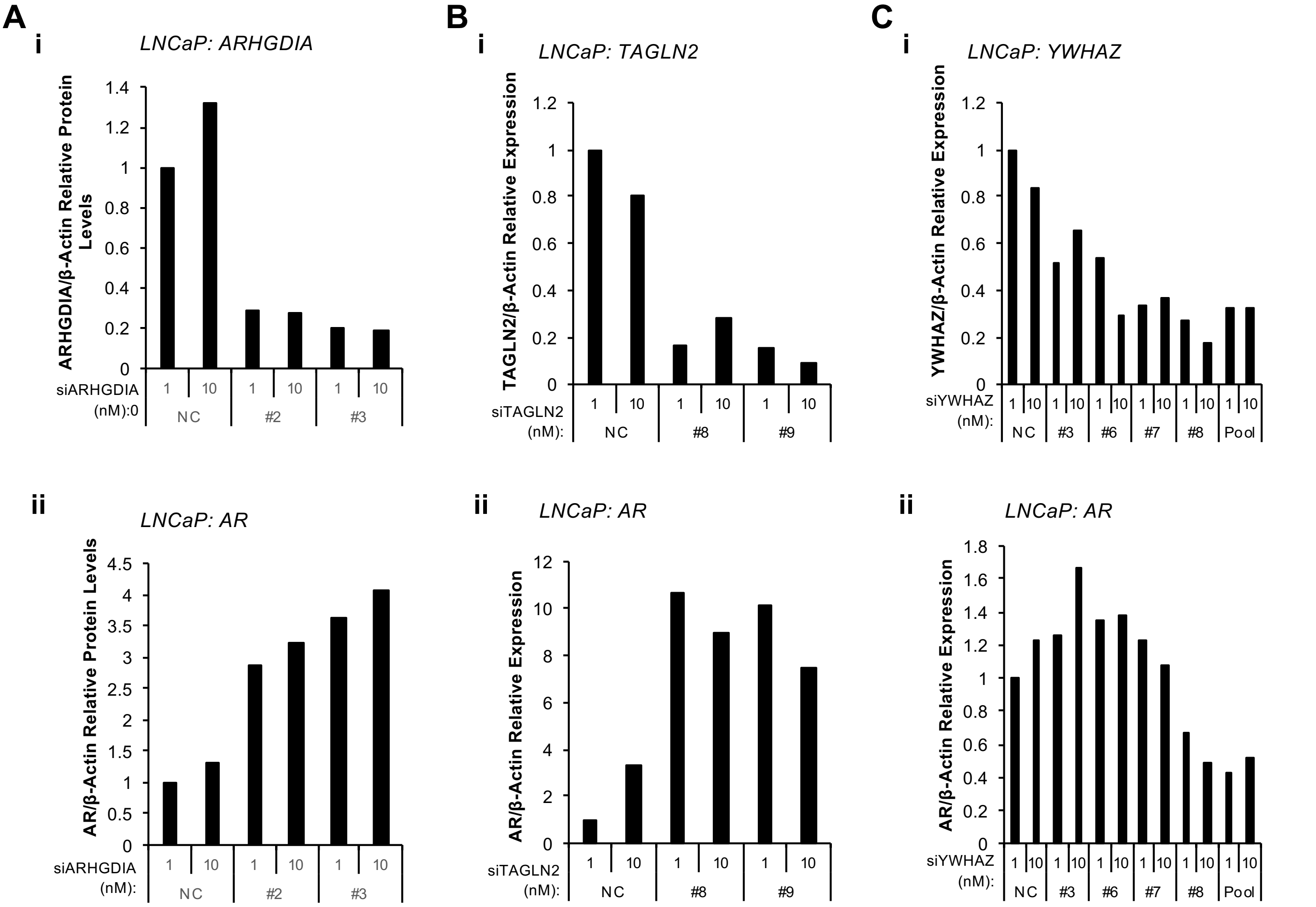

Figure S14: siRNA-Mediated Downregulation of ARHGDIA, TAGLN2 and YWHAZ modulates AR Protein Levels in Prostate Cancer Cells. Quantification of Western blot analysis of ARHGDIA, TAGLN2, YWHAZ and AR protein levels in LNCaP cells transfected with siRNAs (1 or 10nM) targeting *A)* YWHAZ, *B)* TAGLN2 or *C)* ARHGDIA for 72h. β-actin was used as a control for loading. Quantification was performed using ImageJ software.

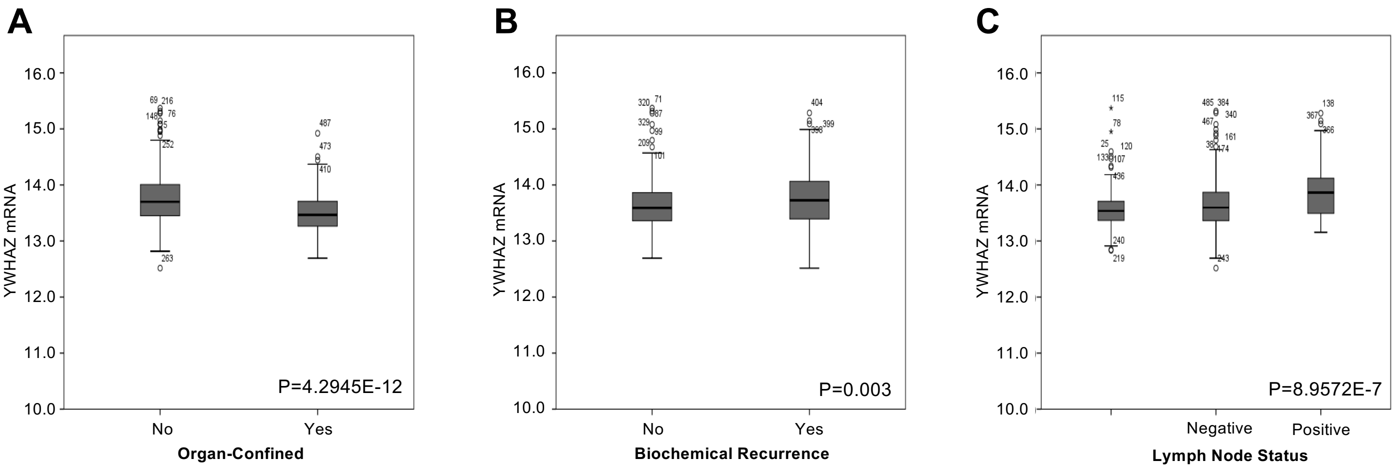

Figure S15: YWHAZ Correlates with Extra-Prostatic Invasion, Biochemical Recurrence and Lymph Node Positivity in Prostate Cancer. YWHAZ mRNA levels were compared between *(A)* organ-confined and non-organ confined prostate tumours, *(B)* tumours of patients with or without biochemical recurrence, and *(C)* between tumours of lymph node-positive and –negative PC patients. Expression data was obtained from the TCGA-PRAD dataset of 333 primary prostate cancers.

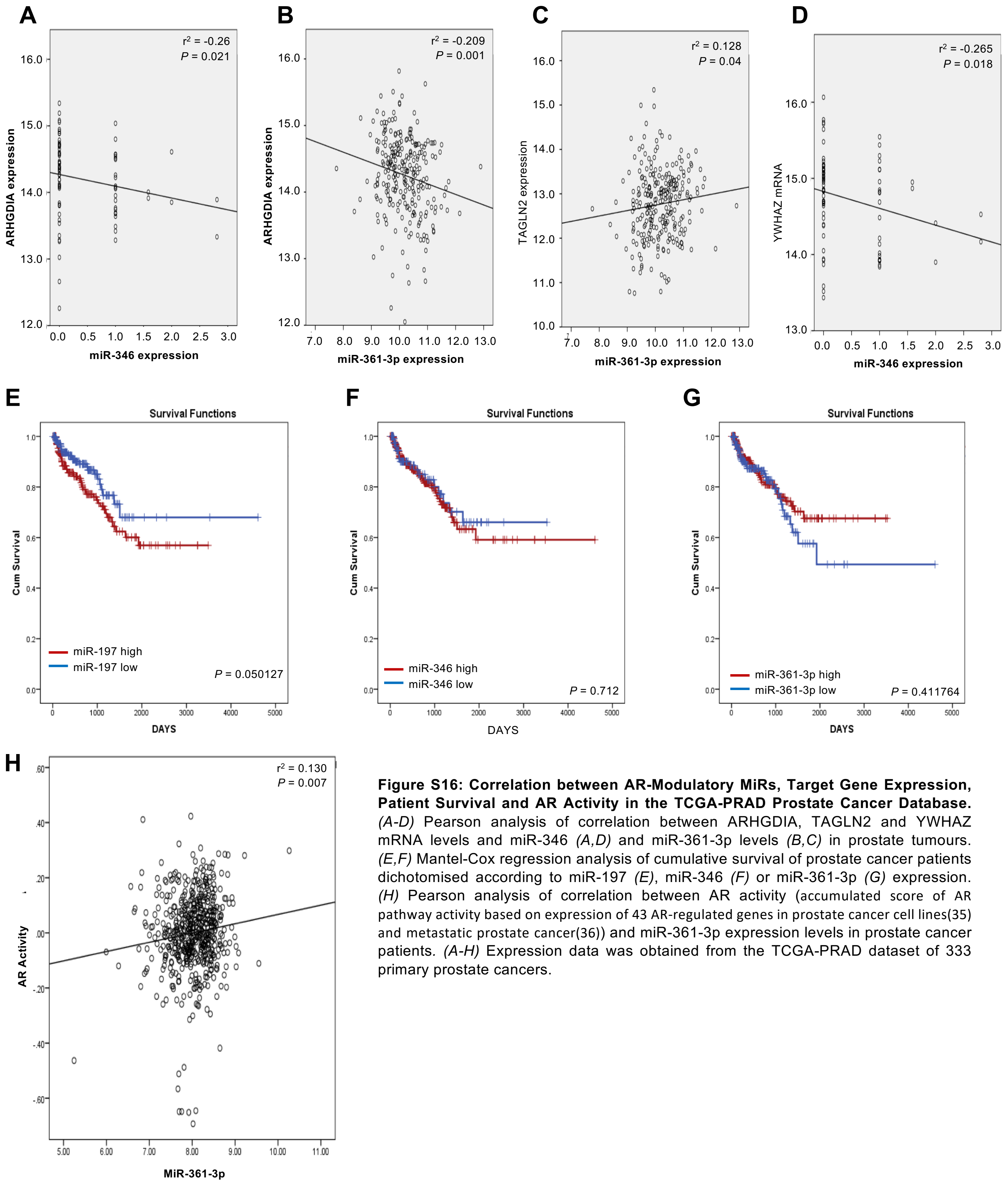

Figure S16: Correlation between AR-Modulatory MiRs, Target Gene Expression, Patient Survival and AR Activity in the TCGA-PRAD Prostate Cancer Database. *(A-D)* Pearson analysis of correlation between ARHGDIA, TAGLN2 and YWHAZ mRNA levels and miR-346 *(A,D)* and miR-361-3p levels *(B,C)* in prostate tumours. *(E,F)* Mantel-Cox regression analysis of cumulative survival of prostate cancer patients dichotomised according to miR-197 *(E)*, miR-346 *(F)* or miR-361-3p *(G)* expression. *(H)* Pearson analysis of correlation between AR activity (accumulated score of AR pathway activity based on expression of 43 AR-regulated genes in prostate cancer cell lines(35) and metastatic prostate cancer(36)) and miR-361-3p expression levels in prostate cancer patients. *(A-H)* Expression data was obtained from the TCGA-PRAD dataset of 333 primary prostate cancers.

Target		Sequence
ACVR1	Forward	5'AATCCCCGAGACGTGGAGTA 3'
	Reverse	5' AATCCCCGAGACGTGGAGTA 3'
AR	Forward	5'-CTGCCCCATCCACGTTGTCCCTGCT-3'
	Reverse	5'GACTCAGATGCTCCAACGCCTCCAC-3'
AR 6.9kb 3'UTR	Forward	5' CCTACAGTGAAGTGCCTGGG 3'
	Reverse	5' TCCCTCCTGCTGCCAGATTA 3'
ARHGDIA	Forward	5'GGATGAGCACTCGGTCAACTA 3'
	Reverse	5'GGCCTCCTTGTACTTTCGCAG 3'
ATXN7L3	Forward	5' CCAGAACCCCAATTCCCCTC 3'
	Reverse	5' CAGGGACCTTGTGCACATCT 3'
BTK	Forward	5' TGTGTTCCACACCTCAGAGC 3'
	Reverse	5' TGCAGTGGAAGGTGCATTCT 3'
DRG1	Forward	5' CAAGACAGGTGATGCTCGAA 3'
	Reverse	5' GTCCCAAAGGTTTCAGGACA 3'
E-Cadherin	Forward	5' ATTTTTCCCTCGACACCCGAT 3'
	Reverse	5' TCCCAGGCGTAGACCAAGA 3'
IL18	Forward	5' TTGACCAAGGAAATCGGCCT 3'
	Reverse	5' CCATACCTCTAGGCTGGCTAT 3'
KLK2	Forward	5' CCTCACGTTCTGGCATCACTT 3'
	Reverse	5' CGGCCAGGTGAGTTCCAA 3'
LIF	Forward	5' ACCTGGACAAGCTATGTGGC 3'
	Reverse	5' ACAGCACGTTGCTAAGGAGG 3'
MAPT	Forward	5' ATGCACCAAGACCAAGAGGG 3'
	Reverse	5' CCGCTGTTGGAGTGCTCTTA 3'
N-Cadherin	Forward	5' AGCCAACCTTAACTGAGGAGT 3'
	Reverse	5' GGCAAGTTGATTGGAGGGATG 3'
PRMT2	Forward	5' AGGGAGTACAGCCAGAGGAG 3'
	Reverse	5' CCACATGGTTTGCCGGAATG 3'
PSA	Forward	5' TTGTCTTCCTCACCCTGTCC 3'
	Reverse	5' AGCTGTGGCTGACCTGAAAT 3'
Slug	Forward	5' CGAACTGGACACACATACAGTG 3'
	Reverse	5' CTGAGGATCTCTGGTTGTGGT 3'
Snail	Forward	5' TCGGAAGCCTAACTACAGCGA 3'
	Reverse	5' AGATGAGCATTGGCAGCGAG
TAGLN2	Forward	5' ATCCCAACTGGTTCCCTAAGAA 3'
	Reverse	5' CCCATCTGTAACCCGATCACG 3'
TAP1	Forward	5' GTTCGAAGCTTTGCCAACGA 3'
	Reverse	5' CAGCTGCCCACCAATGTAGA 3'
TMPRSS2	Forward	5'-AATCGGTGTGTTCGCCTCTAC-3'
	Reverse	5'-GCGGCTGTCACGATCC-3'
TSPAN3	Forward	5' ATGTGTACACGCTCATCCCT 3'
	Reverse	5' CAGGATGATGACAAACGTGGC 3'
TSPAN13	Forward	5' GCGCCCTCAACCTGCTTTA 3'
	Reverse	5' ACTCGGAGACTGGAAATCAGC 3'
TUSC2	Forward	5'GCCGCGGCTCTATGTTCTAT 3'
	Reverse	5' CACGATGCCCTGAGGAATCA 5'
Twist-1	Forward	5' GTCCGCAGTCTTACGAGGAG 3'
	Reverse	5' GCTTGAGGGTCTGAATCTTGCT 3'
Vimentin	Forward	5' AGTCCACTGAGTACCGGAGAC 3'
	Reverse	5' CATTTCACGCATCTGGCGTTC 3'
YWHAZ	Forward	5' CCTGCATGAAGTCTGTAACTGAG 3'
	Reverse	5' GACCTACGGGCTCCTACAACA 3'
ZEB2	Forward	5' GCGATGGTCATGCAGTCAG 3'
	Reverse	5' CAGGTGGCAGGTCATTTTCTT 3'

Table S1: Primer sequences for qRT-PCR

MiR Inhibitor	C42 B score total	LNCaP B score total	Actual sum score	Effect on AR Reporter Luc
hsa-miR-378c	-8.45	-17.27	-25.72	Down
hsa-miR-361-3p	-12.85	-11.57	-24.42	Down
hsa-miR-26b*	-9.78	-12.99	-22.78	Down
hsa-miR-143*	-15.22	-7.05	-22.27	Down
hsa-miR-197	-9.88	-9.38	-19.26	Down
hsa-let-7b*	-10.36	-8.01	-18.37	Down
hsa-miR-203	-3.63	-14.57	-18.20	Down
hsa-miR-346	-11.97	-4.63	-16.60	Down
hsa-miR-593*	-10.52	-5.99	-16.51	Down
hsa-miR-1260	-7.13	-8.47	-15.60	Down
hsa-miR-324-3p	-9.51	-5.71	-15.22	Down
hsa-miR-96*	-1.25	-13.31	-14.56	Down
hsa-miR-16	4.46	10.03	14.49	Up
hsa-miR-621	-4.40	-9.70	-14.10	Down
hsa-miR-1271	-7.82	-6.14	-13.96	Down
hsa-miR-133a	-11.17	-2.76	-13.93	Down
hsa-miR-1227	-5.98	-7.15	-13.14	Down
hsa-miR-548q	-13.18	0.36	-12.83	Down
hsa-miR-516b	11.15	1.68	12.83	Up
hsa-miR-2113	-8.02	-4.45	-12.48	Down
hsa-miR-518a-5p,hsa-miR-527	7.56	3.92	11.48	Up
hsa-miR-664	-3.20	-8.20	-11.40	Down
hsa-miR-195	1.45	9.67	11.12	Up
hsa-miR-610	8.74	2.09	10.83	Up
hsa-miR-1225-3p	-7.59	-3.05	-10.64	Down
hsa-miR-135b*	10.79	-0.18	10.61	Up
hsa-miR-1229	-4.33	-6.26	-10.60	Down
hsa-miR-1914	-7.48	-3.03	-10.51	Down
hsa-miR-744	5.10	5.39	10.49	Up
hsa-miR-766	-7.53	-2.95	-10.48	Down

TableS2: MicroRNA Inhibitors Modulating AR Activity in LNCaP and C42 Cell Lines. Values represent mean score in each cell line. Negative value indicates reduction in AR activity, positive value indicates increase in AR activity. B Score method as previously described (31).

MiR	Location of Seed Binding Site within AR 6.9kb 3'UTR	Type of MiR Site	Sequence of site	V7	V1	V4	V9	V567es	WT
WT-specific miR-346, -361-3p and -197 binding sites									
miR-361-3p	787-793	7mer-m8	CTTGGTCCCTGGGGGCTAGACTGCT	N	N	N	N	n	Y
miR-197	3043-3048	6mer (+pos 9,10)	ACTTAGCTGGTGAGCTAGA	N	N	n	N	n	Y
miR-197	4308-4313	6mer (+pos 9)	GACTCTGCTGGTGACTG	n	n	N	N	n	y
WT/variant-shared miR-346, -361-3p and -197 binding sites (only WT and v567es sites are shared – both in exon 8)									
miR-346	3185-3190	7mer-a1	GCTGGGCAGCAGACAGCTGCCA	n	n	n	n	y	Y
miR-346	6283-6288	6mer (+pos 9,11)	GCAGGAGGGAGCAGACTATGT	n	n	n	n	y	Y
miR-361-3p	407-412	7mer-a1 (+pos 9)	AAGATTATCTGGGGAAAT	N	N	N	N	y	Y
miR-361-3p	5772-5777	8mer	TGTCTCTTAGCCTGGGGAATTAAA	N	N	N	N	y	Y
miR-361-3p	6070-6075	8mer	GAAGTGCCTGGGGGGTTGTCC	N	N	N	N	y	Y
Variant-specific miR-346, -361-3p and -197 binding sites									
miR-361-3p	981-987 (v7 3'UTR), 978-984 (v9 3'UTR), 1268-1274 (v4 3'UTR)	7mer	V7:CCTGACTTGCCTGGGGCCTGTCTTT<br>V9:GACTTGCCTGGGGCCTGTCTTTC<br>V4:CCTGACTTGCCTGGGGCCTGTCTTT	y	n	y	y	n	N
miR-197	24-29 (v567es 3'UTR)	6mer	AAGTCACACATGGTGAGCGTGGA	n	n	n	n	y	N
miR-361-3p	2209-2216 (v9 3'UTR)	8mer	CTGTGTGACCTGGGGCATGAC	n	n	n	y	n	n
miR-346	8353-8358 (v567es 3'UTR)	7mer-a1	AGCAGGAGGGAGCAGACTATGTA	n	n	n	n	y	n

TableS3: Table of miR-346, -361-3p and -197-3p seed complementarity sites in wild-type and variant Androgen Receptor 3'UTRs. MiR-346, -361-3p and -197-3p seed complementarity sites are highlighted in yellow, green and purple, respectively. Y = presence of miR sites, N = absence of miR site. Nucleotide numbers refer to WT AR 6.9kb 3'UTR, unless otherwise stated.

MiR binding location	Strand	Target Gene	Gene Type	Seed Start	Seed End	Seed Type	No of Pca lines (/5)	
NC_RNA	-	RP6-99M1.2	lincRNA	45605615	45605622	7mer-1a	5	DU145, PC3, LNCaP, 22RV1, LAPC4
Intron	-	Intron	Intron	133013410	133013418	8mer-1a	5	DU145, PC3, LNCaP, 22RV1, LAPC4
3_UTR	-	PPP2CA	Prot coding	133532948	133532955	7mer-1a	5	DU145, PC3, LNCaP, 22RV1, LAPC4
CDS	+	MYL6	Prot coding	56553804	56553811	7mer-1a	5	DU145, PC3, LNCaP, 22RV1, LAPC4
CDS	-	TRAPPC1	Prot coding	7835098	7835105	7mer-1a	5	DU145, PC3, LNCaP, 22RV1, LAPC4
3_UTR	-	HNRNPK	Prot coding	86584688	86584695	7mer-m8	4	PC3, LNCaP, 22RV1, LAPC4
3_UTR	-	YWHAZ	Prot coding	101932896	101932904	8mer-1a	4	PC3, LNCaP, 22RV1, LAPC4
Promoter	-	YWHAZP2	Promoter	127314982	127314990	8mer-1a	4	PC3, LNCaP, 22RV1, LAPC4
CDS	-	TMED4	Prot coding	44621091	44621098	7mer-1a	4	PC3, LNCaP, 22RV1, LAPC4
3_UTR	-	TUBA1B	Prot coding	49525117	49525124	7mer-1a	4	DU145, PC3, 22RV1, LAPC4
3_UTR	-	F11R	Prot coding	160966039	160966046	7mer-1a	4	PC3, LNCaP, 22RV1, LAPC4
3_UTR	-	ENO1	Prot coding	8938683	8938690	7mer-1a	4	DU145, PC3, 22RV1, LAPC4
3_UTR	-	EIF4G2	Prot coding	10825904	10825911	7mer-1a	4	DU145, PC3, 22RV1, LAPC4
NC_RNA	+	SNORA81	snoRNA	186504493	186504500	7mer-1a	4	DU145, PC3, 22RV1, LAPC4
3_UTR	-	YWHAZ	Prot coding	101931200	101931208	8mer-1a	4	DU145, PC3, 22RV1, LAPC4
3_UTR	-	TXN2	Prot coding	36863472	36863479	7mer-m8	4	PC3, LNCaP, 22RV1, LAPC4
CDS	-	AARS	Prot coding	70316576	70316583	7mer-m8	4	DU145, PC3, 22RV1, LAPC4
3_UTR	+	NFIC	Prot coding	3465087	3465094	7mer-m8	4	DU145, PC3, 22RV1, LAPC4
CDS	+	HIST2H2AC	Prot coding	149858916	149858923	7mer-1a	4	PC3, LNCaP, 22RV1, LAPC4
NC_RNA	-	LINC00657	lincRNA	34635627	34635634	7mer-1a	3	PC3, 22RV1, LAPC4

TableS4: Table of miR-346 binding site identified by AGO-HITS-PAR-CLIP in at least 3 of 5 prostate cancer cell lines. Seed sequence = GUCUGCC. AGO-PAR-CLIP-seq data represented is extracted from prior published data[25]

Chrom	Strand	Target Gene	Gene Type	Seed Start	Seed End	Seed Type	No of Pca lines (/5)	
chr16	+	Intergenic	Intergenic	33964106	33964113	7mer-1a	5	DU145, PC3, LNCaP, 22RV1, LAPC4
chr1	+	RNU1-4	snRNA	17067146	17067153	7mer-1a	5	DU145, PC3, LNCaP, 22RV1, LAPC4
chr19	-	CTC-457E21.9	antisense	22877682	22877689	7mer-1a	5	DU145, PC3, LNCaP, 22RV1, LAPC4
chr2	+	RPLP0P6	pseudogene	38709237	38709245	8mer-1a	5	DU145, PC3, LNCaP, 22RV1, LAPC4
chr14	+	RNU1-27P	snRNA	35016055	35016062	7mer-1a	5	DU145, PC3, LNCaP, 22RV1, LAPC4
chr2	-	Intron	Intron	133012342	133012349	7mer-1a	5	DU145, PC3, LNCaP, 22RV1, LAPC4
chrX	-	MIR361	miRNA	85158660	85158668	8mer-1a	5	DU145, PC3, LNCaP, 22RV1, LAPC4
chr7	-	ACTB	Prot coding	5568195	5568202	7mer-1a	5	DU145, PC3, LNCaP, 22RV1, LAPC4
chrX	-	BCAP31	Prot coding	152966277	152966284	7mer-1a	5	DU145, PC3, LNCaP, 22RV1, LAPC4
chrY	+	Intergenic	Intergenic	10035774	10035781	7mer-m8	4	DU145, PC3, 22RV1, LAPC4
chr1	-	TAGLN2	Prot coding	159888415	159888422	7mer-1a	4	DU145, PC3, 22RV1, LAPC4
chr10	-	PSAP	Prot coding	73577081	73577088	7mer-1a	4	DU145, PC3, 22RV1, LAPC4
chr11	-	GANAB	Prot coding	62393053	62393060	7mer-1a	4	DU145, PC3, 22RV1, LAPC4
chr8	+	NDUFB9	Prot coding	125555482	125555489	7mer-m8	4	DU145, PC3, 22RV1, LAPC4
chr15	-	PKM	Prot coding	72491439	72491446	7mer-1a	4	DU145, PC3, 22RV1, LAPC4
chr1	-	ICMT	Prot coding	6284648	6284655	7mer-1a	4	DU145, PC3, 22RV1, LAPC4
chr3	+	EIF4G1	Prot coding	184053040	184053047	7mer-m8	4	DU145, PC3, 22RV1, LAPC4
chr7	-	ACTB	Prot coding	5567185	5567192	7mer-1a	4	DU145, PC3, 22RV1, LAPC4
chr3	+	PSMD2	Prot coding	184026763	184026771	8mer-1a	4	DU145, PC3, 22RV1, LAPC4
chr7	-	ACTB	Prot coding	5566890	5566897	7mer-1a	4	DU145, PC3, 22RV1, LAPC4
chr10	-	TIMM23	Prot coding	51592233	51592240	7mer-1a	4	DU145, PC3, 22RV1, LAPC4
chr11	-	GANAB	Prot coding	62392945	62392953	8mer-1a	4	DU145, PC3, 22RV1, LAPC4
chr11	-	CFL1	Prot coding	65622546	65622553	7mer-m8	4	DU145, PC3, 22RV1, LAPC4
chr11	-	BCL9L	Prot coding	118768876	118768883	7mer-1a	4	DU145, PC3, 22RV1, LAPC4
chr12	-	ATP5B	Prot coding	57039662	57039669	7mer-m8	4	DU145, PC3, 22RV1, LAPC4
chr16	+	Intergenic	Intergenic	33963425	33963432	7mer-m8	4	DU145, PC3, 22RV1, LAPC4
chr11	+	MALAT1	lincRNA	65269654	65269661	7mer-m8	4	DU145, PC3, 22RV1, LAPC4
chr11	+	MALAT1	lincRNA	65269026	65269033	7mer-1a	4	DU145, PC3, 22RV1, LAPC4
chr3	-	RPL10AP6	pseudogene	61728531	61728538	7mer-1a	4	DU145, PC3, 22RV1, LAPC4
chr19	+	RPS19	Prot coding	42373164	42373171	7mer-1a	4	DU145, PC3, 22RV1, LAPC4
chr6	+	RPL10A	Prot coding	35437197	35437205	8mer-1a	3	DU145, PC3, 22RV1

TableS5: Table of miR-361-3p binding site identified by AGO-HITS-PAR-CLIP in at least 3 of 5 prostate cancer cell lines. Seed sequence = CCCCCAG. AGO-PAR-CLIP-seq data represented is extracted from prior published data[25]

MiR Binding Location	Strand	Target Gene	Gene Type	Seed Start	Seed End	Seed Type	No of Pca lines interaction found in (/5)	
Intron	-	Intron	Intron	91852823	91852830	7mer-1a	5	DU145, PC3, LNCaP, 22RV1, LAPC4
NC_RNA	+	RP11-25K21.6	Processed transcript	161500916	161500923	7mer-m8	5	DU145, PC3, LNCaP, 22RV1, LAPC4
Promoter	-	MTRNR2L2	Promoter	79947295	79947302	7mer-m8	5	DU145, PC3, LNCaP, 22RV1, LAPC4
Promoter	-	MTRNR2L8	Promoter	10531164	10531171	7mer-m8	5	DU145, PC3, LNCaP, 22RV1, LAPC4
3_UTR	-	RPS2	Prot coding	2012193	2012200	7mer-1a	5	DU145, PC3, LNCaP, 22RV1, LAPC4
Intergenic	-	Intergenic	Intergenic	13060359	13060366	7mer-1a	5	DU145, PC3, LNCaP, 22RV1, LAPC4
NC_RNA	+	MIR27B	miRNA	97847758	97847765	7mer-1a	4	PC3, LNCaP, 22RV1, LAPC4
3_UTR	+	ATP6V0E1	Prot coding	172461561	172461568	7mer-1a	4	PC3, LNCaP, 22RV1, LAPC4
CDS	-	KDELR1	Prot coding	48893712	48893719	7mer-1a	5	DU145, PC3, LNCaP, 22RV1, LAPC4
CDS	-	PABPC1	Prot coding	101727758	101727765	7mer-1a	5	DU145, PC3, LNCaP, 22RV1, LAPC4
3_UTR	-	RPLP0	Prot coding	120636964	120636971	7mer-1a	5	DU145, PC3, LNCaP, 22RV1, LAPC4
Intergenic	+	Intergenic	Intergenic	70108656	70108663	7mer-1a	4	PC3, LNCaP, 22RV1, LAPC4
CDS	-	AP3M1	Prot coding	75897975	75897982	7mer-m8	4	PC3, LNCaP, 22RV1, LAPC4
NC_RNA	+	C17orf76-AS1	Processed transcript	16344587	16344595	8mer-1a	3	LNCaP, 22RV1, LAPC4
3_UTR	-	DDX39B	Prot coding	31498080	31498087	7mer-m8	4	PC3, LNCaP, 22RV1, LAPC4
3_UTR	-	TMEM55B	Prot coding	20926298	20926305	7mer-1a	4	PC3, LNCaP, 22RV1, LAPC4
3_UTR	-	SIGMAR1	Prot coding	34635653	34635660	7mer-m8	4	PC3, LNCaP, 22RV1, LAPC4
CDS	-	LRRC58	Prot coding	120050163	120050170	7mer-1a	4	PC3, LNCaP, 22RV1, LAPC4
CDS	-	FUT10	Prot coding	33310784	33310791	7mer-1a	4	PC3, LNCaP, 22RV1, LAPC4
NC_RNA	-	CEP164P1	pseudogene	45527364	45527372	8mer-1a	4	PC3, LNCaP, 22RV1, LAPC4
CDS	-	DICER1	Prot coding	95569746	95569753	7mer-m8	4	PC3, LNCaP, 22RV1, LAPC4
Promoter	-	PKD1	Promoter	2156693	2156700	7mer-m8	4	PC3, LNCaP, 22RV1, LAPC4
Intron	+	Intron	Intron	145277316	145277324	8mer-1a	4	PC3, LNCaP, 22RV1, LAPC4
3_UTR	-	NDUFC1	Prot coding	140211202	140211209	7mer-1a	4	PC3, LNCaP, 22RV1, LAPC4
Intron	+	Intron	Intron	137201694	137201701	7mer-1a	2	PC3, LNCaP
Intron	-	Intron	Intron	85982273	85982280	7mer-m8	2	PC3, LNCaP
Intron	+	Intron	Intron	141282104	141282111	7mer-m8	2	PC3, LNCaP
Intergenic	-	Intergenic	Intergenic	133036745	133036752	7mer-1a	2	PC3, LNCaP
Intergenic	+	Intergenic	Intergenic	36794244	36794251	7mer-1a	2	PC3, LNCaP

TableS6: Table of miR-197-3p binding site identified by AGO-HITS-PAR-CLIP in at least 2 of 5 prostate cancer cell lines. Seed sequence = UCACCAC. AGO-PAR-CLIP-seq data represented is extracted from prior published data[25]

Chrom	Sequence Start	Sequence end	MiR Binding Location	Strand	Gene ID	Gene Type	Seed Start	Seed End	Seed_Sequence	Seed_Type	miRNA_Family	Pca Cell Lines
chr17	79826443	79826496	3_UTR	-	ARHGDIA	Prot coding	79826460	79826467	CCCCCAG	7mer-m8	miR-361-3p	DU145, PC3, 22RV1
chr17	79826657	79826726	3_UTR	-	ARHGDIA	Prot coding	79826684	79826691	CCCCCAG	7mer-m8	miR-361-3p	22RV1, PC3
chr17	79825598	79825911	3_UTR	-	ARHGDIA	Prot coding	79825795	79825803	CCCCCAG	8mer-1a	miR-361-3p	22RV1
chr17	79826487	79826577	3_UTR	-	ARHGDIA	Prot coding	79826567	79826574	CCCCCAG	7mer-1a	miR-361-3p	LAPC4
chr17	79826142	79826294	3_UTR	-	ARHGDIA	Prot coding	79826189	79826196	GUCUGCC	7mer-1a	miR-346	22RV1, LAPC4, PC3
chr17	79825598	79825911	3_UTR	-	ARHGDIA	Prot coding	79825660	79825668	GUCUGCC	8mer-1a	miR-346	22RV1, LAPC4, PC3
chr1	159888176	159888488	3_UTR	-	TAGLN2	Prot coding	159888415	159888422	CCCCCAG	7mer-1a	miR-361-3p	DU145, PC3, 22RV1, LAPC4
chr1	159887984	159888106	3_UTR	-	TAGLN2	Prot coding	159888072	159888079	UCACCAC	7mer-m8	miR-197	DU145, PC3, 22RV1, LAPC4
chr8	107710117	107710173	NC_RNA	-	TAGLN2P1	pseudogene	107710133	107710140	UCACCAC	7mer-m8	miR-197	LAPC4, PC3
chr1	159890189	159890289	CDS	-	TAGLN2	Prot coding	159890192	159890199	UCACCAC	7mer-m8	miR-197	22RV1
chr8	101932877	101932928	3_UTR	-	YWHAZ	Prot coding	101932896	101932904	GUCUGCC	8mer-1a	miR-346	PC3, LNCaP, 22RV1, LAPC4
chr2	127314952	127315002	Promoter	-	YWHAZP2	Promoter	127314982	127314990	GUCUGCC	8mer-1a	miR-346	PC3, LNCaP, 22RV1, LAPC4
chrX	63832420	63832459	Promoter	-	YWHAZP7	Promoter	63832439	63832447	GUCUGCC	8mer-1a	miR-346	LNCaP, 22RV1
chr8	101931155	101931216	3_UTR	-	YWHAZ	Prot coding	101931200	101931208	GUCUGCC	8mer-1a	miR-346	DU145, PC3, 22RV1, LAPC4
chr8	101964268	101964298	3_UTR	-	YWHAZ	Prot coding	101964283	101964290	GUCUGCC	7mer-1a	miR-346	PC3

Table S7: MiR-346, -361-3p and -197-3p Target ARHGDIA, TAGLN2 and YWHAZ in Prostate Cancer Cells. Binding sites for miR-346, -361-3p and -197-3p in promoter, coding region and 3'UTRs of ARHGDIA, TAGLN2 and YWHAZ, as identified by AGO-PAR-CLIP across five prostate cancer cell lines.
